# Exploring the pathogenesis and key genes associated of acute myocardial infarction complicated with Alzheimer’s disease

**DOI:** 10.1038/s41598-024-52094-4

**Published:** 2024-01-16

**Authors:** Chaosheng Liu, Fuzhi Pan, Zhiyu Sun, Ziyu Chen, Junjie Wang

**Affiliations:** 1 Department of Cardiology, The First Affiliated Hospital of Dalian Medical University, Dalian, Liaoning China; 2 Department of Medical Image Science, Liaoning Cancer Hospital, Shenyang, Liaoning China; 3 Department of Cardiology, Dalian Friendship Hospital, Dalian, Liaoning China; 4 Department of Gynecology and Obstetrics, The First Affiliated Hospital of Dalian Medical University, Dalian, Liaoning China

**Keywords:** Cell biology, Cardiology

## Abstract

Despite mounting evidence linking Acute Myocardial Infarction (AMI) to Alzheimer’s disease (AD), the shared mechanism of these two conditions’ occurrence remains unclear. This research aims to delve deeper into the molecular process of the occurrence of the two diseases. We retrieved the gene expression profiles of AD (GSE5281) and AMI (GSE66360) from the Gene Expression Omnibus database. Then, a total of 22 common differentially expressed genes (DEGs) including one downregulated gene and 21 upregulated genes were chosen for further analysis. Following the discovery of the common DEGs between AMI and AD, we performed protein–protein interaction analysis and hub gene identification analysis. Next, ten important hub genes were identified. Additionally, the key genes were identified by the least absolute shrinkage and selection operator and support vector machine‐recursive feature elimination and multivariable logistic regression analysis. The BCL6 was identified to be the most connected with AMI and AD. Finally, the BCL6 gene was validated in the GSE40680 (AMI) and GSE122063 (AD) datasets. Our research indicates that AMI and AD share a comparable pathophysiology. The Hub genes, especially BCL6, were essential in developing AMI and AD. In addition, these hub genes and shared pathways can offer fresh perspectives for additional mechanism investigation.

## Introduction

Chest pain is the second most frequent reason people attend the emergency department (ED) in the United States after injuries, accounting for over 7 million visits of all ED visits^1^. Therefore, chest pain necessitates a comprehensive clinical evaluation and continues to be a diagnostic difficulty in the ED and outpatient environment^2^. Although noncardiac causes of chest pain are common, coronary artery disease (CAD) affects more than 18.2 million adults in the United States. With more than 365,000 deaths a year, it continues to be the primary cause of death for both men and women^3^. Therefore, cardiovascular disease significantly negatively impacts both health and the economy domestically and internationally. In the field of cardiovascular medicine, an acute myocardial infarction (AMI) is a medical disorder that can cause life-threatening symptoms such as abrupt cardiac death, cardiogenic shock, and malignant arrhythmias. Moreover, it is among the main reasons for sudden cardiac death^2^. The prognosis of individuals with AMI is greatly affected by early diagnosis. While the first ECG is necessary for the evaluation, other aids such as the history, examination, and biomarkers are also important. As a result, we are attempting to uncover novel biomarkers in order to enhance the diagnosis rate of early AMI.

Dementia is a clinical syndrome characterized by a progressive decline in two or more cognitive domains, such as memory, language, executive and visuospatial function, personality, and behavior, resulting in the inability to perform instrumental and/or fundamental everyday activities. Up to 80% of dementia diagnoses are due to Alzheimer’s disease (AD), making it the most frequent cause of dementia^4^. Alzheimer’s disease was officially classified as the sixth-highest cause of death in the United States in 2019, with 121,499 fatalities from the condition, according to official death certificates. Alzheimer’s dementia is thought to affect 6.7 million Americans 65 years of age and older as of right now. Without medical advancements to prevent, slow down, or treat AD, this population could increase to 13.8 million by 2060^5^. Further, due to their high frequency and modifiability, vascular risk factors are widely acknowledged as the most critical group of risk factors for brain health^6^. Numerous variables that raise the risk of cardiovascular disease, such as diabetes and hypertension, also raise the risk of dementia^7–9^. Consequently, it seems that AD might be associated with AMI. Furthermore, with blood biomarkers appearing to be within reach, the advances in biomarker detection have completely rethought the approach to diagnosing Alzheimer’s disease prior to clinical symptomatology. Therefore, it makes sense and is required to design a novel biological diagnostic model for preclinical AD diagnosis.

In this study, we first conducted gene co-expression analysis on AMI and AD. We found that compared to normal individuals, 22 genes, including 21 upregulated genes and one downregulated gene, exhibit the same expression trend in patients with AMI and AD. Based on the genes above, we conducted enrichment analysis and obtained ten essential genes to explore disease correlation further. We have successfully constructed bioinformatics models for AMI and AD patients based on the least absolute shrinkage and selection operator (LASSO), support vector machine recursive feature elimination (SVM-RFE) analysis (SVM-RFE) and multivariable logistic regression analysis. The BCL6 gene is closely associated with AMI and AD patients. This indicates that neither AMI nor AD exists separately, providing new ideas and targets for diagnosing and treating diseases, including AMI and AD, which undoubtedly had specific significance.

## Methods

### Date preparation

The microarray datasets for AMI, AD, and normal controls were obtained from the Gene Expression Omnibus (GEO) database. The gene expression profiles of 50 controls and 49 AMI patients were included in the GSE66360 dataset. Additionally, the GSE48060 was chosen as the validation dataset; it comprises 31 AMI patients and 21 normal people. 87 AD patients and 74 healthy controls were included in the analysis of the GSE5281 dataset. Additionally, from GSE122063, which served as the validation dataset, 56 AD patients and 44 controls were chosen. Since these gene expression profiles were taken from a publicly available, free internet database, our research did not need permission from the Ethics Committee.

### Identification of differentially expressed genes (DEGs) in AMI and AD

After the data downloaded from the GEO database, the primary data was annotated to form an expression matrix, each probe was matched to their homologous gene symbols, and the repeated gene symbols in the matrix were excluded. Next, the matrix was then normalized using the RMA method^10^. Then, the R software’s “limma” package was used to analyze the differential expression of the AMI and AD samples. DEGs were defined as genes with *p* < 0.05 and (|log fold change (FC)|≥ 1. As the demographics of age, sex, and weight of the individuals were not available from the primary data, we were unable to adjust the data for subgroup analysis based on these factors. DEG heat maps and volcano plots were produced using the “pheatmap” and “ggplot2” packages. Following our use of these screening parameters, two sets of DEGs were found. With the “venn” package, these DEGs were analyzed to determine the intersecting genes between the AMI and AD^11^. Further analysis was conducted using these common genes that intersected.

### Functional enrichment analysis for common DEGs

The functional enrichment analysis was carried out in three GO domains: biological process (BP), cellular component (CC), and molecular function (MF)^12^. Databases of pathways involving illnesses, medications, chemicals, and biological processes can be found in the KEGG database^13–16^. In order to ascertain the biological roles of the genes and related pathways, the enrichment analysis was performed using the R package “clusterProfiler.” A *p* value of less than 0.05 was deemed statistically significant.

### Construction of protein–protein interaction (PPI) network and identification of hub genes

We created a PPI network^17^ using the Search Tool for the Retrieval of Interacting Genes (STRING) (http://string-db.org/) in order to investigate the interactions between the common genes that were found above in more detail. Cytoscape 3.9.1 was used to display the findings. The proteins were represented by nodes in the network result, while the interactions between proteins were shown by lines. We installed the MCODE and cytoHubba plugin, which can be downloaded for free via Cytoscape software, after finding the hub genes among these common genes in order to analyze the correlation among these genes^18,19^. Additionally, genes for functional tests were prioritized by GeneMANIA (http://genemania.org/)^20^.

### Establishment and evaluation of the prognosis model

In biomedical research, it is important to select the variables most associated with the studied outcome and to determine the strength of this association. The “glmnet” package was used to obtain the LASSO algorithm to minimize the dimensionality of the data^21^. Besides, using an “SVM” package, a support vector machine-recursive feature elimination (SVM-RFE) model was created, and the average misjudgment rates of their tenfold cross-validations were compared^22^. Additionally, the impact of confounding factors was lessened by applying the multivariable logistic regression analysis^23^. A nomogram is primarily used to summarise the specific and statistically important parameters obtained from multivariable logistic regression analysis as a simple, two-dimensional graphic^24^. The “rms” package in R was used to estimate AMI and AD risk. A diagnostic nomogram model for AMI and AD was created by summarising logistic regression’s independent components.

### Gene set variation analysis (GSVA) analysis

Gene set enrichment analysis (GSEA) is a popular method for condensing data from gene expression profiles into pathways or signature summaries. More accurately than GSEA, gene set variation analysis (GSVA) may identify minute changes in pathway activity in sample populations. GSVA is the basis for developing biological models that are centered around pathways^25^. The GO set served as the study’s background set, and each marker gene was subjected to GSVA analysis using the R software’s “GSVA” package. In the event that t > 0 signifies the activation of a function in the high-expression group, t < 0 implies that this activation is present in the low-expression group.

### Construction of ceRNA network

For the ceRNA network, based on the BCL6 gene, mRNA-miRNA interaction pairings were predicted using the starBase database (http://starbase.sysu.edu.cn/). In the meantime, the human miRNA sequences were retrieved from 3 miRNA target prediction databases: miRbase (https://www.mirbase.org/cgi-bin/browse.pl), TargetScan (http://www.targetscan.org/vert_72/), miRDB (http://mirdb.org/), and we extract the results with three occurrences in the three databases as our prediction result. Besides, lncRNAs and miRNAs were analyzed based on the lncRNA target prediction database: Spongescan (http://spongescan.rc.ufl.edu). With a critical score threshold of 140, the Miranda online predicts the nucleic acid binding between mRNA and miRNA (http://www.bioinformatics.com.cn/local_miranda_miRNA_target_prediction_120). After that, with the help of the Cytoscape (version 3.9.1), we screened miRNA-lncRNAs in starBase for projected miRNAs and created a ceRNA network of mRNA-miRNA-lncRNAs^26^.

## Results

### Differential expression analysis of DEGs in AMI and AD

**Figure 1:**
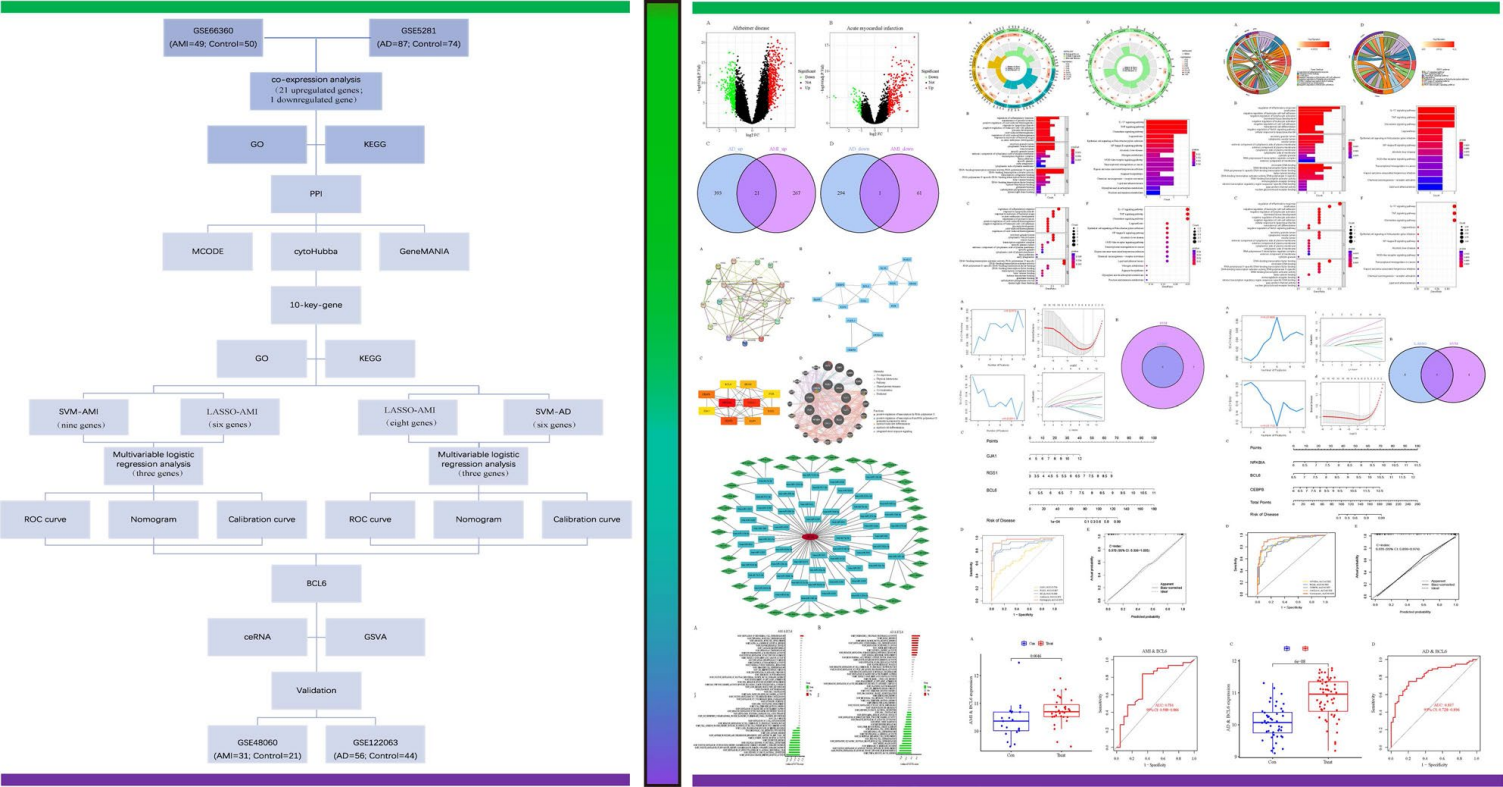
The flow chart of this study.

**Figure 2:**
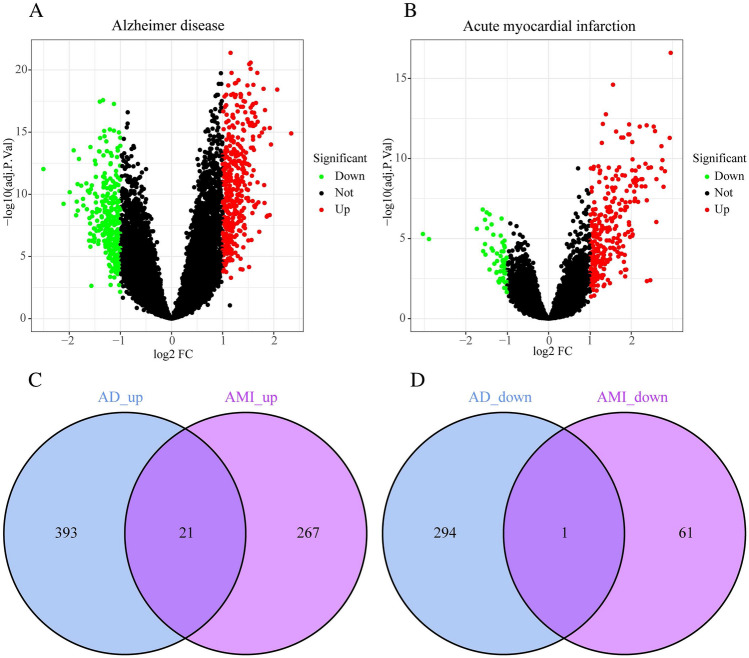
Differentially expressed genes. (**A**) The volcano plot for AD. (**B**) The volcano plot for AMI. (**C**) Upregulated genes between AMI and AD. (**D**) Downregulated genes between AMI and AD.

Figure 1 displays the flowchart for this article. In the GSE5281 dataset, we found 414 upregulated genes and 295 downregulated genes for AD patients after excluding the batch effect (Fig. 2A) (Supplementary Table 1). The GSE66360 dataset revealed 350 genes with substantial changes for AMI patients, including 288 upregulated and 62 downregulated genes (Fig. 2B) (Supplementary Table 2). Based on this, we compared the differentially expressed genes in AMI and AD patients, ultimately obtaining a total of 22 differentially expressed genes, consisting of 21 upregulated genes and 1 downregulated gene (Fig. 2C,D).

### Functional enrichment analysis

**Figure 3:**
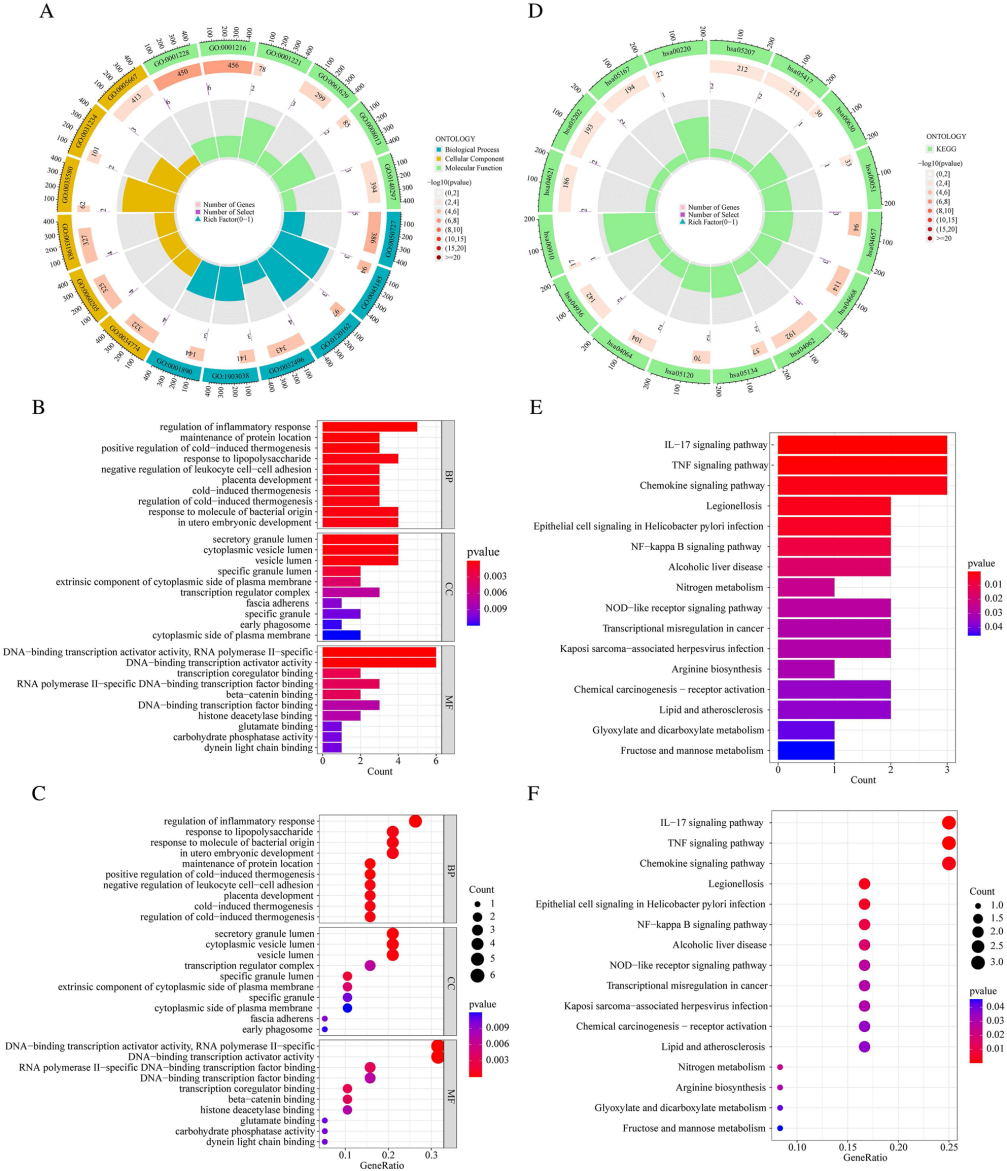
Functional enrichment analysis. (**A**–**C**) The circle plot (**A**), bar plot (**B**), and bubble plot (**C**) via GO analysis. (**D**–**F**) The circle plot (**D**), bar plot (**E**), and bubble plot (**F**) via KEGG analysis.

In order to obtain a more comprehensive understanding of the role of DEGs, enrichment studies of KEGG and GO pathways were made. Changes in BP mainly included inflammatory reactions (e.g., regulation of inflammatory response) and signal transduction (e.g., maintenance of protein location and positive regulation of cold-induced thermogenesis). Besides, the changes in CC were notably focused on cell secretion, such as secretory granule lumen, cytoplasmic vesicle lumen, and vesicle lumen. Moreover, in the MF section, changes were significant in DNA-binding transcription activator activity, RNA polymerase II-specific (Fig. 3A–C) (Supplementary Table 3). In particular, changes in the KEGG pathway mainly were enriched in several immune-related pathways (IL-17 signaling pathway, TNF signaling pathway, and Chemokine signaling pathway) (Fig. 3D–F) (Supplementary Table 4).

### PPI network analysis and hub gene selection

**Figure 4:**
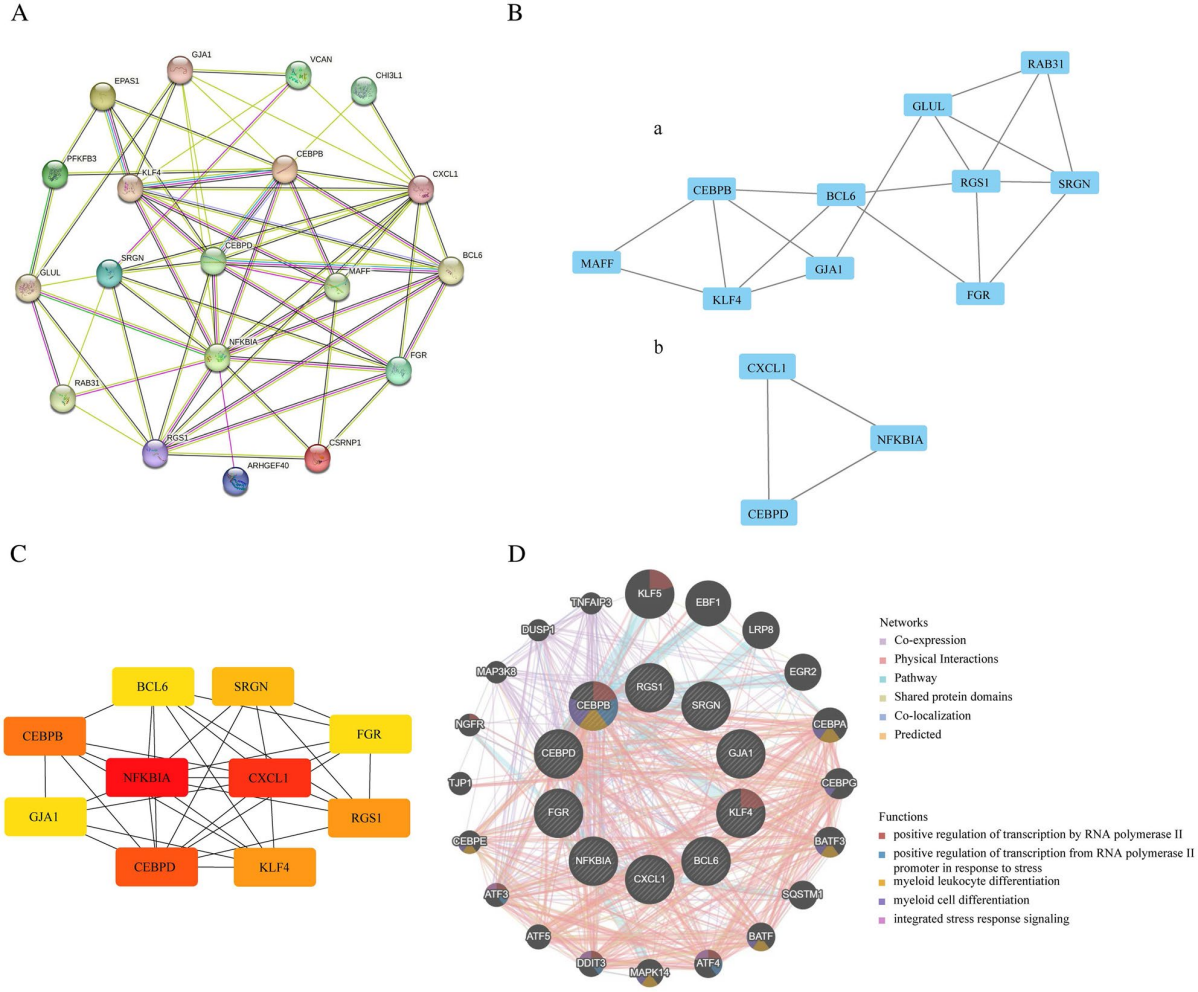
PPI analysis. (**A**) PPI network of the DEGs. (**B**) Two significant gene clustering modules via MCODE. (**C**) Ten hub genes via cytoHubba. (**D**) The gene–gene interaction network for hub genes via the GeneMANIA database.

To identify hub genes and show potential interactions between proteins encoded by common DEGs, STRING screened the DEGs’ PPI network, which had 19 nodes and 64 edges (Fig. 4A). To find important clustering modules, module analysis was carried out using the Cytoscape plug-in MCODE. Two modules were retrieved from the PPI network constructed using common DEGs. Module 1 included ten nodes and 18 edges, and module 2 with three nodes and three edges (Fig. 4B). Besides, through the seven plug-in cytoHubba algorithms, we calculated the top ten hub genes for the following analysis (Supplementary Table 5), and the relationship among ten hub genes was displayed in Fig. 4C.

GeneMANIA conducted PPI analysis of the ten hub genes and their twenty interacting genes to predict correlations between colocalization, pathways, shared protein domains, coexpression, and prediction (Fig. 4D). The inner circle contains the hub genes, and the outside circle contains the predicted genes. As shown in Fig. 4D, the network illustrates that these genes were mainly enriched in positive regulation of transcription by RNA polymerase II, positive regulation of transcription from RNA polymerase II promoter in response to stress, and myeloid leukocyte differentiation.

### Hub gene functional enrichment analysis

**Figure 5:**
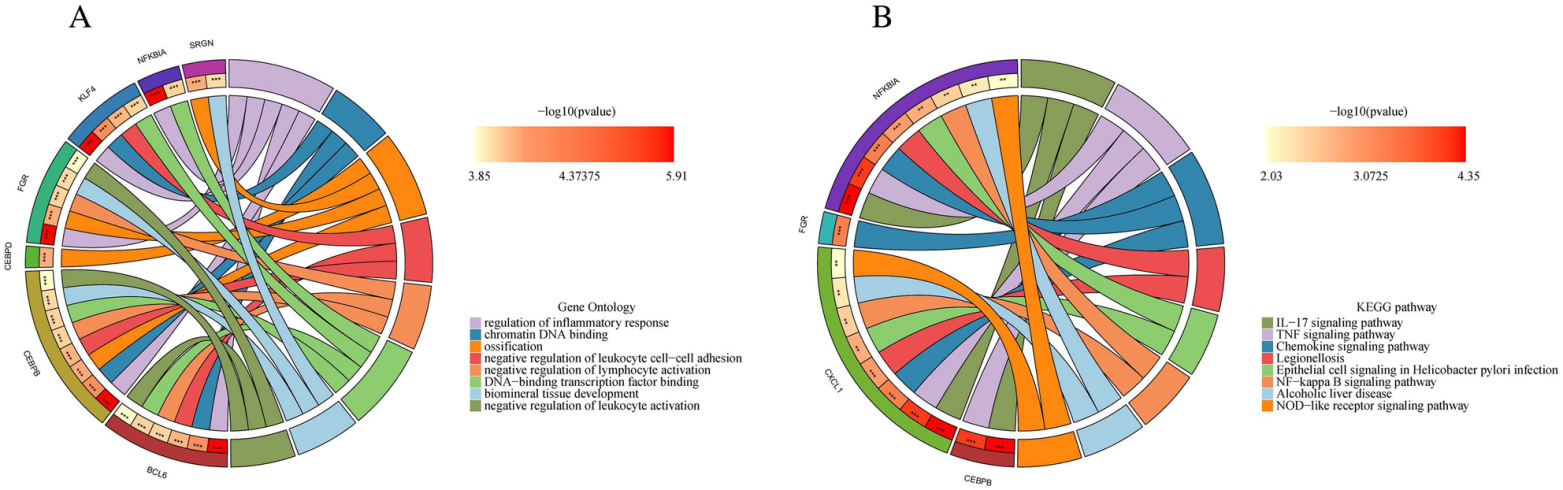
Hub gene functional enrichment analysis. (**A**) The circle plot via GO analysis. (**B**) The circle plot via GO analysis.

GO analysis showed that these hub genes are mainly involved in regulation of inflammatory response, chromatin DNA binding, and ossification (Fig. 5A) (Supplementary Table 6). In addition, KEGG Pathway analysis showed that they are mainly involved in the IL-17 signaling pathway, TNF signaling pathway, and Chemokine signaling pathway (Fig. 5B) (Supplementary Table 7). The above hub genes functional analysis shows that the AMI and the AD have a strong relationship with inflammation.

### Establishment and evaluation of the prognosis model

**Figure 6:**
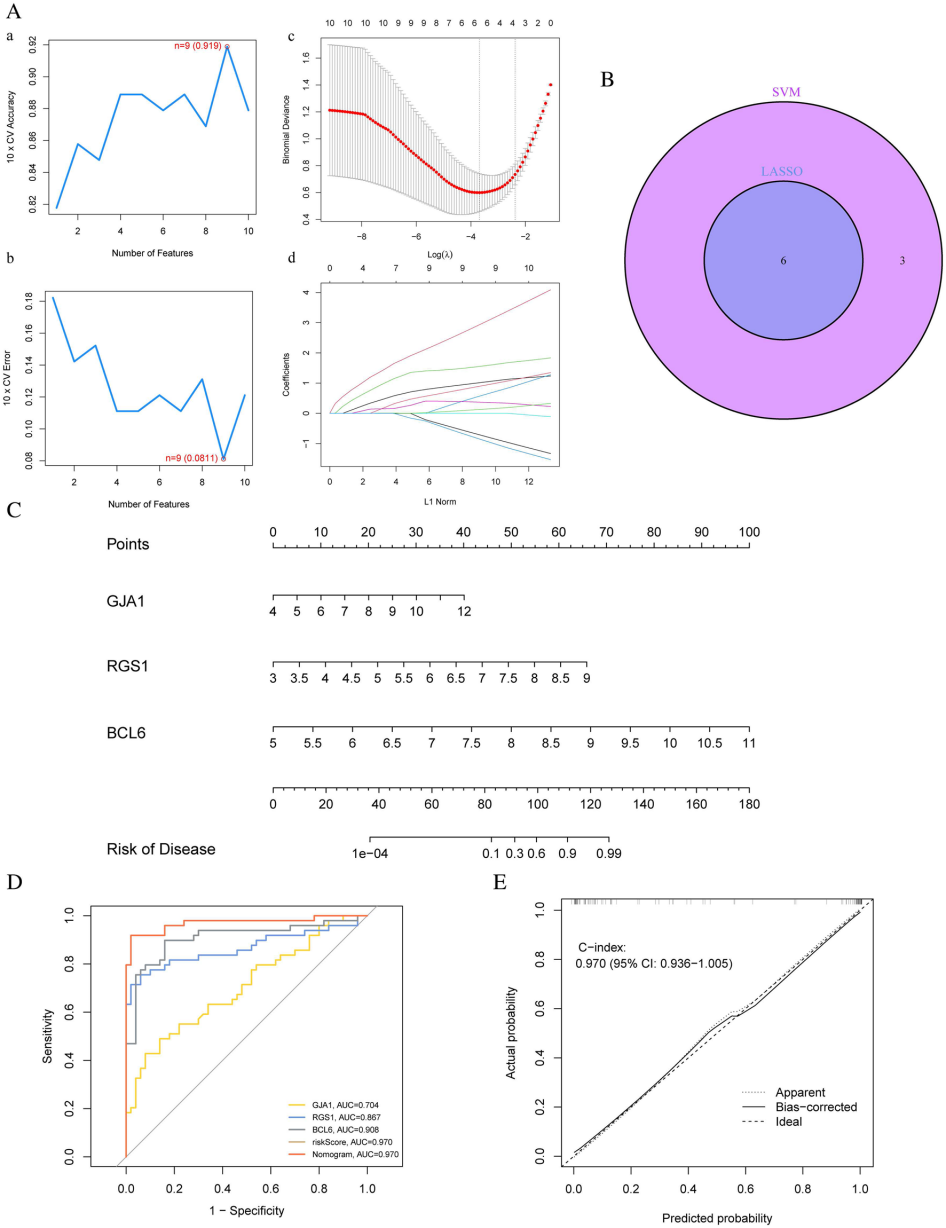
Establishment and Evaluation of the AMI Prognosis Model. (**A**) The SVM‐RFE and LASSO algorithm, (**B**) The six marker genes obtained from the LASSO and SVM-RFE models. (**C**) The nomogram of the Prognosis Model. (**D**) ROC analysis of this model. (**E**) The calibration curve.

We then applied the SVM‐RFE algorithm to filter the ten hub genes to identify the optimal combination of feature genes. Nine genes (maximal accuracy = 0.919, minimal errors = 0.0811) were identified as the optimal feature genes for the AMI patients (Fig. 6A a,b). Meanwhile, we used the LASSO method to construct a model for the target gene set, and based on the ten-fold cross-validation, we obtained a six gene model graph (Fig. 6A c,d). In addition, we took the intersection genes between the SVM-RFE and LASSO results to obtain six model genes (Fig. 6B). Next, we analyzed these six genes with logistic regression and found that only three hub genes with a p-value less than 0.05 were obtained: GAJ1, BCL6 and RGS1. Further, we constructed the predictive model using the two hub genes. To calculate the risk score of each one, the following formula was applied: Risk score = BCL6 × (2.5463) + RGS1 × (1.6769) + RGS1 × (0.7661). This model’s nomogram was also generated in Fig. 6C for visualization and clinical usage of the diagnostic model. Users can find three genes’ blood expression levels on the nomogram and project them to the top point scale to read each variant’s point. By projecting the total points downward, one might estimate the risk likelihood that this patient has AMI based on the bottom scale. In addition, we created a ROC curve to assess the AMI diagnostic model’s predictive accuracy. The training set’s AUC of the ROC curve was 0.970, indicating the model’s strong predictive capacity (Fig. 6D). The calibration curve of the training set was 0.970 when the nomogram was tested using the calibration curve, suggesting that the model has the ability to identify (Fig. 6E).

**Figure 7:**
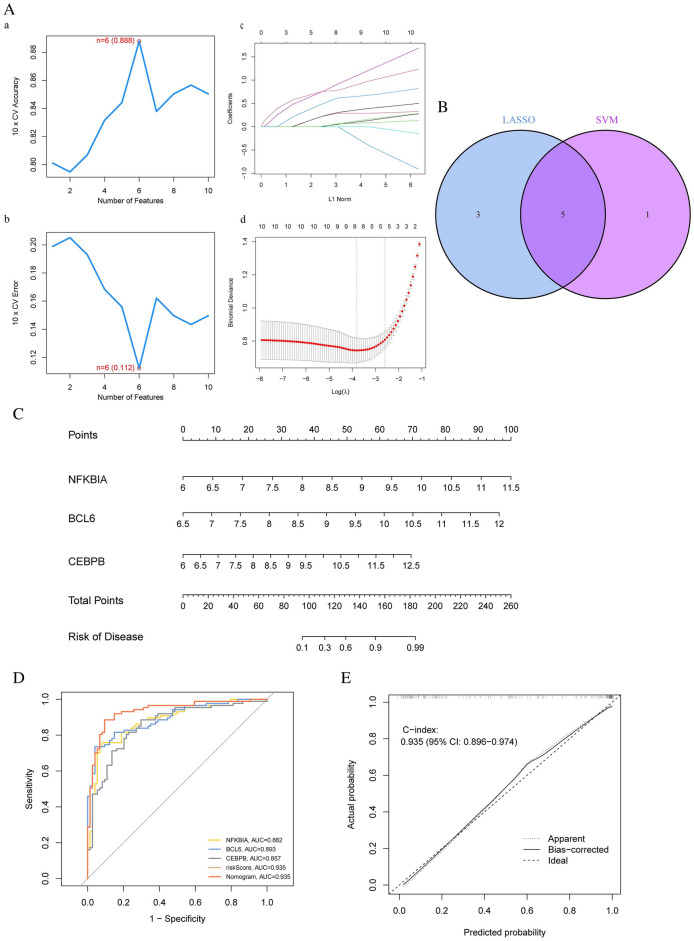
Establishment and Evaluation of the AD Prognosis Model. (**A**) The SVM‐RFE and LASSO algorithm, (**B**) The five marker genes obtained from the LASSO and SVM-RFE models. (**C**) The nomogram of the Prognosis Model. (**D**) ROC analysis of this model. (**E**) The calibration curve.

Meanwhile, six genes (maximal accuracy = 0.888, minimal errors = 0.112) were identified as the optimal feature genes for AD patients through the SVM-RFE algorithm (Fig. 7A a,b). Meanwhile, we used the LASSO method to construct a model for the target gene set, and based on the ten-fold cross-validation, we obtained an eight gene model graph (Fig. 7A c,d). In addition, we took the intersection genes between the SVM-RFE and LASSO results to obtain five model genes (Fig. 7B). Next, we analyzed these six genes with logistic regression and found that three hub genes with a p-value less than 0.05 were obtained: CEBPB, BCL6, and NFKBIA. Further, we constructed the predictive model using the three hub genes. To calculate the risk score of each one, the following formula was applied: Risk score = CEBPB × (0.8110) + BCL6 × (1.3287) + NFKBIA × (1.3785). This model’s nomogram was also generated in Fig. 7C for visualization and clinical usage of the diagnostic model. Moreover, we created a ROC curve to assess the AMI diagnostic model’s predictive accuracy. The training set’s AUC of the ROC curve was 0.935, indicating the model’s strong predictive capacity (Fig. 7D). The calibration curve of the training set was 0.935 when the nomogram was tested using the calibration curve, suggesting that the model has the ability to identify (Fig. 7E).

### GSVA analysis

**Figure 8:**
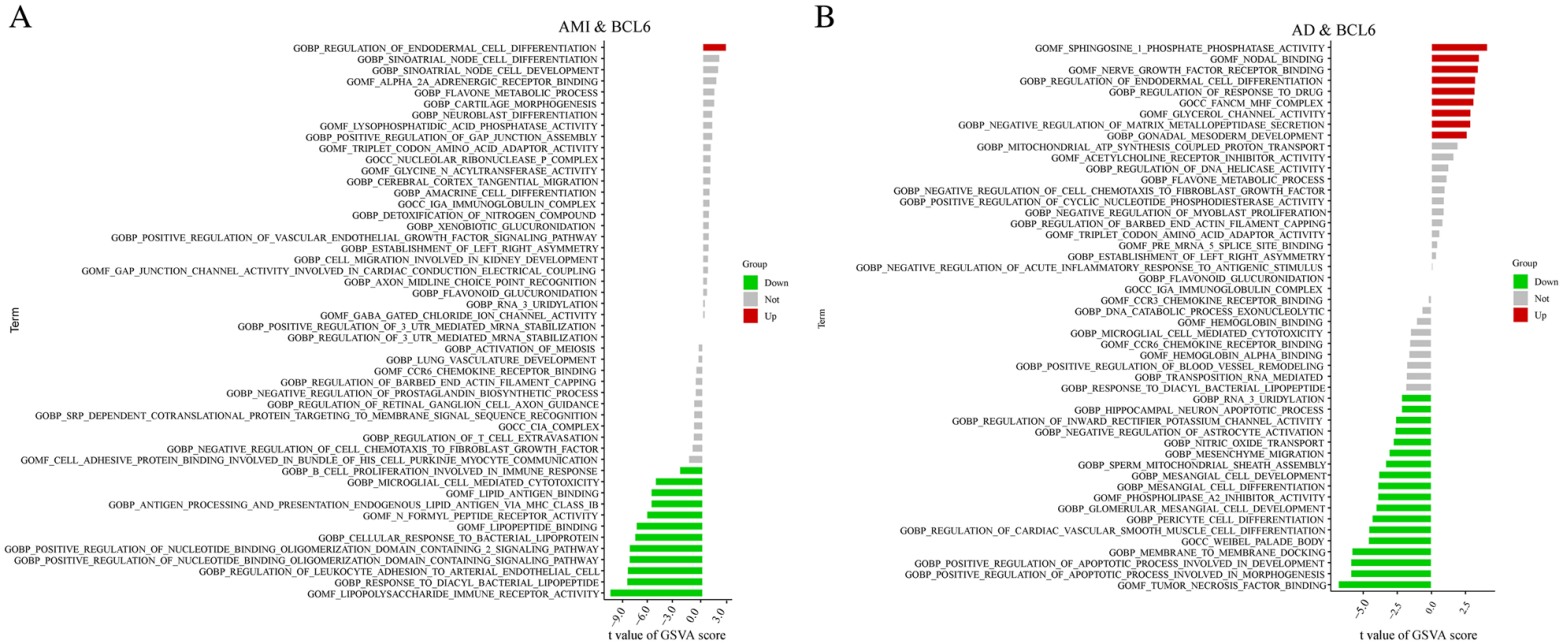
Gene set variation analysis. (**A**) GSVA between AMI and normal samples. (**b**) GSVA between AD and normal samples.

We intersected the model genes based on the AMI and AD prediction models and discovered that the BCL6 gene differed between the two disorders. Subsequently, we ran GSVA analysis on a BCL6-based training set. Based on GO functional analysis, we found that AMI individuals had a high expression about cell proliferation, such as regulation of endodermal cell-differentiation, while had a lower expression about immune response, such as lipopolysaccharide immune receptor activity (Fig. 8A). Besides, for AD patients, we found that AD individuals had a high expression about cell proliferation and migration, such as sphingosine-I-phosphate phosphatase activity, while had a lower expression about immune response, such as tumor necrosis factor binding (Fig. 8B).

### Construction of ceRNA network

**Figure 9:**
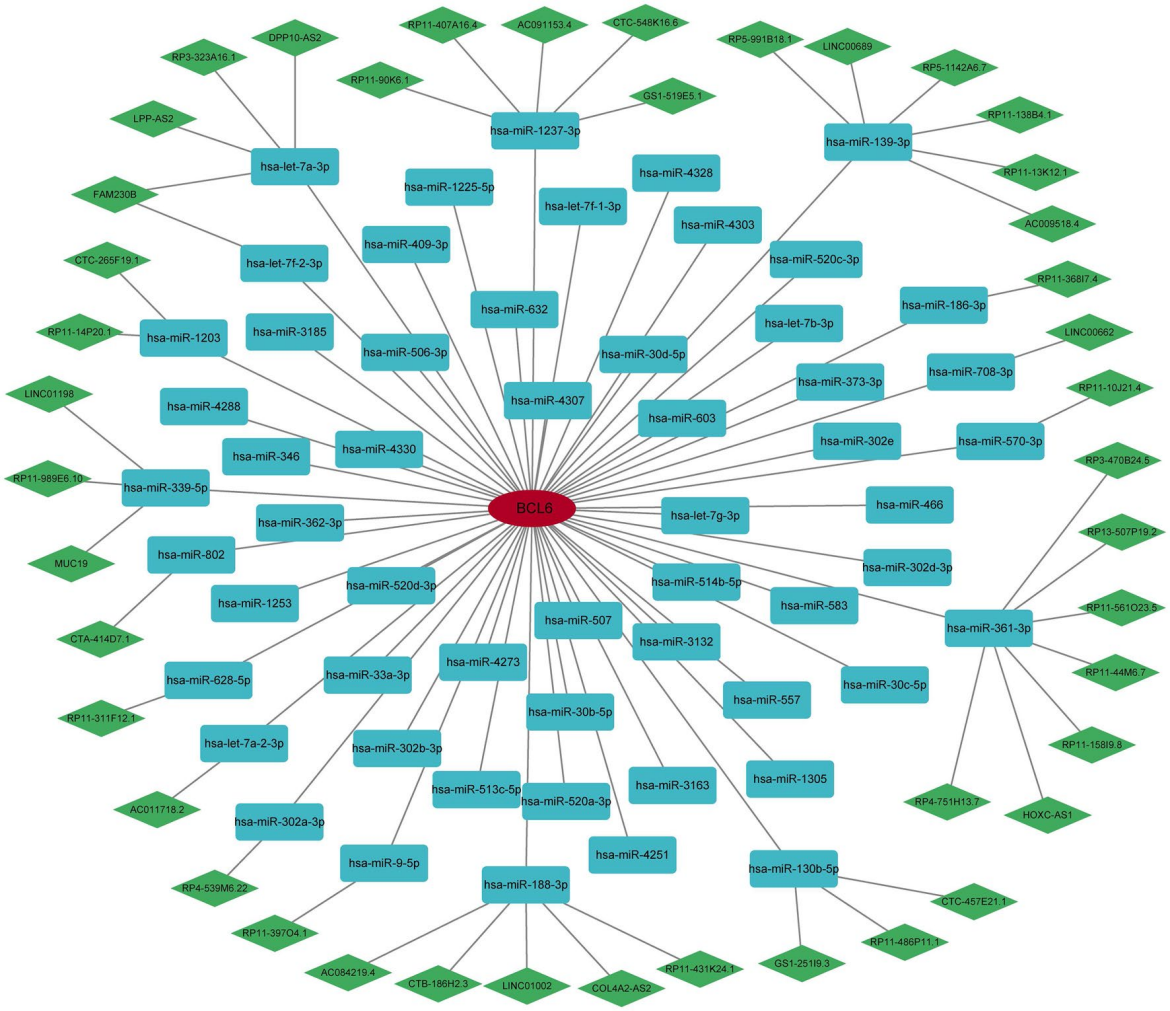
The ceRNA network of BCL6.

Next, we constructed a ceRNA network based on BCL6 through StarBase and Miranda databases. The network included 100 nodes (one marker gene, 56 miRNAs, and 43 lncRNAs) and 100 edges (Fig. 9). In detail, we found that only 17 out of 56 miRNAs were associated with 44 lncRNAs. Among them, hsa-miR-361-3p has the highest number of related lncRNAs, which are correlated with seven lncRNAs, including RP3-470B24.5, RP13-507P19.2, HOXC-AS1, RP11-158I9.8, RP11-44M6.7, RP11-561O23.5, and RP4-751H13.7. In addition, we found a correlation between FAM230B and two miRNAs, including hsa-let-7a-3p and hsa-let-7f-2-3p.

### Key gene validation

**Figure 10:**
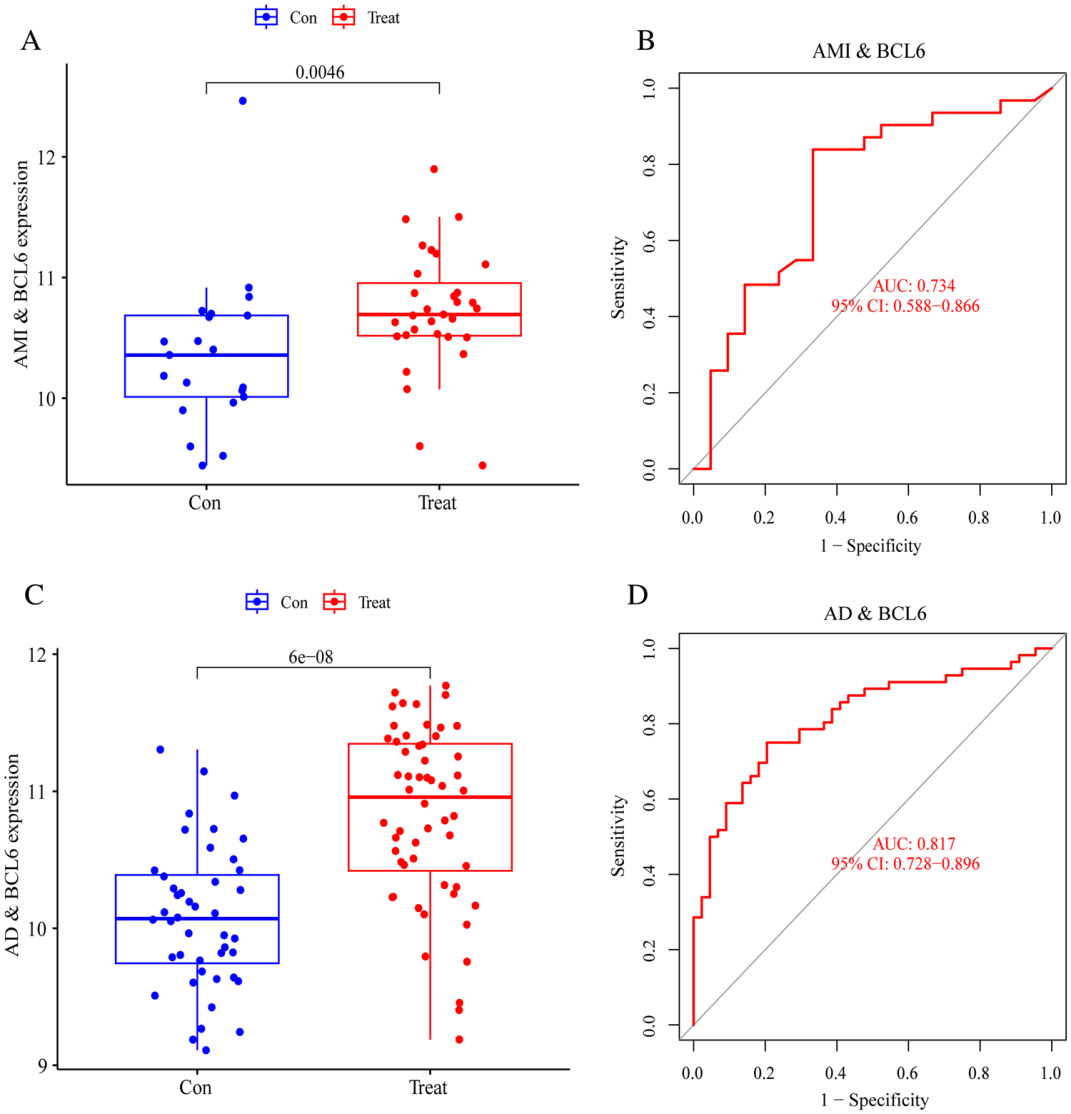
Key gene validation. (**A**,**B**) Validation in AMI. (**A**) BCL6 expression in GSE48060. (**B**) ROC analysis in GSE48060. (**C**,**D**) Validation in AD. (**C**) BCL6 expression in GSE122063. (**D**) ROC analysis in GSE122063.

Moreover, we corroborated that BCL6 was expressed in the validation dataset. The expression trends of BCL6 were found to be in agreement with the test dataset. We created ROC curves and determined the associated area under the curve (AUC) of these gene expression levels to validate the diagnostic value of BCL6 found in the investigation. The BCL6 gene was discovered to be expressed at a higher level in AMI patients than in normal patients, and the single gene prediction ability for AMI patients is strong (AUC = 0.734) (Fig. 10A,B). Additionally, AD patients’ BCL6 expression levels were still rising, and the single gene prediction performance is strong (AUC = 0.817) (Fig. 10C,D).

## Discussion

In modern Western countries, cardiovascular diseases were the leading causes of morbidity and mortality^27–29^. It is presently believed that pathogenetic pathways resulting in atherogenesis and atherosclerosis complications are abnormal lipid metabolism and inflammatory processes. For example, a slight increase in blood C reactive protein (CRP) levels, a measure of inflammation, is linked to an increased risk of cardiovascular events^30^. Promoter polymorphisms having functional significance in the expression of the cognate inflammatory gene are frequently observed in patients with AMI^31^.

AD was the most frequent type of dementia in the elderly^32^. Pathologically, it was distinguished by the loss of neuronal synapses, extracellular amyloid protein deposits known as neuritic plaques, and intracellular production of defective neurofilaments that form neurofibrillary tangles^33^. Previous studies had revealed that inflammatory processes have been detected in the brains of people with AD^34^. Besides, gene variations that increase inflammation or change cholesterol transport had been commonly discovered in AD patients^35,36^.

Despite these similarities, little data explicitly connected AD and AMI. Epidemiological studies that demonstrated the reduction of inflammation by the use of statins or non-steroidal anti-inflammatory medicines (NSAIDs) decreased the incidence of both AD and AMI might provide the most substantial evidence yet that AMI and AD are related^28,37^. Nevertheless, no effort had been undertaken to assess the possibility that particular immunological genetic risk factors represent a significant pathogenetic and etiologic connection between AMI and AD. Therefore, it was essential to investigate the molecular mechanisms behind these two diseases and identify targets to stop the early disease’s progression.

We discovered 22 shared DEGs between AD and AMI in this study by exploring the GEO databases for these two disorders. In addition, we carried out analyses of the KEGG pathway and GO enrichment, and we built a PPI network to determine which ten hub genes were among the DEGs. Next, we used the SVM-RFE and multivariable logistic regression analysis technique to build models for patients with AMI and AD. The BCL6 gene exhibited differential expression in both illnesses and demonstrated a strong predictive capacity (*p* < 0.05, AUC > 0.8). This means we must accept the genetic resemblance between AMI and AD.

While vigorous acute inflammatory responses were necessary for survival, uncontrolled inflammation was at the root of many diseases, including atherosclerosis. Chronic inflammation was a hallmark of atherosclerosis to the extent that acute transcriptional induction is a driver of inflammation. From early fatty streaks to mature lesions, MØ played a critical role in atherogenesis while the BCL6 played a pivotal role in MØ quiescence and the regulation of inflammation^38^. Previous research had found that BCL6 reduces oxidized LDL and TLR-induced inflammation and atherogenesis, indicating that BCL6 plays an important role in reducing early vascular lesion development^39^. Surprisingly, nevertheless, our research revealed that BCL6 gene expression was up-regulated, which may be connected to negative feedback regulation in individuals with high levels of atherosclerosis. Interestingly, prior research had also discovered increased BCL6 gene expression in individuals suffering from coronary heart disease^40^. Hence, additional mechanisms must be proven through additional tests.

Previous data unraveled that bone marrow mesenchymal stem cells-derived extracellular vesicles carrying miR-302d-3p repressed cardiomyocyte injury and inflammatory response after AMI by disrupting the NF-κB pathway via the BCL6/MD2 axis, which means the BCL6 played an essential role in early inflammation response^41^. Concurrent with this, a prior work discovered that BCL6 upregulation participated in the promotion of oxygen–glucose deprivation-induced increase of apoptosis and decreased cell proliferation after ischemic stroke^42^. Also, BCL6 upregulation was able to aggravate the pulmonary inflammation following influenza virus infection^43^. These data indirectly supported the effect of BCL6 on apoptosis, inflammation, and viability.

Although prior studies individually investigated the hub genes related to AMI and AD, few studies have used bioinformatics approaches to investigate their common molecular mechanism^44,45^. Due to the significant comorbidity rate between AMI and AD, we found the common DEGs and hub genes for the first time, which helped clarify the process. Nonetheless, this investigation is not without its drawbacks. The current study was based on a thorough bioinformatics analysis; even though we used an external dataset for additional validation, more research is still required to demonstrate the relationship between feature genes and AMI pathophysiology, which will be the focus of our future work.

## Conclusion

In conclusion, we carried out enrichment and PPI network analysis, as well as determined the shared DEGs of AMI and AD. Our research revealed that AMI and AD shared numerous pathogenic pathways, maybe mediated by certain hub genes. In the development of AMI and AD, the hub gene, specifically the BCL6, is crucial. This study adds to our understanding of the molecular mechanism of AMI in combination with AD.

### Supplementary Information

Supplementary Table 1.

Supplementary Table 2.

Supplementary Table 3.

Supplementary Table 4.

Supplementary Table 5.

Supplementary Table 6.

Supplementary Table 7.

## Data Availability

The datasets analyzed in this work may be found in the GEO database. All software applications used are included in this article.
