# Supplementary material for: Exploring the pathogenesis and key genes associated of acute myocardial infarction complicated with Alzheimer’s disease
Source: Sci Rep. 2024 Jan 16;14:1449. doi: 10.1038/s41598-024-52094-4 (PMC10791667; doi:10.1038/s41598-024-52094-4)
Supplement: Supplementary file 1 — Supplementary Table 1. [file 41598_2024_52094_MOESM1_ESM.docx]

| id | logFC | AveExpr | t | P.Value | adj.P.Val | B |
| --- | --- | --- | --- | --- | --- | --- |
| GAS2L1 | 1.154658 | 9.421076 | 12.79745 | 1.92E-26 | 4.16E-22 | 49.37861 |
| DTNA | 1.548579 | 9.620347 | 12.40845 | 2.35E-25 | 2.55E-21 | 46.9262 |
| MALAT1 | 1.520338 | 12.62628 | 12.3013 | 4.69E-25 | 3.39E-21 | 46.25069 |
| PXDC1 | 1.547516 | 7.597953 | 12.12089 | 1.50E-24 | 8.12E-21 | 45.11372 |
| HSD17B7 | 1.173846 | 8.962614 | 11.96238 | 4.16E-24 | 1.71E-20 | 44.1152 |
| NSUN6 | 1.677921 | 8.616093 | 11.94227 | 4.73E-24 | 1.71E-20 | 43.98858 |
| AMER2 | 1.31055 | 11.22781 | 11.68863 | 2.41E-23 | 6.53E-20 | 42.39243 |
| SEMA4C | 1.263633 | 9.524193 | 11.59201 | 4.49E-23 | 1.08E-19 | 41.78503 |
| COL27A1 | 1.24065 | 6.809786 | 11.5601 | 5.51E-23 | 1.19E-19 | 41.58452 |
| ITPKB | 1.601648 | 6.931368 | 11.47715 | 9.38E-23 | 1.56E-19 | 41.0635 |
| NUCKS1 | 1.110271 | 11.69963 | 11.44737 | 1.13E-22 | 1.74E-19 | 40.87657 |
| ERBB2IP | 1.485113 | 8.733598 | 11.43789 | 1.21E-22 | 1.74E-19 | 40.81707 |
| SRRM2 | 1.800667 | 9.880708 | 11.32951 | 2.41E-22 | 3.27E-19 | 40.13709 |
| MAFF | 2.068099 | 8.347027 | 11.29754 | 2.96E-22 | 3.77E-19 | 39.9366 |
| KLF15 | 1.519319 | 8.042212 | 11.16996 | 6.70E-22 | 8.06E-19 | 39.1373 |
| ARHGEF40 | 1.205566 | 7.042526 | 11.15497 | 7.38E-22 | 8.41E-19 | 39.04349 |
| BCL6 | 1.349041 | 9.406777 | 11.14404 | 7.91E-22 | 8.56E-19 | 38.97504 |
| MED13L | 1.070452 | 8.087288 | 11.1165 | 9.43E-22 | 9.73E-19 | 38.80267 |
| SASH1 | 1.359061 | 9.963719 | 11.09236 | 1.10E-21 | 9.93E-19 | 38.65168 |
| TNPO1 | 1.165725 | 10.06009 | 11.07827 | 1.20E-21 | 1.01E-18 | 38.5635 |
| MAP4K4 | 1.257762 | 9.114471 | 11.07778 | 1.21E-21 | 1.01E-18 | 38.56049 |
| CREBBP | 1.035976 | 8.400212 | 11.019 | 1.76E-21 | 1.36E-18 | 38.19292 |
| SLC35E1 | 1.354985 | 9.428979 | 10.99989 | 1.99E-21 | 1.48E-18 | 38.07351 |
| JUND | 1.242084 | 9.73937 | 10.99342 | 2.07E-21 | 1.49E-18 | 38.03311 |
| IDH3G | -1.33873 | 7.501625 | -10.8969 | 3.83E-21 | 2.67E-18 | 37.43023 |
| NFIA | 1.40882 | 8.95919 | 10.86496 | 4.69E-21 | 3.11E-18 | 37.2311 |
| ANP32B | 1.436282 | 10.32056 | 10.83858 | 5.55E-21 | 3.49E-18 | 37.06655 |
| ATP5C1 | -1.40217 | 9.818193 | -10.8359 | 5.65E-21 | 3.49E-18 | 37.05009 |
| FAM63A | 1.652003 | 8.746348 | 10.79403 | 7.37E-21 | 4.43E-18 | 36.78884 |
| TUBB | -1.12574 | 9.215083 | -10.7559 | 9.39E-21 | 5.35E-18 | 36.55155 |
| ACACB | 1.597033 | 8.215844 | 10.75096 | 9.69E-21 | 5.38E-18 | 36.5205 |
| MKNK2 | 1.581703 | 10.14539 | 10.70754 | 1.28E-20 | 6.76E-18 | 36.25017 |
| BBX | 1.069347 | 8.460621 | 10.70685 | 1.28E-20 | 6.76E-18 | 36.24591 |
| NFKBIA | 1.280058 | 8.561127 | 10.70348 | 1.31E-20 | 6.76E-18 | 36.2249 |
| TOB1 | 1.396782 | 9.730894 | 10.65403 | 1.79E-20 | 9.01E-18 | 35.91727 |
| PFKFB3 | 1.355275 | 11.47911 | 10.65081 | 1.83E-20 | 9.01E-18 | 35.89723 |
| SNRNP48 | 1.04332 | 7.525149 | 10.64265 | 1.93E-20 | 9.28E-18 | 35.8465 |
| CTD-3092A11.2 | 1.548888 | 8.508547 | 10.61746 | 2.26E-20 | 1.05E-17 | 35.6899 |
| KIAA0485 | 1.828923 | 7.782416 | 10.5358 | 3.80E-20 | 1.68E-17 | 35.18271 |
| LUC7L3 | 1.014104 | 11.30217 | 10.49232 | 5.00E-20 | 2.11E-17 | 34.91299 |
| QKI | 1.294152 | 9.86017 | 10.48486 | 5.25E-20 | 2.14E-17 | 34.86668 |
| ADAM33 | 1.573737 | 7.801506 | 10.45218 | 6.45E-20 | 2.51E-17 | 34.66409 |
| RFX4 | 1.442325 | 7.299361 | 10.45091 | 6.50E-20 | 2.51E-17 | 34.65621 |
| EZR | 1.616487 | 6.766148 | 10.43406 | 7.24E-20 | 2.75E-17 | 34.55184 |
| TAF3 | 1.077891 | 7.810151 | 10.37471 | 1.05E-19 | 3.93E-17 | 34.18427 |
| DNAJC1 | 1.197304 | 9.133125 | 10.37061 | 1.08E-19 | 3.97E-17 | 34.15893 |
| CDK13 | 1.191877 | 8.968097 | 10.30307 | 1.66E-19 | 5.78E-17 | 33.74121 |
| LOC101929787 | 1.639716 | 8.32675 | 10.29826 | 1.71E-19 | 5.87E-17 | 33.71147 |
| SOX2 | 1.211907 | 8.543373 | 10.26384 | 2.12E-19 | 6.96E-17 | 33.49881 |
| ATP8B1 | 1.483731 | 8.927533 | 10.21691 | 2.85E-19 | 9.21E-17 | 33.20911 |
| NOTCH2 | 1.488436 | 8.36287 | 10.21438 | 2.90E-19 | 9.22E-17 | 33.1935 |
| LPP | 1.113969 | 8.003306 | 10.1678 | 3.88E-19 | 1.22E-16 | 32.9063 |
| NOTCH2NL | 1.686178 | 10.80588 | 10.1333 | 4.83E-19 | 1.45E-16 | 32.69366 |
| HIPK2 | 1.504903 | 9.256457 | 10.11912 | 5.28E-19 | 1.56E-16 | 32.60636 |
| MSI2 | 1.072179 | 7.54031 | 10.11145 | 5.54E-19 | 1.62E-16 | 32.55915 |
| VCAN | 1.291022 | 8.146784 | 10.10315 | 5.83E-19 | 1.68E-16 | 32.50803 |
| PRR34-AS1 | 1.570274 | 8.953919 | 10.09223 | 6.25E-19 | 1.78E-16 | 32.44081 |
| GLUL | 1.393666 | 11.73445 | 10.04967 | 8.16E-19 | 2.24E-16 | 32.17906 |
| PGF | 1.638908 | 10.11738 | 10.02663 | 9.43E-19 | 2.55E-16 | 32.03741 |
| RP11-403P17.4 | 1.578405 | 10.72588 | 10.02083 | 9.78E-19 | 2.61E-16 | 32.00183 |
| PRR11 | 1.536176 | 10.75138 | 10.00396 | 1.09E-18 | 2.87E-16 | 31.89817 |
| KIF5B | 1.192954 | 9.981749 | 10.001 | 1.11E-18 | 2.89E-16 | 31.88001 |
| PLSCR4 | 1.589388 | 8.95243 | 9.983958 | 1.23E-18 | 3.15E-16 | 31.77535 |
| ZFP36L1 | 1.605706 | 7.234201 | 9.983668 | 1.23E-18 | 3.15E-16 | 31.77357 |
| HIP1R | 1.128793 | 8.336047 | 9.936513 | 1.66E-18 | 4.13E-16 | 31.48425 |
| ZC3H7B | 1.22295 | 7.646532 | 9.928303 | 1.75E-18 | 4.25E-16 | 31.43391 |
| ANKRD36 | 1.922596 | 8.967329 | 9.91256 | 1.93E-18 | 4.64E-16 | 31.33741 |
| RP11-194N12.2 | 1.814717 | 6.202769 | 9.893413 | 2.17E-18 | 5.17E-16 | 31.22009 |
| KMT2E | 1.019032 | 9.590411 | 9.871562 | 2.49E-18 | 5.80E-16 | 31.08628 |
| ATP5B | -1.20648 | 10.55019 | -9.8636 | 2.62E-18 | 6.03E-16 | 31.03752 |
| LIFR | 1.403677 | 8.34702 | 9.853821 | 2.78E-18 | 6.28E-16 | 30.97768 |
| PSMB3 | -1.12654 | 8.684114 | -9.82051 | 3.43E-18 | 7.57E-16 | 30.77391 |
| RAB11FIP3 | 1.419828 | 9.05697 | 9.817654 | 3.49E-18 | 7.63E-16 | 30.75644 |
| PCYOX1L | -1.3166 | 7.847003 | -9.80847 | 3.70E-18 | 8.00E-16 | 30.70032 |
| ME3 | -1.02776 | 7.308265 | -9.76046 | 4.99E-18 | 1.05E-15 | 30.40698 |
| RPS16P5 | 1.769098 | 7.312682 | 9.755659 | 5.14E-18 | 1.07E-15 | 30.37768 |
| LOC202181 | 2.341414 | 8.168296 | 9.728154 | 6.10E-18 | 1.26E-15 | 30.20984 |
| PTMA | 1.119705 | 10.50356 | 9.703527 | 7.11E-18 | 1.45E-15 | 30.05966 |
| LRP4 | 1.718586 | 9.615346 | 9.636137 | 1.08E-17 | 2.11E-15 | 29.6492 |
| RAD51C | -1.31081 | 6.680504 | -9.63065 | 1.12E-17 | 2.15E-15 | 29.6158 |
| PALLD | 1.117744 | 8.04256 | 9.630251 | 1.12E-17 | 2.15E-15 | 29.61338 |
| MT1M | 1.593954 | 8.063378 | 9.616947 | 1.22E-17 | 2.29E-15 | 29.53245 |
| MNT | 1.192649 | 9.921863 | 9.614994 | 1.23E-17 | 2.30E-15 | 29.52057 |
| ZBED6 | 1.299469 | 6.312425 | 9.584994 | 1.49E-17 | 2.70E-15 | 29.3382 |
| AFF1 | 1.122485 | 8.432157 | 9.577812 | 1.55E-17 | 2.80E-15 | 29.29456 |
| RAP1GDS1 | -1.00233 | 8.803638 | -9.57676 | 1.56E-17 | 2.80E-15 | 29.28815 |
| IQCA1 | 1.177226 | 7.560573 | 9.575114 | 1.58E-17 | 2.80E-15 | 29.27817 |
| NME1 | -1.39631 | 8.978977 | -9.5643 | 1.69E-17 | 2.97E-15 | 29.2125 |
| CASP6 | 1.073474 | 6.171184 | 9.51248 | 2.33E-17 | 3.97E-15 | 28.89802 |
| CCDC152 | 1.595305 | 10.06792 | 9.511146 | 2.35E-17 | 3.97E-15 | 28.88993 |
| ID4 | 1.389237 | 8.452033 | 9.508626 | 2.39E-17 | 4.00E-15 | 28.87465 |
| UBXN2A | 1.16464 | 9.711376 | 9.475827 | 2.92E-17 | 4.79E-15 | 28.67588 |
| ITGB5 | 1.217061 | 7.677096 | 9.456139 | 3.30E-17 | 5.33E-15 | 28.55665 |
| COPG2IT1 | -1.27483 | 11.05982 | -9.44897 | 3.45E-17 | 5.53E-15 | 28.51327 |
| NLN | 1.211098 | 9.189626 | 9.430982 | 3.86E-17 | 6.14E-15 | 28.40441 |
| TP53INP1 | 1.212794 | 6.259724 | 9.417161 | 4.20E-17 | 6.59E-15 | 28.32082 |
| MEGF10 | 1.295597 | 9.054894 | 9.359262 | 6.01E-17 | 9.09E-15 | 27.97103 |
| GFAP | 1.945986 | 11.2933 | 9.344617 | 6.57E-17 | 9.81E-15 | 27.88265 |
| RBM25 | 1.141197 | 10.01431 | 9.303777 | 8.45E-17 | 1.25E-14 | 27.63641 |
| EMC4 | -1.00133 | 8.641383 | -9.30327 | 8.48E-17 | 1.25E-14 | 27.63335 |
| CMBL | 1.334376 | 10.30181 | 9.298352 | 8.74E-17 | 1.28E-14 | 27.60373 |
| NDUFA7 | -1.58531 | 6.350337 | -9.26179 | 1.09E-16 | 1.58E-14 | 27.38361 |
| ANKDD1A | 1.068851 | 8.552183 | 9.251252 | 1.17E-16 | 1.67E-14 | 27.32019 |
| DDIT4 | 1.591184 | 9.11602 | 9.242881 | 1.23E-16 | 1.75E-14 | 27.26985 |
| NACC2 | 1.360723 | 9.364538 | 9.240979 | 1.24E-16 | 1.76E-14 | 27.25841 |
| PMP2 | 1.555139 | 10.31592 | 9.236204 | 1.28E-16 | 1.79E-14 | 27.2297 |
| AP3M2 | -1.05534 | 8.970239 | -9.2268 | 1.36E-16 | 1.88E-14 | 27.17316 |
| MXI1 | 1.220276 | 10.49023 | 9.189722 | 1.70E-16 | 2.29E-14 | 26.95045 |
| SYMPK | 1.086718 | 7.210637 | 9.164648 | 1.99E-16 | 2.66E-14 | 26.8 |
| LOC100272216 | 1.61209 | 8.917784 | 9.161482 | 2.03E-16 | 2.68E-14 | 26.78101 |
| HIF3A | 1.080196 | 5.67933 | 9.15996 | 2.05E-16 | 2.68E-14 | 26.77188 |
| CALM1 | -1.91342 | 7.216204 | -9.14712 | 2.21E-16 | 2.89E-14 | 26.69489 |
| ESF1 | 1.050012 | 8.794602 | 9.13848 | 2.33E-16 | 3.01E-14 | 26.64312 |
| PSMB7 | -1.05084 | 8.471208 | -9.13831 | 2.34E-16 | 3.01E-14 | 26.64209 |
| TGFBR3 | 1.238377 | 8.556834 | 9.136293 | 2.36E-16 | 3.03E-14 | 26.63001 |
| ZNF423 | 1.400803 | 7.932595 | 9.131911 | 2.43E-16 | 3.08E-14 | 26.60376 |
| STK35 | 1.093076 | 9.668977 | 9.116693 | 2.67E-16 | 3.35E-14 | 26.51261 |
| TUBB4B | -1.26366 | 10.47525 | -9.11602 | 2.68E-16 | 3.35E-14 | 26.50861 |
| ANXA6 | -1.26214 | 7.113408 | -9.11513 | 2.69E-16 | 3.35E-14 | 26.50326 |
| ATP6V1G2 | -1.41614 | 9.374561 | -9.10939 | 2.79E-16 | 3.45E-14 | 26.46885 |
| PTPN3 | -1.40831 | 6.133386 | -9.10114 | 2.93E-16 | 3.61E-14 | 26.41949 |
| C9orf64 | 1.286776 | 9.814919 | 9.075543 | 3.43E-16 | 4.15E-14 | 26.26638 |
| PCDHGA4 | 1.174426 | 7.612491 | 9.073654 | 3.47E-16 | 4.17E-14 | 26.25508 |
| PALD1 | 1.457506 | 6.977325 | 9.064371 | 3.67E-16 | 4.39E-14 | 26.19959 |
| MYO10 | 1.259406 | 8.018854 | 9.053918 | 3.91E-16 | 4.58E-14 | 26.13713 |
| MLLT11 | -1.18879 | 12.44398 | -9.0482 | 4.05E-16 | 4.72E-14 | 26.10299 |
| ZNF721 | 1.16404 | 10.84122 | 9.022076 | 4.75E-16 | 5.44E-14 | 25.94698 |
| TJAP1 | 1.052347 | 8.051579 | 9.020216 | 4.81E-16 | 5.48E-14 | 25.93589 |
| ZNF160 | 1.363512 | 9.195691 | 9.0098 | 5.12E-16 | 5.81E-14 | 25.87374 |
| ZNRF3 | 1.265435 | 9.133228 | 9.007585 | 5.19E-16 | 5.85E-14 | 25.86052 |
| DDX59 | 1.180686 | 8.649996 | 9.006161 | 5.24E-16 | 5.87E-14 | 25.85203 |
| CEBPB | 1.25621 | 9.377505 | 8.993024 | 5.67E-16 | 6.33E-14 | 25.77369 |
| ITPRIPL2 | 1.005735 | 6.865594 | 8.976871 | 6.26E-16 | 6.92E-14 | 25.67742 |
| FBXW12 | 1.303741 | 9.726907 | 8.970014 | 6.53E-16 | 7.17E-14 | 25.63657 |
| TUBB3 | -1.3305 | 10.08403 | -8.96425 | 6.76E-16 | 7.36E-14 | 25.60225 |
| SLC35B1 | -1.16851 | 7.459233 | -8.95031 | 7.36E-16 | 7.93E-14 | 25.51927 |
| HDAC7 | 1.258897 | 5.61924 | 8.926362 | 8.51E-16 | 8.95E-14 | 25.37676 |
| FAT1 | 1.412417 | 9.319861 | 8.919895 | 8.85E-16 | 9.20E-14 | 25.33831 |
| TJP1 | 1.088964 | 10.30593 | 8.902521 | 9.84E-16 | 1.00E-13 | 25.23505 |
| SLC25A4 | -1.06586 | 8.157152 | -8.90166 | 9.89E-16 | 1.00E-13 | 25.22991 |
| MT2A | 1.435572 | 11.46493 | 8.858867 | 1.28E-15 | 1.28E-13 | 24.97589 |
| SKI | 1.033695 | 7.945678 | 8.858106 | 1.29E-15 | 1.28E-13 | 24.97137 |
| SAMD4A | 1.138839 | 7.832774 | 8.839594 | 1.44E-15 | 1.42E-13 | 24.86161 |
| PDCD6 | 1.005211 | 9.319798 | 8.839467 | 1.44E-15 | 1.42E-13 | 24.86086 |
| ADCYAP1 | -1.80955 | 5.593898 | -8.83847 | 1.45E-15 | 1.42E-13 | 24.85495 |
| TBL1X | 1.131447 | 7.14592 | 8.829985 | 1.53E-15 | 1.48E-13 | 24.80467 |
| UCHL1 | -1.49789 | 9.892124 | -8.78043 | 2.06E-15 | 1.95E-13 | 24.51136 |
| CSRNP1 | 1.226689 | 7.500625 | 8.776814 | 2.11E-15 | 1.98E-13 | 24.48995 |
| TNS1 | 1.306849 | 8.128762 | 8.772938 | 2.16E-15 | 2.01E-13 | 24.46704 |
| RELL1 | 1.328142 | 8.268451 | 8.769274 | 2.21E-15 | 2.05E-13 | 24.44538 |
| COL5A3 | 1.197684 | 7.464226 | 8.741651 | 2.61E-15 | 2.40E-13 | 24.28219 |
| AGAP4 | 1.423523 | 6.832778 | 8.7411 | 2.62E-15 | 2.40E-13 | 24.27894 |
| PSD2 | 1.167734 | 9.903742 | 8.721357 | 2.95E-15 | 2.68E-13 | 24.16243 |
| NEK7 | 1.255775 | 8.784207 | 8.712568 | 3.11E-15 | 2.80E-13 | 24.11059 |
| YAP1 | 1.424865 | 7.257789 | 8.711627 | 3.12E-15 | 2.81E-13 | 24.10504 |
| SOX9 | 1.370861 | 9.206604 | 8.707038 | 3.21E-15 | 2.87E-13 | 24.07798 |
| GLS2 | -1.18992 | 6.456877 | -8.68965 | 3.57E-15 | 3.15E-13 | 23.97547 |
| CSPG5 | 1.047892 | 10.5924 | 8.678252 | 3.82E-15 | 3.34E-13 | 23.90836 |
| GRAMD1C | 1.462748 | 8.543077 | 8.668871 | 4.04E-15 | 3.49E-13 | 23.85312 |
| ATP1A2 | 1.372349 | 11.74009 | 8.66666 | 4.10E-15 | 3.52E-13 | 23.84011 |
| BAG3 | 1.489106 | 8.791871 | 8.665305 | 4.13E-15 | 3.53E-13 | 23.83214 |
| CDK2AP1 | 1.214016 | 11.48016 | 8.660572 | 4.25E-15 | 3.61E-13 | 23.80428 |
| EMX2OS | 1.00137 | 8.099459 | 8.654459 | 4.41E-15 | 3.71E-13 | 23.76832 |
| INA | -1.48398 | 10.53267 | -8.65362 | 4.43E-15 | 3.72E-13 | 23.76339 |
| ACOT7 | -1.1935 | 8.607404 | -8.65167 | 4.48E-15 | 3.75E-13 | 23.75191 |
| SEMA3F | 1.386056 | 7.288892 | 8.636195 | 4.92E-15 | 4.10E-13 | 23.6609 |
| PHF6 | 1.111805 | 8.836614 | 8.630464 | 5.09E-15 | 4.23E-13 | 23.62722 |
| FAM107B | 1.519064 | 8.613771 | 8.618045 | 5.49E-15 | 4.45E-13 | 23.55425 |
| ZNF415 | -1.00545 | 7.641127 | -8.60087 | 6.08E-15 | 4.92E-13 | 23.45341 |
| SLC12A7 | 1.264 | 8.341752 | 8.595813 | 6.27E-15 | 5.03E-13 | 23.42372 |
| DBT | 1.102815 | 9.194714 | 8.561671 | 7.70E-15 | 6.02E-13 | 23.22351 |
| ANKRD13D | 1.128472 | 7.79116 | 8.559909 | 7.78E-15 | 6.06E-13 | 23.21319 |
| PARD3 | 1.048966 | 7.506663 | 8.548498 | 8.33E-15 | 6.42E-13 | 23.14634 |
| KCNJ10 | 1.398835 | 8.219464 | 8.521781 | 9.77E-15 | 7.41E-13 | 22.98997 |
| KCNE4 | 1.306767 | 5.688739 | 8.510359 | 1.05E-14 | 7.90E-13 | 22.92317 |
| MAPKBP1 | 1.062103 | 8.702873 | 8.50862 | 1.06E-14 | 7.95E-13 | 22.913 |
| FBXL17 | 1.031017 | 8.705022 | 8.484657 | 1.22E-14 | 9.05E-13 | 22.77299 |
| SST | -2.50551 | 7.082883 | -8.47939 | 1.26E-14 | 9.31E-13 | 22.74224 |
| SCARNA17 | 1.385506 | 6.163823 | 8.464215 | 1.38E-14 | 1.01E-12 | 22.65366 |
| GLIS3 | 1.175928 | 6.951639 | 8.455103 | 1.46E-14 | 1.06E-12 | 22.60051 |
| POU3F3 | 1.038746 | 8.405392 | 8.453235 | 1.47E-14 | 1.07E-12 | 22.58962 |
| CXCR4 | 1.418681 | 6.3302 | 8.440097 | 1.59E-14 | 1.15E-12 | 22.51303 |
| MGC12488 | 1.604892 | 7.834002 | 8.439354 | 1.60E-14 | 1.15E-12 | 22.50869 |
| ASAH2B | -1.08466 | 6.342363 | -8.41662 | 1.83E-14 | 1.31E-12 | 22.37626 |
| SLC5A3 | 1.002399 | 9.059089 | 8.412819 | 1.87E-14 | 1.33E-12 | 22.35414 |
| PLOD2 | 1.12838 | 8.058189 | 8.404199 | 1.97E-14 | 1.39E-12 | 22.30398 |
| ZCCHC24 | 1.41391 | 9.582699 | 8.349519 | 2.73E-14 | 1.89E-12 | 21.98623 |
| CD200 | -1.30604 | 8.077986 | -8.3393 | 2.90E-14 | 2.00E-12 | 21.92692 |
| RASEF | 1.469622 | 10.32049 | 8.335556 | 2.97E-14 | 2.03E-12 | 21.90522 |
| LOC100289333 | 1.153562 | 10.11741 | 8.313638 | 3.38E-14 | 2.28E-12 | 21.77816 |
| TNFRSF10B | 1.058371 | 6.51958 | 8.311805 | 3.41E-14 | 2.30E-12 | 21.76754 |
| CEBPD | 1.102005 | 8.811444 | 8.308018 | 3.49E-14 | 2.33E-12 | 21.7456 |
| LDLRAP1 | 1.024746 | 6.945713 | 8.273211 | 4.29E-14 | 2.79E-12 | 21.54416 |
| ATP6V1H | -1.08483 | 8.940483 | -8.27007 | 4.37E-14 | 2.83E-12 | 21.52602 |
| GRAMD3 | 1.281079 | 8.063827 | 8.266393 | 4.47E-14 | 2.88E-12 | 21.50475 |
| JPX | 1.573215 | 6.631774 | 8.246529 | 5.03E-14 | 3.20E-12 | 21.38998 |
| SLC22A3 | 1.276772 | 8.14936 | 8.23704 | 5.32E-14 | 3.36E-12 | 21.33518 |
| ATP6V1B2 | -1.33799 | 9.271223 | -8.21892 | 5.92E-14 | 3.70E-12 | 21.23066 |
| BMPR1B | 1.102528 | 8.3474 | 8.211042 | 6.20E-14 | 3.86E-12 | 21.18521 |
| MT1G | 1.271571 | 8.804335 | 8.210965 | 6.20E-14 | 3.86E-12 | 21.18477 |
| SCN2B | -1.47937 | 7.496801 | -8.20641 | 6.37E-14 | 3.94E-12 | 21.15853 |
| PTPRZ1 | 1.031283 | 11.06719 | 8.19961 | 6.63E-14 | 4.05E-12 | 21.11933 |
| SEPP1 | 1.381895 | 9.768767 | 8.199219 | 6.64E-14 | 4.05E-12 | 21.11708 |
| GPI | -1.31136 | 9.148857 | -8.18547 | 7.21E-14 | 4.36E-12 | 21.03787 |
| NUMBL | 1.159509 | 9.846822 | 8.185348 | 7.21E-14 | 4.36E-12 | 21.03719 |
| TUSC3 | -1.04775 | 6.506383 | -8.18192 | 7.36E-14 | 4.44E-12 | 21.01747 |
| FAR2 | -1.19709 | 6.722458 | -8.1481 | 8.98E-14 | 5.36E-12 | 20.82293 |
| METTL7A | 1.141919 | 9.485005 | 8.145167 | 9.14E-14 | 5.43E-12 | 20.80608 |
| KAT2B | 1.298142 | 9.25429 | 8.142263 | 9.29E-14 | 5.50E-12 | 20.7894 |
| LOC101060510 | 1.297762 | 7.038997 | 8.111628 | 1.11E-13 | 6.49E-12 | 20.61354 |
| SF3B5 | -1.0043 | 7.933913 | -8.10644 | 1.15E-13 | 6.66E-12 | 20.58376 |
| TFEB | 1.072062 | 6.5909 | 8.092088 | 1.25E-13 | 7.17E-12 | 20.50151 |
| SCRIB | 1.134827 | 8.456003 | 8.08619 | 1.29E-13 | 7.34E-12 | 20.46772 |
| MZT2B | 1.370841 | 9.148698 | 8.074452 | 1.38E-13 | 7.76E-12 | 20.40051 |
| ANLN | 1.173249 | 7.627183 | 8.071476 | 1.41E-13 | 7.88E-12 | 20.38347 |
| EIF2B3 | -1.26518 | 6.942171 | -8.06866 | 1.43E-13 | 7.97E-12 | 20.36734 |
| AQP4 | 1.253856 | 9.677773 | 8.066346 | 1.45E-13 | 8.04E-12 | 20.35411 |
| PTN | 1.323287 | 10.42723 | 8.049641 | 1.60E-13 | 8.81E-12 | 20.25856 |
| RTN3 | -1.14676 | 10.70873 | -8.04654 | 1.63E-13 | 8.93E-12 | 20.24083 |
| PSAT1 | 1.120889 | 9.313205 | 8.04464 | 1.65E-13 | 9.01E-12 | 20.22997 |
| RP11-73M18.8 | 1.06859 | 7.615756 | 8.043448 | 1.66E-13 | 9.05E-12 | 20.22316 |
| LOC286437 | 1.401196 | 8.532909 | 8.026694 | 1.83E-13 | 9.81E-12 | 20.12744 |
| SLC7A2 | 1.683002 | 6.534189 | 8.006637 | 2.06E-13 | 1.09E-11 | 20.01297 |
| CLDN15 | 1.012137 | 5.959522 | 8.006268 | 2.06E-13 | 1.09E-11 | 20.01086 |
| ELAVL3 | 1.332713 | 9.61907 | 8.003967 | 2.09E-13 | 1.10E-11 | 19.99774 |
| SLC1A6 | -1.32864 | 5.857876 | -7.988 | 2.29E-13 | 1.19E-11 | 19.90671 |
| RHOBTB3 | 1.035216 | 8.700264 | 7.985217 | 2.33E-13 | 1.20E-11 | 19.89085 |
| MT1F | 1.298994 | 10.15908 | 7.977392 | 2.44E-13 | 1.25E-11 | 19.84628 |
| SLIRP | -1.28475 | 8.926844 | -7.9742 | 2.49E-13 | 1.27E-11 | 19.82809 |
| LOC100130987 | 1.242928 | 7.599542 | 7.974085 | 2.49E-13 | 1.27E-11 | 19.82745 |
| GOT1 | -1.66516 | 7.898236 | -7.96616 | 2.61E-13 | 1.32E-11 | 19.7823 |
| FGF12 | -1.10304 | 7.279681 | -7.96117 | 2.68E-13 | 1.35E-11 | 19.7539 |
| SLC39A12 | 1.534816 | 8.172825 | 7.949661 | 2.87E-13 | 1.43E-11 | 19.68846 |
| CDC37 | -1.83712 | 5.625119 | -7.93916 | 3.05E-13 | 1.50E-11 | 19.62875 |
| CRYM | -1.06684 | 9.932471 | -7.93384 | 3.15E-13 | 1.55E-11 | 19.59855 |
| SLC38A2 | 1.04456 | 10.69466 | 7.921343 | 3.38E-13 | 1.65E-11 | 19.52755 |
| CTB-12A17.3 | 1.296781 | 9.797991 | 7.920042 | 3.41E-13 | 1.66E-11 | 19.52016 |
| RHPN2 | 1.321731 | 7.572315 | 7.912969 | 3.55E-13 | 1.72E-11 | 19.48001 |
| SERPINE2 | 1.030587 | 10.01564 | 7.912237 | 3.57E-13 | 1.72E-11 | 19.47586 |
| ARHGEF26 | 1.07016 | 7.353659 | 7.908099 | 3.65E-13 | 1.76E-11 | 19.45238 |
| JAZF1 | -1.04049 | 7.72099 | -7.90461 | 3.73E-13 | 1.79E-11 | 19.4326 |
| PSMA5 | -1.04039 | 9.035471 | -7.90165 | 3.79E-13 | 1.81E-11 | 19.41581 |
| ZIC1 | 1.812996 | 7.795479 | 7.901318 | 3.80E-13 | 1.81E-11 | 19.41391 |
| ZBTB20 | 1.29058 | 9.390703 | 7.898464 | 3.87E-13 | 1.84E-11 | 19.39773 |
| DYNC1I1 | -1.32247 | 9.50812 | -7.89705 | 3.90E-13 | 1.85E-11 | 19.3897 |
| HVCN1 | 1.433086 | 6.8571 | 7.893675 | 3.97E-13 | 1.88E-11 | 19.37057 |
| SDR16C5 | -1.55799 | 6.205245 | -7.88948 | 4.07E-13 | 1.91E-11 | 19.34677 |
| MSX1 | 1.140379 | 7.117116 | 7.883962 | 4.20E-13 | 1.97E-11 | 19.31553 |
| CALY | -1.76541 | 7.039531 | -7.87879 | 4.33E-13 | 2.02E-11 | 19.2862 |
| GRIN1 | -1.19348 | 6.339831 | -7.84649 | 5.23E-13 | 2.40E-11 | 19.10343 |
| TBC1D7 | -1.15056 | 6.353946 | -7.83705 | 5.52E-13 | 2.52E-11 | 19.0501 |
| MAP2K4 | -1.11481 | 8.599328 | -7.83657 | 5.53E-13 | 2.52E-11 | 19.04735 |
| DHCR24 | -1.33005 | 8.720841 | -7.81856 | 6.14E-13 | 2.77E-11 | 18.94564 |
| SULT4A1 | -1.25925 | 9.593636 | -7.7878 | 7.34E-13 | 3.24E-11 | 18.77216 |
| HSPA2 | 1.294617 | 9.262423 | 7.78289 | 7.55E-13 | 3.33E-11 | 18.74449 |
| RIN2 | 1.132517 | 8.081219 | 7.781341 | 7.62E-13 | 3.35E-11 | 18.73576 |
| GPRC5B | 1.06135 | 9.368113 | 7.762497 | 8.49E-13 | 3.71E-11 | 18.62967 |
| XAF1 | 1.206242 | 7.499983 | 7.757893 | 8.72E-13 | 3.80E-11 | 18.60377 |
| GJA1 | 1.47231 | 12.36708 | 7.752914 | 8.97E-13 | 3.89E-11 | 18.57576 |
| CHRM1 | -1.57675 | 7.919834 | -7.75288 | 8.97E-13 | 3.89E-11 | 18.57558 |
| ZFP36L2 | 1.099573 | 6.978643 | 7.750458 | 9.10E-13 | 3.94E-11 | 18.56195 |
| ZNF204P | -1.18489 | 8.341222 | -7.73954 | 9.69E-13 | 4.16E-11 | 18.50061 |
| LOC101927166 | 1.496379 | 8.478577 | 7.724504 | 1.06E-12 | 4.49E-11 | 18.41613 |
| RAB13 | 1.272172 | 9.320033 | 7.72333 | 1.06E-12 | 4.51E-11 | 18.40954 |
| XRCC2 | 1.143533 | 7.920242 | 7.71675 | 1.11E-12 | 4.67E-11 | 18.37261 |
| WWTR1 | 1.176149 | 6.664402 | 7.715347 | 1.11E-12 | 4.70E-11 | 18.36474 |
| EMX2 | 1.368565 | 8.529721 | 7.702249 | 1.20E-12 | 5.06E-11 | 18.29127 |
| SLC1A3 | 1.12101 | 11.34221 | 7.696792 | 1.24E-12 | 5.19E-11 | 18.26068 |
| NUDT2 | -1.63555 | 5.829152 | -7.69556 | 1.25E-12 | 5.22E-11 | 18.2538 |
| UQCRFS1 | -1.01933 | 9.160036 | -7.69263 | 1.27E-12 | 5.29E-11 | 18.23734 |
| RTN4IP1 | -1.61409 | 5.740146 | -7.66673 | 1.47E-12 | 6.04E-11 | 18.09236 |
| DHRS7B | -1.99072 | 5.670223 | -7.6454 | 1.66E-12 | 6.76E-11 | 17.97306 |
| GOLIM4 | 1.118779 | 8.272591 | 7.639983 | 1.72E-12 | 6.96E-11 | 17.94281 |
| LOC286367 | -1.60503 | 6.091806 | -7.62705 | 1.85E-12 | 7.45E-11 | 17.8706 |
| AMOTL2 | 1.070792 | 8.841394 | 7.60978 | 2.04E-12 | 8.14E-11 | 17.77427 |
| ATRNL1 | -1.10783 | 7.546735 | -7.60512 | 2.10E-12 | 8.33E-11 | 17.74828 |
| ATP6V1E1 | -1.24576 | 10.59604 | -7.59339 | 2.24E-12 | 8.82E-11 | 17.68292 |
| MDH1 | -1.49375 | 11.74451 | -7.58945 | 2.29E-12 | 8.97E-11 | 17.66101 |
| EFHD1 | 1.169238 | 9.782114 | 7.584873 | 2.35E-12 | 9.18E-11 | 17.63551 |
| GPER1 | 1.322605 | 6.192373 | 7.58265 | 2.38E-12 | 9.26E-11 | 17.62314 |
| SDC4 | 1.290161 | 9.37205 | 7.554267 | 2.80E-12 | 1.07E-10 | 17.4653 |
| MT1E | 1.081407 | 10.09802 | 7.55335 | 2.82E-12 | 1.08E-10 | 17.46021 |
| TMEM123 | 1.178898 | 8.381052 | 7.537693 | 3.08E-12 | 1.16E-10 | 17.37326 |
| NFIC | 1.305959 | 9.116781 | 7.525083 | 3.31E-12 | 1.24E-10 | 17.30331 |
| GABRG2 | -1.26207 | 9.548974 | -7.5161 | 3.48E-12 | 1.30E-10 | 17.25349 |
| EEF1A2 | -1.34326 | 8.620489 | -7.51438 | 3.52E-12 | 1.31E-10 | 17.24396 |
| FGFR3 | 1.37656 | 10.47097 | 7.507765 | 3.65E-12 | 1.34E-10 | 17.20731 |
| FHL2 | -1.1378 | 7.509087 | -7.49132 | 4.01E-12 | 1.47E-10 | 17.11625 |
| CNOT10 | -1.45706 | 5.477504 | -7.47669 | 4.35E-12 | 1.58E-10 | 17.03532 |
| GPAM | 1.139967 | 8.360469 | 7.469804 | 4.53E-12 | 1.64E-10 | 16.99729 |
| COPS3 | -1.15804 | 7.750999 | -7.46242 | 4.72E-12 | 1.70E-10 | 16.95648 |
| ZIC2 | 1.495769 | 8.603679 | 7.43634 | 5.47E-12 | 1.96E-10 | 16.81256 |
| SV2B | -1.18958 | 10.08678 | -7.43222 | 5.60E-12 | 2.00E-10 | 16.78987 |
| SDHB | -1.09593 | 6.347076 | -7.43012 | 5.67E-12 | 2.02E-10 | 16.77826 |
| ELTD1 | 1.462833 | 5.639397 | 7.425932 | 5.80E-12 | 2.05E-10 | 16.75519 |
| SLC26A6 | 1.106666 | 7.31179 | 7.42307 | 5.90E-12 | 2.08E-10 | 16.73942 |
| PECAM1 | 1.052774 | 6.95533 | 7.42051 | 5.98E-12 | 2.10E-10 | 16.72532 |
| HSP90B1 | 1.273577 | 8.288894 | 7.405595 | 6.51E-12 | 2.27E-10 | 16.6432 |
| CHGB | -1.7304 | 9.405581 | -7.40017 | 6.71E-12 | 2.33E-10 | 16.61333 |
| NRXN3 | -1.29362 | 9.109424 | -7.38002 | 7.52E-12 | 2.59E-10 | 16.50262 |
| MEG3 | 1.108429 | 11.41231 | 7.372599 | 7.84E-12 | 2.69E-10 | 16.46183 |
| TBC1D9 | -1.0114 | 8.383412 | -7.349 | 8.96E-12 | 3.03E-10 | 16.33238 |
| TNS3 | 1.31591 | 9.642144 | 7.344157 | 9.20E-12 | 3.10E-10 | 16.30582 |
| RWDD2B | -1.26823 | 6.941169 | -7.34383 | 9.22E-12 | 3.10E-10 | 16.30401 |
| SRGN | 1.2744 | 7.73243 | 7.342343 | 9.30E-12 | 3.13E-10 | 16.29588 |
| PON2 | 1.157994 | 9.667723 | 7.340411 | 9.40E-12 | 3.16E-10 | 16.28529 |
| IL17RB | 1.092538 | 8.001452 | 7.336282 | 9.62E-12 | 3.21E-10 | 16.26267 |
| SPP1 | 1.181917 | 8.835423 | 7.334763 | 9.70E-12 | 3.23E-10 | 16.25435 |
| IRF2BPL | 1.065172 | 11.62583 | 7.33473 | 9.70E-12 | 3.23E-10 | 16.25417 |
| ND6 | 1.367375 | 11.50103 | 7.328702 | 1.00E-11 | 3.33E-10 | 16.22116 |
| AGAP9 | 1.507111 | 5.639026 | 7.32529 | 1.02E-11 | 3.39E-10 | 16.20249 |
| NOMO3 | 1.586789 | 5.901847 | 7.320215 | 1.05E-11 | 3.49E-10 | 16.17471 |
| RP11-416I2.1 | -1.29613 | 6.495736 | -7.31772 | 1.07E-11 | 3.53E-10 | 16.16106 |
| PCSK5 | 1.019752 | 7.331007 | 7.315431 | 1.08E-11 | 3.57E-10 | 16.14854 |
| NRN1 | -1.04815 | 9.713751 | -7.31529 | 1.08E-11 | 3.57E-10 | 16.14777 |
| RHOU | 1.01913 | 8.6634 | 7.308471 | 1.12E-11 | 3.69E-10 | 16.11048 |
| CA10 | -1.14677 | 8.178363 | -7.30813 | 1.13E-11 | 3.69E-10 | 16.10861 |
| JAZF1-AS1 | -1.85227 | 5.366892 | -7.29872 | 1.19E-11 | 3.87E-10 | 16.0572 |
| SSBP3-AS1 | 1.242637 | 7.661275 | 7.297455 | 1.20E-11 | 3.89E-10 | 16.05028 |
| RAB3C | -1.3597 | 7.083453 | -7.2971 | 1.20E-11 | 3.89E-10 | 16.04835 |
| FIBP | -1.03259 | 8.312895 | -7.29484 | 1.21E-11 | 3.94E-10 | 16.03598 |
| RP11-373D23.2 | 1.241682 | 6.470017 | 7.293873 | 1.22E-11 | 3.94E-10 | 16.03072 |
| FYCO1 | 1.006322 | 6.794963 | 7.289868 | 1.25E-11 | 4.02E-10 | 16.00885 |
| RP11-334C17.5 | 1.700268 | 6.217935 | 7.272282 | 1.38E-11 | 4.39E-10 | 15.91289 |
| STAT4 | -1.47892 | 7.932099 | -7.26658 | 1.42E-11 | 4.52E-10 | 15.88178 |
| NIT2 | -1.12159 | 7.408908 | -7.26518 | 1.43E-11 | 4.54E-10 | 15.87419 |
| SUSD4 | -1.06331 | 7.376055 | -7.25876 | 1.49E-11 | 4.70E-10 | 15.83916 |
| MT1H | 1.330978 | 10.07143 | 7.253763 | 1.53E-11 | 4.82E-10 | 15.81196 |
| AK090844 | 1.197353 | 9.051756 | 7.250119 | 1.56E-11 | 4.89E-10 | 15.79212 |
| LOC100131541 | 1.819433 | 5.56061 | 7.247995 | 1.58E-11 | 4.94E-10 | 15.78056 |
| NXN | 1.007295 | 8.181236 | 7.247542 | 1.58E-11 | 4.94E-10 | 15.77809 |
| NECAP1 | -1.21963 | 8.951999 | -7.24694 | 1.59E-11 | 4.95E-10 | 15.7748 |
| GABRA4 | -1.15786 | 7.644108 | -7.22235 | 1.82E-11 | 5.58E-10 | 15.6411 |
| C1QTNF4 | -2.11112 | 5.894191 | -7.2133 | 1.92E-11 | 5.81E-10 | 15.59189 |
| OSMR | 1.033563 | 6.465115 | 7.211786 | 1.93E-11 | 5.84E-10 | 15.58369 |
| LOC728061 | 1.216234 | 7.711979 | 7.209039 | 1.96E-11 | 5.91E-10 | 15.56878 |
| LOC100130429 | 1.653546 | 6.94796 | 7.186239 | 2.23E-11 | 6.63E-10 | 15.44509 |
| MT1X | 1.216154 | 10.29441 | 7.177484 | 2.34E-11 | 6.92E-10 | 15.39765 |
| RAB31 | 1.126892 | 9.918551 | 7.175137 | 2.37E-11 | 7.00E-10 | 15.38494 |
| CAMK1G | -1.33942 | 6.422077 | -7.15902 | 2.59E-11 | 7.58E-10 | 15.29768 |
| RP11-395B7.7 | 1.240431 | 5.984832 | 7.158886 | 2.59E-11 | 7.58E-10 | 15.29698 |
| PIDD1 | 1.021107 | 6.997152 | 7.155325 | 2.64E-11 | 7.72E-10 | 15.27771 |
| CDK7 | -1.7232 | 6.710643 | -7.15123 | 2.71E-11 | 7.88E-10 | 15.25557 |
| GABRG1 | 1.114738 | 7.79458 | 7.140522 | 2.87E-11 | 8.32E-10 | 15.1977 |
| LOC439911 | 1.04428 | 7.542861 | 7.13728 | 2.92E-11 | 8.44E-10 | 15.18019 |
| ACKR3 | 1.268922 | 7.058123 | 7.134949 | 2.96E-11 | 8.54E-10 | 15.1676 |
| BC022892 | 1.619439 | 4.741187 | 7.132255 | 3.01E-11 | 8.63E-10 | 15.15305 |
| LOC100134445 | 1.101789 | 8.535434 | 7.122988 | 3.16E-11 | 8.99E-10 | 15.10304 |
| LARGE | -1.05221 | 7.264649 | -7.1039 | 3.52E-11 | 9.87E-10 | 15.00014 |
| LOC646014 | 1.228852 | 8.085558 | 7.103744 | 3.52E-11 | 9.87E-10 | 14.99928 |
| FAM198B | 1.155054 | 8.60465 | 7.098027 | 3.63E-11 | 1.02E-09 | 14.96848 |
| CHCHD6 | -1.29516 | 7.376348 | -7.09471 | 3.70E-11 | 1.03E-09 | 14.95063 |
| BCAS1 | 1.631838 | 8.377373 | 7.086656 | 3.87E-11 | 1.07E-09 | 14.90727 |
| KLF2 | 1.062187 | 6.970764 | 7.072265 | 4.19E-11 | 1.16E-09 | 14.82987 |
| C11orf73 | -1.17612 | 6.962613 | -7.06482 | 4.36E-11 | 1.20E-09 | 14.78988 |
| CRMP1 | -1.01736 | 9.098997 | -7.06323 | 4.40E-11 | 1.21E-09 | 14.7813 |
| PCAT19 | 1.417779 | 6.031113 | 7.054757 | 4.61E-11 | 1.26E-09 | 14.73582 |
| SLAIN1 | 1.027163 | 9.003834 | 7.041684 | 4.96E-11 | 1.34E-09 | 14.66567 |
| VASN | 1.05763 | 6.660606 | 7.039918 | 5.01E-11 | 1.35E-09 | 14.6562 |
| CUL1 | -1.01525 | 6.887756 | -7.03067 | 5.27E-11 | 1.41E-09 | 14.6066 |
| KIF1C | 1.080287 | 6.593605 | 7.017918 | 5.65E-11 | 1.51E-09 | 14.53832 |
| MPV17 | -1.45722 | 6.383296 | -7.01791 | 5.65E-11 | 1.51E-09 | 14.53831 |
| SYN2 | -1.10615 | 9.342952 | -7.01067 | 5.88E-11 | 1.56E-09 | 14.49955 |
| RRAGB | -1.65488 | 5.006787 | -7.00856 | 5.95E-11 | 1.58E-09 | 14.48826 |
| MIR7-3HG | -1.30755 | 6.37895 | -7.00705 | 6.00E-11 | 1.59E-09 | 14.48015 |
| COPS5 | -1.00439 | 8.466343 | -6.9863 | 6.72E-11 | 1.76E-09 | 14.36927 |
| GABRA1 | -1.40475 | 9.288077 | -6.97791 | 7.04E-11 | 1.83E-09 | 14.32448 |
| BC045805 | 1.243737 | 7.380756 | 6.976071 | 7.11E-11 | 1.84E-09 | 14.31465 |
| ID2 | 1.022924 | 9.438924 | 6.972929 | 7.24E-11 | 1.87E-09 | 14.29789 |
| NKX2-2 | 1.384564 | 6.379863 | 6.961844 | 7.69E-11 | 1.98E-09 | 14.23877 |
| CARTPT | -1.18025 | 7.682335 | -6.96121 | 7.72E-11 | 1.99E-09 | 14.23537 |
| NVL | -1.83256 | 5.232241 | -6.95618 | 7.93E-11 | 2.03E-09 | 14.20861 |
| LOC100289019 | 1.032045 | 7.657107 | 6.949213 | 8.24E-11 | 2.10E-09 | 14.17148 |
| MAML2 | 1.023525 | 7.250835 | 6.947314 | 8.33E-11 | 2.12E-09 | 14.16136 |
| SERTM1 | -1.41444 | 7.02976 | -6.94467 | 8.45E-11 | 2.15E-09 | 14.14726 |
| SLC25A18 | 1.372294 | 9.972032 | 6.944321 | 8.46E-11 | 2.15E-09 | 14.14543 |
| TM7SF2 | -1.25362 | 6.170927 | -6.93856 | 8.73E-11 | 2.21E-09 | 14.11475 |
| DYRK4 | -1.06102 | 7.122084 | -6.9137 | 1.00E-10 | 2.50E-09 | 13.9826 |
| DTX3L | 1.104972 | 6.967818 | 6.901423 | 1.07E-10 | 2.65E-09 | 13.91745 |
| MAL2 | -1.54847 | 8.670141 | -6.89814 | 1.09E-10 | 2.69E-09 | 13.90003 |
| SURF2 | -1.30755 | 5.010858 | -6.89778 | 1.09E-10 | 2.69E-09 | 13.89814 |
| LOC100289283 | 1.286352 | 6.874188 | 6.891529 | 1.13E-10 | 2.78E-09 | 13.86497 |
| KIFAP3 | -1.46746 | 7.973594 | -6.89028 | 1.14E-10 | 2.80E-09 | 13.85837 |
| C21orf91 | 1.102482 | 8.082921 | 6.879793 | 1.20E-10 | 2.95E-09 | 13.80279 |
| FOXC1 | 1.582186 | 7.892025 | 6.862398 | 1.32E-10 | 3.21E-09 | 13.71072 |
| RP11-157P1.4 | 1.224682 | 6.304317 | 6.859102 | 1.35E-10 | 3.26E-09 | 13.69329 |
| LOC100129447 | 1.118178 | 7.851218 | 6.855696 | 1.37E-10 | 3.30E-09 | 13.67528 |
| VASP | 1.218307 | 6.162941 | 6.837923 | 1.51E-10 | 3.62E-09 | 13.58139 |
| MTX2 | -1.23852 | 8.074735 | -6.8357 | 1.53E-10 | 3.65E-09 | 13.56966 |
| OLIG1 | 1.254013 | 10.6346 | 6.826092 | 1.61E-10 | 3.84E-09 | 13.51897 |
| RP11-1114A5.4 | 1.144997 | 6.297166 | 6.823685 | 1.63E-10 | 3.88E-09 | 13.50628 |
| STMN2 | -1.25133 | 11.33687 | -6.80925 | 1.77E-10 | 4.16E-09 | 13.4302 |
| HPRT1 | -1.21327 | 9.138472 | -6.80851 | 1.77E-10 | 4.17E-09 | 13.42629 |
| ZNF566 | 1.336383 | 6.151907 | 6.808184 | 1.78E-10 | 4.18E-09 | 13.4246 |
| WDR70 | -1.04091 | 6.576402 | -6.80678 | 1.79E-10 | 4.20E-09 | 13.41722 |
| IMPDH2 | -1.0561 | 8.400238 | -6.79397 | 1.92E-10 | 4.49E-09 | 13.34979 |
| ID3 | 1.920755 | 6.199507 | 6.78708 | 1.99E-10 | 4.65E-09 | 13.31355 |
| ARPC1A | -1.86505 | 6.793843 | -6.77884 | 2.08E-10 | 4.84E-09 | 13.27027 |
| RP11-389C8.2 | 1.879813 | 6.123026 | 6.765255 | 2.24E-10 | 5.18E-09 | 13.19892 |
| C11orf96 | 1.384119 | 10.13657 | 6.751168 | 2.42E-10 | 5.56E-09 | 13.12504 |
| APOO | -1.2342 | 8.199946 | -6.74553 | 2.49E-10 | 5.71E-09 | 13.09551 |
| FAM71E1 | -1.55164 | 4.833323 | -6.74473 | 2.50E-10 | 5.73E-09 | 13.0913 |
| CLPP | -1.36666 | 6.642454 | -6.74103 | 2.55E-10 | 5.82E-09 | 13.07193 |
| LDHA | -1.17511 | 10.98688 | -6.7407 | 2.56E-10 | 5.82E-09 | 13.07018 |
| RP11-357G3.2 | 1.863742 | 5.523746 | 6.727128 | 2.75E-10 | 6.21E-09 | 12.99916 |
| ITGA6 | 1.036326 | 6.700634 | 6.711477 | 2.99E-10 | 6.71E-09 | 12.91734 |
| PPIH | -1.11568 | 7.09928 | -6.70343 | 3.13E-10 | 6.97E-09 | 12.87531 |
| AMPH | -1.3634 | 9.359188 | -6.70241 | 3.14E-10 | 6.99E-09 | 12.86999 |
| KLF4 | 1.322098 | 6.378977 | 6.702183 | 3.15E-10 | 6.99E-09 | 12.86881 |
| BEX4 | -1.0305 | 9.701739 | -6.69807 | 3.22E-10 | 7.14E-09 | 12.84736 |
| SMYD3 | -1.21105 | 7.387845 | -6.69332 | 3.30E-10 | 7.29E-09 | 12.82255 |
| TFAP2C | 1.203478 | 4.645035 | 6.692662 | 3.31E-10 | 7.31E-09 | 12.81913 |
| AHNAK | 1.102156 | 6.700223 | 6.681763 | 3.51E-10 | 7.70E-09 | 12.7623 |
| CARD6 | 1.416895 | 5.26389 | 6.676815 | 3.61E-10 | 7.86E-09 | 12.73653 |
| PPEF1 | -1.60543 | 5.600613 | -6.66905 | 3.76E-10 | 8.13E-09 | 12.69611 |
| PRO2852 | 1.197113 | 6.576881 | 6.663635 | 3.87E-10 | 8.34E-09 | 12.66791 |
| DNTTIP1 | -1.24151 | 5.463894 | -6.65473 | 4.06E-10 | 8.68E-09 | 12.62161 |
| EPS8 | 1.104115 | 7.753744 | 6.653097 | 4.09E-10 | 8.75E-09 | 12.6131 |
| ARHGEF1 | 1.009296 | 7.750989 | 6.649206 | 4.18E-10 | 8.91E-09 | 12.59288 |
| PTPRO | 1.114478 | 12.10235 | 6.63811 | 4.43E-10 | 9.40E-09 | 12.53524 |
| PLP1 | 1.078074 | 11.4408 | 6.615351 | 5.01E-10 | 1.05E-08 | 12.4172 |
| CAPNS1 | -1.24589 | 7.906869 | -6.59791 | 5.50E-10 | 1.14E-08 | 12.32691 |
| KDM1A | -1.13473 | 7.09954 | -6.59459 | 5.59E-10 | 1.16E-08 | 12.30971 |
| GAREML | 1.087449 | 6.720082 | 6.590779 | 5.71E-10 | 1.18E-08 | 12.29001 |
| LOC102724356 | 1.093328 | 9.256694 | 6.578783 | 6.09E-10 | 1.25E-08 | 12.22802 |
| BLVRB | -1.28118 | 6.85916 | -6.57332 | 6.26E-10 | 1.29E-08 | 12.19982 |
| BEX5 | -1.07013 | 11.52173 | -6.56866 | 6.42E-10 | 1.31E-08 | 12.17575 |
| CCK | -1.45415 | 9.58333 | -6.56646 | 6.50E-10 | 1.32E-08 | 12.16439 |
| AX747507 | 1.363354 | 6.568712 | 6.562894 | 6.62E-10 | 1.34E-08 | 12.14601 |
| NUDT18 | -1.28241 | 5.944307 | -6.55342 | 6.96E-10 | 1.41E-08 | 12.09716 |
| MYBPC1 | 1.043328 | 8.859338 | 6.547646 | 7.18E-10 | 1.45E-08 | 12.06741 |
| CLEC2L | -1.18926 | 6.99577 | -6.5421 | 7.39E-10 | 1.49E-08 | 12.03887 |
| TUBA4A | -1.05125 | 10.85033 | -6.53568 | 7.65E-10 | 1.53E-08 | 12.00579 |
| F3 | 1.315331 | 8.96166 | 6.531888 | 7.81E-10 | 1.56E-08 | 11.9863 |
| MRVI1 | 1.011858 | 8.655909 | 6.529418 | 7.91E-10 | 1.58E-08 | 11.9736 |
| APOLD1 | 1.093172 | 9.126319 | 6.528285 | 7.96E-10 | 1.58E-08 | 11.96777 |
| PAXIP1OS | 1.044713 | 5.313781 | 6.521506 | 8.25E-10 | 1.64E-08 | 11.93292 |
| HSP90AB1 | -1.02825 | 9.086524 | -6.51804 | 8.40E-10 | 1.66E-08 | 11.9151 |
| TNFRSF1A | 1.191187 | 7.118623 | 6.515625 | 8.51E-10 | 1.68E-08 | 11.90271 |
| RGS7 | -1.03408 | 9.593454 | -6.51193 | 8.67E-10 | 1.71E-08 | 11.88372 |
| C19orf18 | 1.036889 | 5.889109 | 6.511547 | 8.69E-10 | 1.71E-08 | 11.88176 |
| ITGAV | 1.023708 | 9.9286 | 6.502113 | 9.14E-10 | 1.79E-08 | 11.83335 |
| LAMB2 | 1.119612 | 7.177572 | 6.497886 | 9.34E-10 | 1.82E-08 | 11.81166 |
| WDR7 | -1.0204 | 9.250659 | -6.49127 | 9.68E-10 | 1.88E-08 | 11.77773 |
| MIR302B | 1.018072 | 6.59242 | 6.485209 | 9.99E-10 | 1.93E-08 | 11.74669 |
| STAR | -1.35056 | 5.667466 | -6.48005 | 1.03E-09 | 1.98E-08 | 11.72025 |
| PSMG1 | -1.02778 | 7.323124 | -6.4778 | 1.04E-09 | 2.00E-08 | 11.70875 |
| ROMO1 | -1.12771 | 7.679698 | -6.46571 | 1.11E-09 | 2.12E-08 | 11.64691 |
| CUX1 | 1.065319 | 8.69365 | 6.464021 | 1.12E-09 | 2.13E-08 | 11.63826 |
| HMP19 | -1.0573 | 9.851563 | -6.46341 | 1.12E-09 | 2.13E-08 | 11.63512 |
| AEBP1 | 1.283152 | 6.707151 | 6.462504 | 1.13E-09 | 2.14E-08 | 11.63051 |
| PRMT7 | -1.59225 | 5.758706 | -6.45385 | 1.18E-09 | 2.22E-08 | 11.58631 |
| CECR6 | -1.1994 | 6.595598 | -6.45368 | 1.18E-09 | 2.22E-08 | 11.58545 |
| RNF181 | -1.07295 | 6.66097 | -6.45106 | 1.20E-09 | 2.25E-08 | 11.57206 |
| PAIP2B | 1.172733 | 7.576004 | 6.445387 | 1.23E-09 | 2.31E-08 | 11.54308 |
| ZWINT | -1.09381 | 7.251255 | -6.42949 | 1.34E-09 | 2.50E-08 | 11.46202 |
| LAMP5 | -1.27405 | 8.775554 | -6.42483 | 1.37E-09 | 2.55E-08 | 11.43826 |
| PBXIP1 | 1.032158 | 8.083284 | 6.418618 | 1.42E-09 | 2.63E-08 | 11.40663 |
| MROH6 | 1.230635 | 6.987672 | 6.415593 | 1.44E-09 | 2.66E-08 | 11.39123 |
| CALB1 | -1.21062 | 7.706505 | -6.41275 | 1.46E-09 | 2.70E-08 | 11.37677 |
| C14orf37 | 1.0642 | 7.437918 | 6.411969 | 1.47E-09 | 2.70E-08 | 11.37278 |
| MECR | -1.28286 | 6.017851 | -6.40938 | 1.49E-09 | 2.74E-08 | 11.35962 |
| RP11-690I21.2 | 1.118034 | 9.170611 | 6.403149 | 1.54E-09 | 2.82E-08 | 11.32793 |
| CRB2 | 1.425077 | 5.289469 | 6.394618 | 1.61E-09 | 2.94E-08 | 11.28457 |
| NCALD | -1.11933 | 8.412211 | -6.39183 | 1.63E-09 | 2.98E-08 | 11.27043 |
| LPAR1 | 1.078225 | 8.554853 | 6.387458 | 1.67E-09 | 3.04E-08 | 11.24821 |
| ZBBX | -1.22726 | 6.580115 | -6.37739 | 1.76E-09 | 3.19E-08 | 11.19712 |
| IGFBP7 | 1.059599 | 9.590625 | 6.375074 | 1.78E-09 | 3.22E-08 | 11.18538 |
| PPM1E | -1.32542 | 8.986909 | -6.37325 | 1.80E-09 | 3.25E-08 | 11.17614 |
| ACTL6B | -1.42648 | 7.048033 | -6.35856 | 1.94E-09 | 3.49E-08 | 11.10173 |
| EMC9 | -1.38774 | 6.385651 | -6.35781 | 1.95E-09 | 3.50E-08 | 11.09792 |
| SLC18B1 | 1.047499 | 9.323113 | 6.35193 | 2.01E-09 | 3.58E-08 | 11.06815 |
| USP39 | -1.24554 | 5.456635 | -6.35105 | 2.02E-09 | 3.60E-08 | 11.06371 |
| FAM107A | 1.179193 | 11.34729 | 6.339757 | 2.14E-09 | 3.80E-08 | 11.0066 |
| ANKRD36B | 1.410953 | 11.42015 | 6.328017 | 2.28E-09 | 4.01E-08 | 10.9473 |
| AIF1L | 1.057632 | 7.366523 | 6.303995 | 2.58E-09 | 4.49E-08 | 10.82617 |
| EPAS1 | 1.203487 | 8.745676 | 6.296597 | 2.68E-09 | 4.66E-08 | 10.78893 |
| RP11-295G20.2 | 1.115058 | 4.856142 | 6.2755 | 2.99E-09 | 5.14E-08 | 10.68285 |
| AF289551 | 1.436328 | 5.260522 | 6.271081 | 3.06E-09 | 5.26E-08 | 10.66066 |
| HSPB3 | -1.516 | 5.878922 | -6.25916 | 3.26E-09 | 5.56E-08 | 10.60082 |
| RUVBL1 | -1.26293 | 5.303555 | -6.25486 | 3.33E-09 | 5.67E-08 | 10.57929 |
| TBC1D19 | -1.49936 | 5.241563 | -6.25208 | 3.38E-09 | 5.73E-08 | 10.56533 |
| UQCRC1 | -1.3791 | 8.988866 | -6.23412 | 3.71E-09 | 6.22E-08 | 10.47545 |
| LOC101930415 | 1.147472 | 9.237209 | 6.233654 | 3.71E-09 | 6.23E-08 | 10.4731 |
| RP11-69I8.2 | 1.086822 | 5.791753 | 6.2279 | 3.83E-09 | 6.40E-08 | 10.44432 |
| LOC100507557 | -1.04865 | 7.274186 | -6.22543 | 3.88E-09 | 6.47E-08 | 10.43196 |
| PDK4 | 1.077821 | 7.574603 | 6.221838 | 3.95E-09 | 6.57E-08 | 10.41402 |
| TARBP1 | -1.2227 | 8.184532 | -6.21593 | 4.07E-09 | 6.74E-08 | 10.38449 |
| C14orf119 | -1.1744 | 6.985259 | -6.21288 | 4.13E-09 | 6.84E-08 | 10.3693 |
| SERPINF1 | -1.29142 | 7.067949 | -6.20251 | 4.36E-09 | 7.17E-08 | 10.31752 |
| LOC102724870 | 1.137907 | 10.14779 | 6.182707 | 4.83E-09 | 7.86E-08 | 10.21888 |
| LOC100133089 | 1.321588 | 7.017408 | 6.17901 | 4.92E-09 | 8.00E-08 | 10.20048 |
| ABT1 | -1.41932 | 5.186855 | -6.17744 | 4.96E-09 | 8.05E-08 | 10.19265 |
| GLRX | -1.16742 | 8.355078 | -6.17682 | 4.98E-09 | 8.06E-08 | 10.18957 |
| LINC00889 | -1.46872 | 6.226327 | -6.16178 | 5.38E-09 | 8.61E-08 | 10.11485 |
| LRRC32 | 1.346696 | 5.742431 | 6.154707 | 5.57E-09 | 8.89E-08 | 10.07972 |
| SLC27A4 | -1.21161 | 6.201 | -6.15396 | 5.60E-09 | 8.92E-08 | 10.07602 |
| ATOX1 | -1.07716 | 7.469335 | -6.14417 | 5.88E-09 | 9.32E-08 | 10.02744 |
| RP11-589P10.5 | 1.189506 | 7.20571 | 6.139607 | 6.02E-09 | 9.50E-08 | 10.00483 |
| TMEM191A | -1.29932 | 5.767904 | -6.12554 | 6.47E-09 | 1.01E-07 | 9.93517 |
| ST6GALNAC5 | -1.07287 | 7.837711 | -6.12334 | 6.54E-09 | 1.02E-07 | 9.924279 |
| SNX31 | 1.716989 | 5.443484 | 6.12158 | 6.60E-09 | 1.03E-07 | 9.915576 |
| GNG3 | -1.08973 | 9.345245 | -6.1198 | 6.66E-09 | 1.04E-07 | 9.906781 |
| SNX10 | -1.10055 | 9.755503 | -6.09789 | 7.45E-09 | 1.15E-07 | 9.79851 |
| IFITM1 | 1.004893 | 8.867115 | 6.079907 | 8.17E-09 | 1.24E-07 | 9.709874 |
| LSM7 | -1.06 | 7.126281 | -6.0645 | 8.83E-09 | 1.33E-07 | 9.634067 |
| HACL1 | -1.0369 | 6.73081 | -6.06096 | 8.99E-09 | 1.35E-07 | 9.616638 |
| LOC283484 | -1.0045 | 5.374238 | -6.04531 | 9.74E-09 | 1.45E-07 | 9.539757 |
| RPA3 | -1.59033 | 5.448436 | -6.04465 | 9.77E-09 | 1.46E-07 | 9.536534 |
| SEPSECS-AS1 | 1.069723 | 5.983481 | 6.043695 | 9.82E-09 | 1.46E-07 | 9.531843 |
| YBX3 | 1.002069 | 6.914226 | 6.034656 | 1.03E-08 | 1.52E-07 | 9.48751 |
| MIF | -1.06473 | 9.456559 | -6.02777 | 1.06E-08 | 1.57E-07 | 9.453761 |
| ERICH3 | -1.2429 | 8.324936 | -6.02752 | 1.07E-08 | 1.57E-07 | 9.45254 |
| SMYD5 | -1.24094 | 6.031036 | -6.01834 | 1.12E-08 | 1.63E-07 | 9.407589 |
| MRPL37 | -1.34547 | 6.050647 | -6.01542 | 1.13E-08 | 1.66E-07 | 9.39332 |
| PRO1804 | 1.422141 | 6.857354 | 6.015231 | 1.13E-08 | 1.66E-07 | 9.392379 |
| IGFBPL1 | -1.18652 | 5.972823 | -6.00949 | 1.17E-08 | 1.70E-07 | 9.364292 |
| ZNF57 | -1.16787 | 6.397784 | -6.00798 | 1.18E-08 | 1.72E-07 | 9.356906 |
| BC041025 | 1.428832 | 6.011434 | 6.005407 | 1.19E-08 | 1.74E-07 | 9.344341 |
| CSPG4 | 1.033268 | 5.888384 | 5.990742 | 1.28E-08 | 1.86E-07 | 9.272718 |
| PRR3 | -1.3253 | 4.965195 | -5.99059 | 1.28E-08 | 1.86E-07 | 9.271988 |
| FGR | 1.26974 | 6.136641 | 5.981331 | 1.35E-08 | 1.94E-07 | 9.226818 |
| LOC100134822 | 1.142333 | 5.763227 | 5.976497 | 1.38E-08 | 1.98E-07 | 9.203262 |
| UBE2T | -1.33785 | 5.474182 | -5.97334 | 1.40E-08 | 2.01E-07 | 9.187879 |
| SSSCA1 | -1.52011 | 4.39896 | -5.9731 | 1.40E-08 | 2.01E-07 | 9.186706 |
| ATOH7 | -1.07957 | 5.759536 | -5.96039 | 1.49E-08 | 2.13E-07 | 9.124835 |
| DDIT4L | 1.172338 | 7.450614 | 5.950224 | 1.57E-08 | 2.22E-07 | 9.075429 |
| KCNJ16 | 1.07532 | 7.033746 | 5.941044 | 1.65E-08 | 2.31E-07 | 9.030847 |
| RP11-271C24.3 | 1.031354 | 7.853292 | 5.9407 | 1.65E-08 | 2.32E-07 | 9.029178 |
| SOX2-OT | 1.16215 | 9.398578 | 5.929374 | 1.75E-08 | 2.43E-07 | 8.974236 |
| SGIP1 | -1.19845 | 8.269795 | -5.92825 | 1.76E-08 | 2.45E-07 | 8.968767 |
| MRPS28 | -1.04473 | 6.903177 | -5.92431 | 1.79E-08 | 2.49E-07 | 8.949698 |
| HEPH | 1.023975 | 7.880319 | 5.916509 | 1.86E-08 | 2.58E-07 | 8.911916 |
| LEAP2 | 1.17703 | 6.336042 | 5.910385 | 1.92E-08 | 2.65E-07 | 8.882281 |
| CXCL1 | 1.234539 | 5.660011 | 5.907333 | 1.95E-08 | 2.69E-07 | 8.867514 |
| RPH3A | -1.05546 | 8.815757 | -5.8923 | 2.10E-08 | 2.88E-07 | 8.794854 |
| GABRD | -1.50337 | 6.726591 | -5.85829 | 2.49E-08 | 3.36E-07 | 8.630972 |
| SLCO4A1 | 1.022709 | 6.902666 | 5.848306 | 2.62E-08 | 3.51E-07 | 8.582976 |
| MAEL | -1.23561 | 5.306102 | -5.84672 | 2.64E-08 | 3.54E-07 | 8.575342 |
| ATP6AP1 | -1.1219 | 8.802405 | -5.83446 | 2.80E-08 | 3.73E-07 | 8.516507 |
| CCNA1 | -1.1718 | 6.897675 | -5.82084 | 3.00E-08 | 3.98E-07 | 8.451208 |
| C11orf97 | -1.1345 | 3.50163 | -5.81845 | 3.04E-08 | 4.02E-07 | 8.439749 |
| LATS2 | 1.113777 | 7.097912 | 5.817831 | 3.05E-08 | 4.03E-07 | 8.436796 |
| PHYHD1 | 1.101728 | 6.393443 | 5.812354 | 3.13E-08 | 4.12E-07 | 8.410576 |
| PSMC4 | -1.07128 | 6.855837 | -5.80491 | 3.25E-08 | 4.27E-07 | 8.37496 |
| ENC1 | -1.23144 | 11.00032 | -5.80125 | 3.31E-08 | 4.33E-07 | 8.357492 |
| CHST6 | 1.564872 | 4.41574 | 5.783449 | 3.61E-08 | 4.71E-07 | 8.272477 |
| RP11-38P22.2 | 1.086192 | 8.228402 | 5.781553 | 3.64E-08 | 4.74E-07 | 8.263432 |
| LOC100506563 | -1.08802 | 6.046941 | -5.77907 | 3.69E-08 | 4.79E-07 | 8.251594 |
| COL1A2 | 1.005796 | 6.006667 | 5.776206 | 3.74E-08 | 4.85E-07 | 8.237942 |
| C15orf52 | 1.155485 | 6.388894 | 5.775979 | 3.75E-08 | 4.85E-07 | 8.236861 |
| LRMP | -1.04957 | 5.943778 | -5.77239 | 3.81E-08 | 4.92E-07 | 8.219752 |
| PPP1R14C | -1.11966 | 6.878372 | -5.77099 | 3.84E-08 | 4.95E-07 | 8.213094 |
| FOXD1 | 1.50298 | 5.044259 | 5.763363 | 3.99E-08 | 5.13E-07 | 8.176779 |
| PET112 | -1.30148 | 7.2193 | -5.75519 | 4.15E-08 | 5.30E-07 | 8.137879 |
| SOSTDC1 | -1.61174 | 6.228815 | -5.75511 | 4.15E-08 | 5.30E-07 | 8.137501 |
| MSH2 | -1.426 | 5.555649 | -5.75271 | 4.20E-08 | 5.36E-07 | 8.126086 |
| ASAP1-IT2 | 1.1685 | 5.488885 | 5.750959 | 4.24E-08 | 5.39E-07 | 8.117785 |
| PCSK1 | -1.55951 | 7.409213 | -5.75044 | 4.25E-08 | 5.40E-07 | 8.115315 |
| FHOD3 | -1.08338 | 7.509005 | -5.74663 | 4.33E-08 | 5.50E-07 | 8.097234 |
| UBL7 | -1.31705 | 6.406775 | -5.74157 | 4.44E-08 | 5.62E-07 | 8.073201 |
| CASP7 | 1.042456 | 6.866841 | 5.741309 | 4.44E-08 | 5.63E-07 | 8.071954 |
| ELMO1 | -1.06434 | 6.813214 | -5.73435 | 4.60E-08 | 5.81E-07 | 8.038953 |
| AY940074 | 1.01415 | 6.383472 | 5.72335 | 4.85E-08 | 6.10E-07 | 7.98679 |
| PAQR6 | 1.179767 | 9.024337 | 5.712893 | 5.11E-08 | 6.37E-07 | 7.937284 |
| PSMD8 | -1.51235 | 7.233054 | -5.70688 | 5.26E-08 | 6.54E-07 | 7.908851 |
| CDK5 | -1.21483 | 7.816935 | -5.70595 | 5.29E-08 | 6.56E-07 | 7.904461 |
| AQP11 | -1.24725 | 6.024039 | -5.67809 | 6.06E-08 | 7.44E-07 | 7.772932 |
| ERC2-IT1 | 1.129202 | 7.935233 | 5.671949 | 6.24E-08 | 7.64E-07 | 7.744019 |
| EIF3C | 1.115083 | 5.23773 | 5.659837 | 6.62E-08 | 8.03E-07 | 7.687027 |
| LAMTOR2 | -1.04381 | 5.959954 | -5.63122 | 7.61E-08 | 9.09E-07 | 7.552713 |
| VAC14-AS1 | 1.238558 | 6.000549 | 5.62132 | 7.99E-08 | 9.48E-07 | 7.50633 |
| AP000347.2 | 1.077042 | 5.686756 | 5.616862 | 8.16E-08 | 9.68E-07 | 7.485467 |
| DMAP1 | -1.43283 | 6.265674 | -5.61427 | 8.26E-08 | 9.77E-07 | 7.473337 |
| C14orf79 | -1.1204 | 6.161149 | -5.61011 | 8.43E-08 | 9.95E-07 | 7.453875 |
| LOC285696 | 1.212105 | 5.212943 | 5.609201 | 8.47E-08 | 9.98E-07 | 7.449646 |
| ACTRT3 | -1.10006 | 4.804109 | -5.60675 | 8.57E-08 | 1.01E-06 | 7.438182 |
| EXOSC5 | -1.17651 | 5.888454 | -5.60354 | 8.70E-08 | 1.02E-06 | 7.423197 |
| CCT7 | -1.06355 | 8.814116 | -5.60255 | 8.75E-08 | 1.03E-06 | 7.418594 |
| ZNF488 | -1.29471 | 5.239669 | -5.58742 | 9.41E-08 | 1.10E-06 | 7.347972 |
| NAT6 | -1.1746 | 4.966723 | -5.54852 | 1.14E-07 | 1.30E-06 | 7.167086 |
| THEM6 | -1.24534 | 5.099698 | -5.54289 | 1.17E-07 | 1.34E-06 | 7.140966 |
| ESAM | 1.27948 | 4.77504 | 5.534722 | 1.21E-07 | 1.39E-06 | 7.103109 |
| LOC729870 | -1.14451 | 4.650987 | -5.52747 | 1.26E-07 | 1.43E-06 | 7.069515 |
| EPDR1 | -1.0702 | 9.484649 | -5.52256 | 1.29E-07 | 1.46E-06 | 7.046805 |
| SMIM10 | 1.043228 | 5.139812 | 5.522367 | 1.29E-07 | 1.46E-06 | 7.045925 |
| SCO1 | -1.21381 | 4.803281 | -5.52161 | 1.29E-07 | 1.46E-06 | 7.042424 |
| MIR34A | 1.12181 | 7.084963 | 5.49983 | 1.43E-07 | 1.60E-06 | 6.941847 |
| SPHKAP | -1.58644 | 8.666916 | -5.49517 | 1.47E-07 | 1.64E-06 | 6.920373 |
| YJEFN3 | -1.10638 | 7.202463 | -5.49439 | 1.47E-07 | 1.64E-06 | 6.916778 |
| ANKRD34C | -1.09126 | 7.206947 | -5.48893 | 1.51E-07 | 1.68E-06 | 6.891634 |
| PNMAL2 | -1.28996 | 7.887399 | -5.48791 | 1.52E-07 | 1.69E-06 | 6.8869 |
| LENG8 | 1.000524 | 7.093448 | 5.48539 | 1.54E-07 | 1.71E-06 | 6.875313 |
| TCIRG1 | 1.404021 | 5.16526 | 5.467381 | 1.67E-07 | 1.85E-06 | 6.792504 |
| RBP4 | -1.22631 | 6.585244 | -5.4621 | 1.72E-07 | 1.89E-06 | 6.76827 |
| TCAP | 1.051468 | 4.8865 | 5.453391 | 1.79E-07 | 1.96E-06 | 6.728304 |
| NPTX2 | -1.00083 | 8.223457 | -5.4393 | 1.91E-07 | 2.08E-06 | 6.663757 |
| NME7 | -1.00979 | 10.80939 | -5.41647 | 2.13E-07 | 2.30E-06 | 6.559437 |
| CHAF1B | -1.0805 | 5.05847 | -5.40766 | 2.22E-07 | 2.38E-06 | 6.519253 |
| MRGPRF | 1.151573 | 5.729601 | 5.407259 | 2.23E-07 | 2.39E-06 | 6.517408 |
| GJA4 | 1.066425 | 5.773195 | 5.397424 | 2.33E-07 | 2.49E-06 | 6.472604 |
| PODXL2 | -1.11879 | 5.972415 | -5.38817 | 2.44E-07 | 2.59E-06 | 6.430491 |
| PKD1P1 | 1.162049 | 5.615146 | 5.382074 | 2.51E-07 | 2.64E-06 | 6.402794 |
| RP4-758J24.5 | -1.10173 | 7.393322 | -5.36318 | 2.74E-07 | 2.87E-06 | 6.31708 |
| NLRC5 | 1.159953 | 6.778021 | 5.359055 | 2.80E-07 | 2.92E-06 | 6.298367 |
| AK021977 | 1.174795 | 7.963661 | 5.358587 | 2.80E-07 | 2.92E-06 | 6.296249 |
| FAM65C | 1.102396 | 4.791179 | 5.357511 | 2.82E-07 | 2.93E-06 | 6.291376 |
| LRRC4 | -1.21967 | 6.696544 | -5.35188 | 2.89E-07 | 3.00E-06 | 6.265872 |
| SYAP1 | -1.19596 | 6.208788 | -5.34504 | 2.99E-07 | 3.08E-06 | 6.234942 |
| ZNF217 | 1.001784 | 5.609206 | 5.34058 | 3.05E-07 | 3.13E-06 | 6.214781 |
| NEUROD6 | -1.19001 | 7.649444 | -5.32845 | 3.23E-07 | 3.31E-06 | 6.160001 |
| ACSF2 | 1.146983 | 5.614625 | 5.317926 | 3.39E-07 | 3.46E-06 | 6.112563 |
| RP3-327A19.5 | 1.052238 | 5.262109 | 5.317695 | 3.40E-07 | 3.46E-06 | 6.111519 |
| SLC39A10 | -1.2048 | 9.646505 | -5.31091 | 3.51E-07 | 3.56E-06 | 6.08096 |
| TTLL1 | -1.00926 | 6.574303 | -5.29875 | 3.71E-07 | 3.75E-06 | 6.026275 |
| SLC14A1 | 1.111787 | 7.586207 | 5.283475 | 3.99E-07 | 3.99E-06 | 5.957698 |
| EIF3G | -1.01139 | 8.453812 | -5.27841 | 4.08E-07 | 4.08E-06 | 5.934988 |
| UHRF1 | 1.174074 | 5.598142 | 5.251443 | 4.63E-07 | 4.54E-06 | 5.814345 |
| NDRG2 | 1.016199 | 9.343557 | 5.251069 | 4.64E-07 | 4.54E-06 | 5.812674 |
| NECAB1 | -1.14347 | 6.463563 | -5.24517 | 4.77E-07 | 4.66E-06 | 5.786334 |
| WDR46 | -1.28714 | 5.566282 | -5.23024 | 5.11E-07 | 4.95E-06 | 5.719785 |
| GIMAP7 | 1.248935 | 5.961031 | 5.230124 | 5.11E-07 | 4.95E-06 | 5.719282 |
| TDRD10 | 1.048742 | 5.966521 | 5.226098 | 5.21E-07 | 5.03E-06 | 5.701359 |
| CHI3L1 | 1.007343 | 7.915774 | 5.208009 | 5.66E-07 | 5.43E-06 | 5.620956 |
| ANKRD39 | -1.02117 | 6.232494 | -5.20535 | 5.73E-07 | 5.49E-06 | 5.60917 |
| NELL2 | -1.153 | 11.89357 | -5.19967 | 5.88E-07 | 5.62E-06 | 5.583941 |
| PEAR1 | 1.151018 | 5.889268 | 5.190996 | 6.12E-07 | 5.81E-06 | 5.545517 |
| SLC6A12 | 1.024129 | 6.787601 | 5.18286 | 6.36E-07 | 6.01E-06 | 5.509501 |
| RP11-65J3.14 | 1.088241 | 4.972008 | 5.18279 | 6.36E-07 | 6.01E-06 | 5.509193 |
| BC045789 | 1.180756 | 5.061598 | 5.178478 | 6.49E-07 | 6.12E-06 | 5.490121 |
| PCDHAC2 | -1.12726 | 3.618616 | -5.14741 | 7.48E-07 | 6.94E-06 | 5.353032 |
| PLK2 | -1.20151 | 9.197685 | -5.14272 | 7.64E-07 | 7.08E-06 | 5.332429 |
| LRRC75A | -1.08918 | 5.548683 | -5.14235 | 7.66E-07 | 7.08E-06 | 5.330774 |
| UBE2S | -1.1 | 5.041316 | -5.13717 | 7.84E-07 | 7.23E-06 | 5.30802 |
| OR7E47P | -1.05288 | 4.786027 | -5.13384 | 7.96E-07 | 7.33E-06 | 5.293362 |
| KDELC2 | 1.015184 | 5.920906 | 5.111395 | 8.82E-07 | 8.05E-06 | 5.194899 |
| NELL1 | -1.10934 | 6.605702 | -5.0917 | 9.65E-07 | 8.70E-06 | 5.108739 |
| P2RY14 | 1.295616 | 6.70844 | 5.075418 | 1.04E-06 | 9.28E-06 | 5.037716 |
| COG7 | -1.10039 | 5.309476 | -5.06973 | 1.07E-06 | 9.49E-06 | 5.012951 |
| ZNF222 | -1.20563 | 5.299015 | -5.03729 | 1.24E-06 | 1.08E-05 | 4.872035 |
| CORO1A | -1.2181 | 6.660542 | -5.03501 | 1.25E-06 | 1.09E-05 | 4.862155 |
| PRKAG1 | -1.02368 | 5.927322 | -5.01325 | 1.38E-06 | 1.19E-05 | 4.768018 |
| STAG3L3 | 1.147217 | 5.366982 | 4.986285 | 1.55E-06 | 1.32E-05 | 4.651776 |
| LOC100129973 | -1.23133 | 4.410879 | -4.98607 | 1.56E-06 | 1.32E-05 | 4.650871 |
| GOLT1A | -1.21398 | 3.618666 | -4.97906 | 1.61E-06 | 1.36E-05 | 4.620702 |
| MTFR1L | -1.03656 | 7.53982 | -4.96961 | 1.68E-06 | 1.41E-05 | 4.580126 |
| CLTC-IT1 | 1.105478 | 5.197181 | 4.957598 | 1.77E-06 | 1.48E-05 | 4.528628 |
| APLNR | 1.35072 | 6.57992 | 4.95235 | 1.81E-06 | 1.51E-05 | 4.506155 |
| FCRLB | -1.12969 | 5.841934 | -4.93309 | 1.97E-06 | 1.63E-05 | 4.423855 |
| SLC16A9 | 1.153476 | 7.00146 | 4.930752 | 1.99E-06 | 1.65E-05 | 4.413855 |
| TMEM59L | -1.10504 | 6.319098 | -4.92815 | 2.02E-06 | 1.66E-05 | 4.402754 |
| LOC100288310 | -1.13198 | 7.369564 | -4.92382 | 2.06E-06 | 1.69E-05 | 4.384305 |
| AP000265.1 | 1.101417 | 4.90361 | 4.916871 | 2.12E-06 | 1.74E-05 | 4.354695 |
| OCA2 | -1.01627 | 5.873673 | -4.90879 | 2.20E-06 | 1.80E-05 | 4.320314 |
| AOC3 | 1.189596 | 5.632742 | 4.883 | 2.46E-06 | 1.99E-05 | 4.210849 |
| RARRES2 | 1.14117 | 6.375398 | 4.872008 | 2.59E-06 | 2.07E-05 | 4.164326 |
| FLJ37786 | -1.15712 | 5.191088 | -4.85774 | 2.75E-06 | 2.20E-05 | 4.104053 |
| PP12719 | 1.0063 | 5.699894 | 4.85174 | 2.83E-06 | 2.25E-05 | 4.07875 |
| AP000253.1 | 1.074577 | 4.776476 | 4.849844 | 2.85E-06 | 2.27E-05 | 4.070758 |
| SERPINI1 | -1.01902 | 11.15554 | -4.84305 | 2.94E-06 | 2.33E-05 | 4.04216 |
| NEU1 | -1.01945 | 6.001943 | -4.842 | 2.95E-06 | 2.34E-05 | 4.037701 |
| LSR | 1.040827 | 4.292027 | 4.836255 | 3.03E-06 | 2.39E-05 | 4.013549 |
| SYT1 | -1.09668 | 11.73471 | -4.83586 | 3.03E-06 | 2.39E-05 | 4.011903 |
| RDH5 | 1.033339 | 6.276776 | 4.790788 | 3.70E-06 | 2.84E-05 | 3.823 |
| LOC102724611 | 1.072886 | 3.984427 | 4.774872 | 3.96E-06 | 3.02E-05 | 3.756618 |
| PDE6H | -1.0297 | 4.013847 | -4.75074 | 4.40E-06 | 3.32E-05 | 3.656266 |
| SCN3B | -1.06628 | 9.975144 | -4.73584 | 4.70E-06 | 3.53E-05 | 3.59451 |
| ZNF713 | 1.063026 | 4.934895 | 4.733261 | 4.75E-06 | 3.56E-05 | 3.583853 |
| DUSP4 | -1.10339 | 5.518617 | -4.71861 | 5.06E-06 | 3.77E-05 | 3.523306 |
| OSTF1 | -1.01251 | 6.239245 | -4.7181 | 5.07E-06 | 3.78E-05 | 3.521207 |
| KRTAP10-11 | 1.006651 | 5.167706 | 4.709125 | 5.27E-06 | 3.90E-05 | 3.484166 |
| AMZ2P1 | -1.05564 | 6.158224 | -4.70748 | 5.31E-06 | 3.93E-05 | 3.477379 |
| PSMC3 | -1.0253 | 6.143264 | -4.70173 | 5.44E-06 | 4.01E-05 | 3.453695 |
| RFWD3 | -1.01669 | 4.361748 | -4.68039 | 5.97E-06 | 4.35E-05 | 3.365989 |
| LYPD6B | -1.01028 | 4.476527 | -4.66753 | 6.30E-06 | 4.57E-05 | 3.313279 |
| IL13RA2 | -1.22025 | 5.001608 | -4.65923 | 6.53E-06 | 4.72E-05 | 3.279304 |
| LOC158434 | 1.054807 | 4.905398 | 4.65499 | 6.65E-06 | 4.79E-05 | 3.261992 |
| TMEM200A | -1.09499 | 7.261097 | -4.64304 | 7.00E-06 | 5.02E-05 | 3.213229 |
| NWD2 | -1.0432 | 6.632903 | -4.60424 | 8.26E-06 | 5.80E-05 | 3.055477 |
| ZMAT4 | -1.2024 | 7.111912 | -4.58349 | 9.02E-06 | 6.27E-05 | 2.971545 |
| HIGD1B | 1.516901 | 6.179053 | 4.553642 | 1.02E-05 | 7.02E-05 | 2.851341 |
| VSIG10L | -1.02773 | 5.189702 | -4.55064 | 1.04E-05 | 7.10E-05 | 2.839296 |
| RP11-421E14.2 | 1.006587 | 4.617294 | 4.520772 | 1.18E-05 | 7.95E-05 | 2.719651 |
| SUSD1 | -1.0917 | 5.815808 | -4.51308 | 1.21E-05 | 8.18E-05 | 2.688944 |
| PYCRL | -1.14101 | 4.559233 | -4.48102 | 1.39E-05 | 9.25E-05 | 2.561367 |
| PLA2G4A | -1.08497 | 4.946994 | -4.47938 | 1.40E-05 | 9.30E-05 | 2.554893 |
| TGFB1I1 | 1.350662 | 5.661954 | 4.448905 | 1.59E-05 | 0.000104 | 2.434319 |
| SERPINA3 | 1.388325 | 7.614233 | 4.429633 | 1.72E-05 | 0.000112 | 2.35841 |
| POR | -1.05602 | 6.067162 | -4.41063 | 1.86E-05 | 0.00012 | 2.283832 |
| PPARG | -1.04321 | 3.811204 | -4.41037 | 1.86E-05 | 0.00012 | 2.282808 |
| LRRC69 | 1.020737 | 5.511312 | 4.409568 | 1.87E-05 | 0.00012 | 2.279652 |
| RTBDN | -1.03104 | 5.013435 | -4.38043 | 2.11E-05 | 0.000133 | 2.165793 |
| LOC101929143 | 1.004874 | 5.141 | 4.377435 | 2.13E-05 | 0.000134 | 2.154109 |
| TYRP1 | -1.16183 | 5.340868 | -4.3721 | 2.18E-05 | 0.000137 | 2.133321 |
| TMEM151A | -1.02861 | 5.368355 | -4.32978 | 2.59E-05 | 0.000159 | 1.969271 |
| RP11-305O6.3 | 1.012959 | 5.500417 | 4.329678 | 2.59E-05 | 0.000159 | 1.968866 |
| ZNF165 | -1.17785 | 4.061544 | -4.32523 | 2.64E-05 | 0.000162 | 1.951679 |
| DYDC2 | -1.03419 | 4.194672 | -4.3178 | 2.72E-05 | 0.000166 | 1.923063 |
| HLA-DRB4 | 1.069382 | 3.701093 | 4.302558 | 2.89E-05 | 0.000176 | 1.864388 |
| SLC30A3 | -1.09204 | 6.740082 | -4.28208 | 3.14E-05 | 0.000189 | 1.785837 |
| SNCB | -1.0487 | 6.780565 | -4.24901 | 3.59E-05 | 0.000213 | 1.659652 |
| LOC100132891 | -1.05106 | 5.886953 | -4.18918 | 4.56E-05 | 0.000263 | 1.433294 |
| TAC1 | -1.19764 | 8.042635 | -4.04661 | 7.99E-05 | 0.00043 | 0.904365 |
| UBE2M | -1.11865 | 5.007668 | -4.01202 | 9.13E-05 | 0.000484 | 0.778322 |
| RGS1 | 1.162419 | 5.513637 | 3.992651 | 9.84E-05 | 0.000517 | 0.708096 |
| PVALB | -1.19058 | 6.194649 | -3.58688 | 0.000441 | 0.001932 | -0.69741 |
| RSPO2 | -1.02309 | 5.51148 | -3.58106 | 0.000451 | 0.001967 | -0.71664 |
| RPS4Y1 | -1.56707 | 7.6452 | -3.53054 | 0.000539 | 0.002297 | -0.88248 |
| FREM3 | -1.00408 | 4.754516 | -3.15538 | 0.001907 | 0.006949 | -2.04991 |

Supplementary Table 1. The results of DEGs in AD.
