# Supplementary material for: Exploring the pathogenesis and key genes associated of acute myocardial infarction complicated with Alzheimer’s disease
Source: Sci Rep. 2024 Jan 16;14:1449. doi: 10.1038/s41598-024-52094-4 (PMC10791667; doi:10.1038/s41598-024-52094-4)
Supplement: Supplementary file 2 — Supplementary Table 2. [file 41598_2024_52094_MOESM2_ESM.docx]

| id | logFC | AveExpr | t | P.Value | adj.P.Val | B |
| --- | --- | --- | --- | --- | --- | --- |
| NR4A2 | 2.952315 | 7.936055 | 12.21787 | 1.16E-21 | 2.51E-17 | 38.29484 |
| GABARAPL1 | 1.55827 | 8.444366 | 11.16689 | 2.26E-19 | 2.45E-15 | 33.25696 |
| NFKBIZ | 1.386215 | 10.62043 | 10.24877 | 2.38E-17 | 1.72E-13 | 28.796 |
| PDE4B | 1.312438 | 8.51378 | 9.919079 | 1.28E-16 | 6.91E-13 | 27.18658 |
| THBD | 1.948071 | 5.594177 | 9.862029 | 1.71E-16 | 7.39E-13 | 26.90791 |
| IRAK3 | 2.371639 | 6.531702 | 9.799945 | 2.34E-16 | 8.44E-13 | 26.60461 |
| ACSL1 | 2.205616 | 8.187668 | 9.710368 | 3.69E-16 | 1.01E-12 | 26.16698 |
| IL1R2 | 2.542094 | 7.502981 | 9.707301 | 3.75E-16 | 1.01E-12 | 26.152 |
| NFIL3 | 2.57321 | 8.53475 | 9.556068 | 8.09E-16 | 1.95E-12 | 25.41315 |
| MAP3K8 | 1.632965 | 7.666189 | 9.523942 | 9.53E-16 | 2.06E-12 | 25.25622 |
| ZFP36 | 1.955386 | 10.13921 | 9.41801 | 1.63E-15 | 3.11E-12 | 24.73884 |
| PELI1 | 1.929384 | 8.918911 | 9.407162 | 1.73E-15 | 3.11E-12 | 24.68587 |
| CLEC4E | 1.806929 | 7.288701 | 9.299646 | 2.98E-15 | 4.90E-12 | 24.16096 |
| BCL6 | 1.757633 | 7.465519 | 9.287533 | 3.17E-15 | 4.90E-12 | 24.10185 |
| S100A12 | 2.92834 | 7.768156 | 9.263646 | 3.58E-15 | 5.16E-12 | 23.98527 |
| NAMPT | 2.024186 | 9.634521 | 9.133694 | 6.92E-15 | 9.36E-12 | 23.35134 |
| SLC11A1 | 1.288174 | 6.660522 | 9.09805 | 8.29E-15 | 1.06E-11 | 23.17756 |
| IL1B | 2.72907 | 9.224539 | 8.993716 | 1.41E-14 | 1.69E-11 | 22.66917 |
| PPP1R15A | 2.030598 | 8.184995 | 8.608653 | 9.85E-14 | 1.12E-10 | 20.7976 |
| CCL20 | 2.752637 | 8.447883 | 8.575684 | 1.16E-13 | 1.26E-10 | 20.63779 |
| S100P | 2.342935 | 8.366427 | 8.463157 | 2.05E-13 | 2.11E-10 | 20.09295 |
| ITPRIP | 1.205901 | 6.922409 | 8.381266 | 3.09E-13 | 3.05E-10 | 19.6971 |
| HBEGF | 1.094994 | 5.737812 | 8.355257 | 3.53E-13 | 3.29E-10 | 19.5715 |
| FCN1 | 2.185565 | 7.100306 | 8.348674 | 3.64E-13 | 3.29E-10 | 19.53972 |
| JUN | 1.707281 | 9.517165 | 8.328078 | 4.04E-13 | 3.50E-10 | 19.44031 |
| VCAN | 2.475674 | 7.084572 | 8.316727 | 4.28E-13 | 3.56E-10 | 19.38554 |
| GADD45A | 1.456062 | 9.193059 | 8.299752 | 4.66E-13 | 3.74E-10 | 19.30366 |
| CLEC4D | 2.414411 | 6.095388 | 8.288021 | 4.94E-13 | 3.82E-10 | 19.24709 |
| FCER1G | 2.73015 | 8.489255 | 8.262544 | 5.61E-13 | 4.11E-10 | 19.12428 |
| FOSL2 | 1.023521 | 7.122425 | 8.255856 | 5.81E-13 | 4.11E-10 | 19.09205 |
| ADIPOR1 | 1.081447 | 9.195206 | 8.232931 | 6.51E-13 | 4.41E-10 | 18.98161 |
| MAFB | 2.019432 | 7.537164 | 8.184945 | 8.28E-13 | 5.43E-10 | 18.75062 |
| NR4A3 | 1.651149 | 5.646892 | 8.174542 | 8.72E-13 | 5.47E-10 | 18.70057 |
| NLRP3 | 1.650649 | 6.069121 | 8.171913 | 8.84E-13 | 5.47E-10 | 18.68793 |
| GLUL | 1.248286 | 7.795154 | 8.155372 | 9.60E-13 | 5.65E-10 | 18.60838 |
| LILRB2 | 2.103063 | 7.428578 | 8.154231 | 9.66E-13 | 5.65E-10 | 18.60289 |
| ITLN1 | 2.813349 | 6.765094 | 8.130618 | 1.09E-12 | 6.19E-10 | 18.48939 |
| JDP2 | 1.236876 | 6.001185 | 8.06985 | 1.47E-12 | 8.17E-10 | 18.19757 |
| NFKBIA | 1.784645 | 9.449659 | 8.006403 | 2.02E-12 | 1.09E-09 | 17.89334 |
| PLAUR | 1.846259 | 7.269852 | 7.989706 | 2.19E-12 | 1.16E-09 | 17.81335 |
| PMAIP1 | 1.626736 | 9.149289 | 7.98014 | 2.30E-12 | 1.19E-09 | 17.76755 |
| TLR2 | 2.104057 | 7.151334 | 7.908369 | 3.29E-12 | 1.63E-09 | 17.42422 |
| IL1RN | 1.381933 | 5.927639 | 7.906459 | 3.32E-12 | 1.63E-09 | 17.41509 |
| SERPINA1 | 1.544954 | 6.370433 | 7.86539 | 4.07E-12 | 1.93E-09 | 17.21894 |
| TREM1 | 2.608045 | 7.407035 | 7.864142 | 4.10E-12 | 1.93E-09 | 17.21298 |
| LYZ | 2.268833 | 9.108282 | 7.854053 | 4.31E-12 | 1.95E-09 | 17.16483 |
| TP53INP2 | 2.240001 | 7.876359 | 7.853199 | 4.33E-12 | 1.95E-09 | 17.16075 |
| CDA | 1.686091 | 6.422865 | 7.845425 | 4.50E-12 | 1.99E-09 | 17.12366 |
| QPCT | 2.147954 | 6.898733 | 7.818913 | 5.13E-12 | 2.22E-09 | 16.99722 |
| SLC7A7 | 2.045616 | 7.555962 | 7.803815 | 5.53E-12 | 2.35E-09 | 16.92526 |
| HAL | 1.226443 | 5.561364 | 7.754882 | 7.04E-12 | 2.93E-09 | 16.69225 |
| IER3 | 2.061858 | 10.92112 | 7.735846 | 7.74E-12 | 3.15E-09 | 16.6017 |
| S100A9 | 2.102607 | 9.288503 | 7.732785 | 7.85E-12 | 3.15E-09 | 16.58714 |
| CCL4 | 2.120241 | 9.064681 | 7.635811 | 1.27E-11 | 4.99E-09 | 16.12671 |
| CXCL2 | 1.974126 | 6.190973 | 7.627788 | 1.32E-11 | 5.10E-09 | 16.08868 |
| C5AR1 | 2.340348 | 9.10762 | 7.607761 | 1.46E-11 | 5.49E-09 | 15.99379 |
| CD83 | 2.250618 | 9.212644 | 7.605572 | 1.47E-11 | 5.49E-09 | 15.98343 |
| GAB2 | 1.417518 | 7.617555 | 7.586654 | 1.62E-11 | 5.84E-09 | 15.89386 |
| CSTA | 2.77912 | 8.381111 | 7.586222 | 1.62E-11 | 5.84E-09 | 15.89181 |
| CLEC7A | 1.536192 | 7.183855 | 7.559137 | 1.85E-11 | 6.56E-09 | 15.76368 |
| SNORD89 | 1.61384 | 6.426862 | 7.553496 | 1.90E-11 | 6.56E-09 | 15.73701 |
| CD55 | 1.046608 | 10.08213 | 7.552771 | 1.91E-11 | 6.56E-09 | 15.73358 |
| THBS1 | 1.553981 | 5.812115 | 7.497089 | 2.51E-11 | 8.49E-09 | 15.47058 |
| SULF2 | 1.567909 | 5.704011 | 7.447629 | 3.20E-11 | 1.05E-08 | 15.23741 |
| PHACTR1 | 1.888629 | 7.521293 | 7.445212 | 3.24E-11 | 1.05E-08 | 15.22603 |
| BST1 | 2.070488 | 6.594106 | 7.421287 | 3.64E-11 | 1.16E-08 | 15.1134 |
| TRIB1 | 1.040104 | 6.629852 | 7.417762 | 3.70E-11 | 1.16E-08 | 15.09681 |
| ETS2 | 1.441614 | 7.772222 | 7.361066 | 4.88E-11 | 1.51E-08 | 14.83034 |
| AIF1 | 1.596967 | 7.559929 | 7.210902 | 1.02E-10 | 3.01E-08 | 14.12742 |
| GADD45B | 1.365926 | 8.412338 | 7.202799 | 1.06E-10 | 3.09E-08 | 14.08961 |
| ALDH2 | 2.091358 | 6.849399 | 7.195426 | 1.09E-10 | 3.16E-08 | 14.05522 |
| ICAM1 | 1.388324 | 6.644421 | 7.188851 | 1.13E-10 | 3.22E-08 | 14.02456 |
| TULP2 | 1.772818 | 6.001902 | 7.148187 | 1.38E-10 | 3.87E-08 | 13.83513 |
| AQP9 | 2.374917 | 7.558256 | 7.134983 | 1.47E-10 | 4.07E-08 | 13.7737 |
| FOS | 2.076457 | 8.661939 | 7.107722 | 1.67E-10 | 4.59E-08 | 13.64696 |
| FCGR2A | 1.539222 | 7.692069 | 7.090933 | 1.82E-10 | 4.85E-08 | 13.56899 |
| CSF3R | 2.164968 | 7.252738 | 7.079406 | 1.92E-10 | 5.07E-08 | 13.51549 |
| CMTM2 | 1.969147 | 7.89621 | 7.074563 | 1.96E-10 | 5.10E-08 | 13.49302 |
| MAFF | 1.555547 | 8.166479 | 7.073 | 1.98E-10 | 5.10E-08 | 13.48577 |
| CPD | 1.105749 | 8.692247 | 7.059257 | 2.12E-10 | 5.39E-08 | 13.42204 |
| DYSF | 1.860946 | 7.08614 | 7.054642 | 2.16E-10 | 5.45E-08 | 13.40065 |
| FOSB | 1.70738 | 7.903259 | 7.009835 | 2.68E-10 | 6.68E-08 | 13.19318 |
| IL13RA1 | 1.481782 | 5.588701 | 6.915195 | 4.23E-10 | 1.03E-07 | 12.75642 |
| PYGL | 1.893873 | 7.211243 | 6.907459 | 4.39E-10 | 1.06E-07 | 12.72081 |
| VNN1 | 1.857097 | 5.478662 | 6.900896 | 4.53E-10 | 1.08E-07 | 12.69061 |
| CXCL16 | 1.878247 | 8.636878 | 6.890488 | 4.76E-10 | 1.12E-07 | 12.64273 |
| SKIL | 1.404856 | 6.904615 | 6.889074 | 4.79E-10 | 1.12E-07 | 12.63623 |
| TP53BP2 | 1.134883 | 8.506894 | 6.83289 | 6.27E-10 | 1.44E-07 | 12.37824 |
| GIMAP7 | -1.58989 | 10.21881 | -6.81859 | 6.72E-10 | 1.53E-07 | 12.3127 |
| PDZD8 | 1.072831 | 8.097144 | 6.81373 | 6.87E-10 | 1.55E-07 | 12.29042 |
| EFEMP1 | 1.48115 | 4.118601 | 6.81007 | 6.99E-10 | 1.56E-07 | 12.27366 |
| DDIT3 | 1.754275 | 7.957944 | 6.803494 | 7.22E-10 | 1.58E-07 | 12.24355 |
| CCRL2 | 1.741115 | 6.499497 | 6.801376 | 7.29E-10 | 1.58E-07 | 12.23385 |
| BCL2A1 | 1.846604 | 10.3888 | 6.786823 | 7.81E-10 | 1.68E-07 | 12.16725 |
| CH25H | 1.429837 | 4.761607 | 6.753534 | 9.16E-10 | 1.94E-07 | 12.01511 |
| LRG1 | 1.440023 | 6.016017 | 6.74848 | 9.38E-10 | 1.97E-07 | 11.99204 |
| MGP | 1.516344 | 5.950301 | 6.743143 | 9.62E-10 | 2.00E-07 | 11.96768 |
| MMP9 | 1.464139 | 6.688313 | 6.719945 | 1.07E-09 | 2.22E-07 | 11.86188 |
| GIMAP6 | -1.49421 | 9.336239 | -6.70907 | 1.13E-09 | 2.31E-07 | 11.8123 |
| SIRPA | 1.078639 | 6.589882 | 6.675193 | 1.33E-09 | 2.66E-07 | 11.65815 |
| CDKN1A | 1.630883 | 7.491806 | 6.658647 | 1.44E-09 | 2.80E-07 | 11.58295 |
| LILRA5 | 1.118406 | 5.289312 | 6.634816 | 1.61E-09 | 3.08E-07 | 11.47477 |
| ZNF137P | -1.42445 | 7.08115 | -6.6333 | 1.62E-09 | 3.08E-07 | 11.4679 |
| RGS1 | 1.269374 | 6.805258 | 6.625525 | 1.68E-09 | 3.16E-07 | 11.43263 |
| DUSP1 | 1.016175 | 8.099009 | 6.623332 | 1.70E-09 | 3.17E-07 | 11.42269 |
| PPIF | 1.231782 | 7.613046 | 6.600143 | 1.89E-09 | 3.51E-07 | 11.31763 |
| KLF4 | 1.371688 | 6.535638 | 6.570079 | 2.18E-09 | 4.01E-07 | 11.18164 |
| FPR1 | 1.610913 | 6.647189 | 6.55917 | 2.30E-09 | 4.18E-07 | 11.13235 |
| RBP7 | 1.728147 | 7.853605 | 6.551398 | 2.38E-09 | 4.30E-07 | 11.09726 |
| PILRA | 1.57364 | 7.620465 | 6.516682 | 2.81E-09 | 4.98E-07 | 10.94069 |
| PAQR8 | -1.13321 | 7.219048 | -6.48858 | 3.20E-09 | 5.60E-07 | 10.81419 |
| MME | 1.501072 | 6.50012 | 6.488342 | 3.21E-09 | 5.60E-07 | 10.81313 |
| ZEB2 | 1.195199 | 6.40069 | 6.48208 | 3.30E-09 | 5.72E-07 | 10.78497 |
| CCR2 | -1.53451 | 7.404833 | -6.44789 | 3.87E-09 | 6.66E-07 | 10.63144 |
| DOCK4 | 1.115387 | 5.605003 | 6.442509 | 3.97E-09 | 6.77E-07 | 10.60729 |
| FAM198B | 1.40656 | 5.22327 | 6.419295 | 4.43E-09 | 7.49E-07 | 10.50326 |
| CD33 | 1.533936 | 5.578064 | 6.39446 | 4.97E-09 | 8.22E-07 | 10.39213 |
| WDFY3 | 1.230341 | 5.634685 | 6.376756 | 5.40E-09 | 8.86E-07 | 10.31302 |
| DOCK5 | 1.043486 | 6.161851 | 6.368995 | 5.60E-09 | 9.12E-07 | 10.27837 |
| PTX3 | 2.604968 | 6.898403 | 6.365394 | 5.69E-09 | 9.20E-07 | 10.2623 |
| MGAM | 1.943331 | 7.633756 | 6.354437 | 5.99E-09 | 9.54E-07 | 10.21342 |
| TNFAIP2 | 1.225655 | 6.577886 | 6.331073 | 6.68E-09 | 1.06E-06 | 10.10931 |
| CTD-2541M15.1 | -1.37553 | 8.452161 | -6.28382 | 8.32E-09 | 1.30E-06 | 9.899228 |
| AC079305.10 | 1.947592 | 5.65066 | 6.268261 | 8.94E-09 | 1.38E-06 | 9.830213 |
| METRNL | 1.129834 | 6.520169 | 6.253188 | 9.58E-09 | 1.46E-06 | 9.763416 |
| SLC2A3 | 1.061003 | 10.06206 | 6.251266 | 9.67E-09 | 1.46E-06 | 9.754904 |
| SYTL3 | 1.048104 | 7.582614 | 6.23159 | 1.06E-08 | 1.58E-06 | 9.667824 |
| CSRNP1 | 1.154984 | 8.192152 | 6.222583 | 1.10E-08 | 1.62E-06 | 9.627998 |
| LILRA2 | 1.294384 | 6.049543 | 6.222515 | 1.10E-08 | 1.62E-06 | 9.6277 |
| GLT1D1 | 1.635183 | 6.205367 | 6.214015 | 1.15E-08 | 1.66E-06 | 9.59014 |
| RLF | 1.103213 | 9.404918 | 6.179947 | 1.34E-08 | 1.91E-06 | 9.439827 |
| MS4A6A | 1.46258 | 6.635068 | 6.167468 | 1.42E-08 | 2.01E-06 | 9.38486 |
| LINC00528 | 1.412258 | 7.277616 | 6.149499 | 1.55E-08 | 2.16E-06 | 9.305795 |
| CCR5 | -1.48597 | 8.371612 | -6.13781 | 1.63E-08 | 2.26E-06 | 9.254413 |
| RAB32 | 1.355108 | 5.812936 | 6.134907 | 1.65E-08 | 2.28E-06 | 9.24166 |
| FPR2 | 1.601883 | 5.977286 | 6.118755 | 1.78E-08 | 2.44E-06 | 9.170752 |
| CTD-2528L19.6 | -1.73202 | 5.638843 | -6.11608 | 1.80E-08 | 2.45E-06 | 9.159018 |
| TLR4 | 1.165107 | 5.907066 | 6.100616 | 1.93E-08 | 2.62E-06 | 9.091221 |
| SLC7A5 | 1.272911 | 9.112907 | 6.090487 | 2.03E-08 | 2.70E-06 | 9.046858 |
| SAMSN1 | 1.482108 | 7.683086 | 6.089797 | 2.03E-08 | 2.70E-06 | 9.043834 |
| PLBD1 | 2.009664 | 8.305542 | 6.072142 | 2.20E-08 | 2.91E-06 | 8.966588 |
| VNN3 | 1.111173 | 5.363707 | 6.059174 | 2.34E-08 | 3.07E-06 | 8.909916 |
| CD36 | 1.282896 | 6.011685 | 6.057324 | 2.36E-08 | 3.07E-06 | 8.901835 |
| SECTM1 | 1.268045 | 6.213411 | 6.018392 | 2.81E-08 | 3.65E-06 | 8.732045 |
| CD93 | 1.424234 | 6.83141 | 6.011968 | 2.90E-08 | 3.71E-06 | 8.704076 |
| P2RY13 | 1.923122 | 5.164386 | 5.999825 | 3.06E-08 | 3.90E-06 | 8.65125 |
| MBOAT2 | 1.061966 | 6.502258 | 5.947686 | 3.88E-08 | 4.87E-06 | 8.424977 |
| GNA15 | 1.052554 | 6.342377 | 5.940004 | 4.02E-08 | 5.00E-06 | 8.391717 |
| TSIX | -3.03561 | 6.855132 | -5.93193 | 4.17E-08 | 5.16E-06 | 8.35677 |
| PTAFR | 1.156686 | 6.110333 | 5.92699 | 4.26E-08 | 5.21E-06 | 8.335422 |
| CPVL | 1.852378 | 6.01584 | 5.925111 | 4.30E-08 | 5.23E-06 | 8.327298 |
| LPCAT2 | 1.226726 | 5.275108 | 5.915437 | 4.49E-08 | 5.37E-06 | 8.28549 |
| HCAR3 | 2.034079 | 7.664046 | 5.911433 | 4.57E-08 | 5.44E-06 | 8.268198 |
| MXD1 | 1.093944 | 8.903443 | 5.906306 | 4.68E-08 | 5.53E-06 | 8.24606 |
| CISH | -1.33416 | 7.857947 | -5.89866 | 4.84E-08 | 5.69E-06 | 8.213084 |
| ANXA3 | 1.849239 | 5.503245 | 5.896533 | 4.89E-08 | 5.69E-06 | 8.20389 |
| EREG | 1.702878 | 6.577381 | 5.890531 | 5.02E-08 | 5.82E-06 | 8.178009 |
| C9orf72 | 1.122019 | 6.91954 | 5.870953 | 5.49E-08 | 6.29E-06 | 8.093671 |
| ZNF420 | -1.15386 | 7.178174 | -5.86177 | 5.72E-08 | 6.52E-06 | 8.054179 |
| RNASE2 | 2.026149 | 6.524425 | 5.855529 | 5.88E-08 | 6.67E-06 | 8.027325 |
| GPR84 | 1.085058 | 4.276895 | 5.8311 | 6.56E-08 | 7.32E-06 | 7.922414 |
| SAT1 | 1.142112 | 11.09699 | 5.817633 | 6.97E-08 | 7.74E-06 | 7.864669 |
| CXCL1 | 1.97121 | 7.860518 | 5.809858 | 7.22E-08 | 7.93E-06 | 7.831362 |
| TMCC3 | 1.183847 | 6.531769 | 5.791506 | 7.84E-08 | 8.57E-06 | 7.752825 |
| CD163 | 1.576663 | 5.442316 | 5.770339 | 8.61E-08 | 9.37E-06 | 7.6624 |
| CD14 | 1.667404 | 6.73147 | 5.762615 | 8.92E-08 | 9.61E-06 | 7.629442 |
| XIST | -2.88871 | 5.626074 | -5.73294 | 1.02E-07 | 1.07E-05 | 7.503025 |
| MCEMP1 | 1.248949 | 7.026853 | 5.723475 | 1.06E-07 | 1.10E-05 | 7.462773 |
| RNF175 | 1.469592 | 7.982682 | 5.683386 | 1.27E-07 | 1.30E-05 | 7.292649 |
| CXCL8 | 1.405354 | 10.91466 | 5.681567 | 1.28E-07 | 1.31E-05 | 7.284946 |
| EPAS1 | 1.005064 | 5.682322 | 5.675442 | 1.31E-07 | 1.34E-05 | 7.259012 |
| GZMA | -1.06216 | 11.09716 | -5.67419 | 1.32E-07 | 1.34E-05 | 7.253694 |
| CD300LF | 1.727635 | 5.665539 | 5.661958 | 1.40E-07 | 1.40E-05 | 7.201968 |
| EMR2 | 1.114949 | 5.397933 | 5.648546 | 1.48E-07 | 1.48E-05 | 7.145295 |
| IFNGR1 | 1.023856 | 9.844998 | 5.634611 | 1.57E-07 | 1.56E-05 | 7.086483 |
| DMXL2 | 1.547331 | 7.242729 | 5.632227 | 1.59E-07 | 1.57E-05 | 7.076431 |
| LY96 | 1.056769 | 9.918041 | 5.605892 | 1.79E-07 | 1.73E-05 | 6.965518 |
| RP11-214K3.19 | -1.11256 | 8.054412 | -5.60132 | 1.82E-07 | 1.75E-05 | 6.946276 |
| FCGR3B | 1.657654 | 9.293966 | 5.573887 | 2.06E-07 | 1.95E-05 | 6.831084 |
| BCL10 | 1.021398 | 6.99377 | 5.566249 | 2.13E-07 | 2.00E-05 | 6.799061 |
| CRTAM | -1.53425 | 6.620847 | -5.54252 | 2.36E-07 | 2.20E-05 | 6.699707 |
| GPR97 | 1.177925 | 6.069168 | 5.540537 | 2.38E-07 | 2.21E-05 | 6.691431 |
| CHI3L1 | 1.264343 | 5.276083 | 5.485243 | 3.03E-07 | 2.76E-05 | 6.460851 |
| GCSAM | -1.07011 | 6.123603 | -5.48349 | 3.06E-07 | 2.77E-05 | 6.453572 |
| ARHGEF40 | 1.076359 | 7.335021 | 5.475934 | 3.16E-07 | 2.84E-05 | 6.422154 |
| ADM | 1.802934 | 8.486355 | 5.461112 | 3.37E-07 | 3.02E-05 | 6.360609 |
| ARL5B | 1.065332 | 7.387533 | 5.438213 | 3.72E-07 | 3.29E-05 | 6.2657 |
| RP11-96D1.11 | -1.0589 | 6.990549 | -5.42466 | 3.95E-07 | 3.46E-05 | 6.209629 |
| VNN2 | 1.331025 | 9.052973 | 5.417366 | 4.08E-07 | 3.53E-05 | 6.179486 |
| FOLR3 | 1.699067 | 6.128364 | 5.413016 | 4.15E-07 | 3.58E-05 | 6.161518 |
| HAUS3 | 1.206708 | 8.781939 | 5.409634 | 4.21E-07 | 3.62E-05 | 6.147555 |
| KCTD12 | 1.559075 | 7.793604 | 5.397868 | 4.44E-07 | 3.77E-05 | 6.099012 |
| DDX3Y | 1.490095 | 6.693725 | 5.389693 | 4.59E-07 | 3.84E-05 | 6.065319 |
| LOC153682 | -1.37474 | 6.75274 | -5.37599 | 4.88E-07 | 4.06E-05 | 6.008899 |
| TM6SF1 | 1.596148 | 5.793687 | 5.354948 | 5.34E-07 | 4.43E-05 | 5.922424 |
| SCML1 | 1.142842 | 6.193331 | 5.350209 | 5.45E-07 | 4.50E-05 | 5.902974 |
| CYP4F3 | 1.376102 | 6.04254 | 5.339179 | 5.71E-07 | 4.69E-05 | 5.857738 |
| RP6-99M1.2 | 1.599513 | 7.235234 | 5.333362 | 5.86E-07 | 4.75E-05 | 5.8339 |
| CEBPD | 1.197125 | 6.870976 | 5.294238 | 6.93E-07 | 5.56E-05 | 5.673965 |
| EOMES | -1.34424 | 9.653213 | -5.28959 | 7.07E-07 | 5.62E-05 | 5.655005 |
| GIMAP4 | -1.57993 | 9.58405 | -5.26747 | 7.77E-07 | 6.10E-05 | 5.564918 |
| LYN | 1.198664 | 8.839062 | 5.263766 | 7.90E-07 | 6.17E-05 | 5.54985 |
| WDR86-AS1 | -1.08797 | 6.039503 | -5.25792 | 8.10E-07 | 6.26E-05 | 5.526091 |
| RNF144B | 1.074231 | 6.697345 | 5.257164 | 8.12E-07 | 6.26E-05 | 5.523011 |
| SLC8A1-AS1 | 1.150674 | 5.529224 | 5.254168 | 8.23E-07 | 6.29E-05 | 5.510838 |
| MPP1 | 1.346687 | 7.206178 | 5.253698 | 8.24E-07 | 6.29E-05 | 5.508929 |
| GIMAP8 | -1.13313 | 6.794527 | -5.25335 | 8.26E-07 | 6.29E-05 | 5.507504 |
| TYROBP | 1.664738 | 9.433957 | 5.236642 | 8.86E-07 | 6.73E-05 | 5.439711 |
| RP11-443B7.1 | 1.026766 | 4.838478 | 5.210497 | 9.91E-07 | 7.48E-05 | 5.333848 |
| RAB31 | 1.084794 | 8.348299 | 5.207905 | 1.00E-06 | 7.52E-05 | 5.323371 |
| FURIN | 1.070463 | 9.004056 | 5.191971 | 1.07E-06 | 8.00E-05 | 5.259017 |
| G0S2 | 1.275791 | 10.29726 | 5.175765 | 1.15E-06 | 8.53E-05 | 5.193684 |
| TIMP2 | 1.058628 | 8.421523 | 5.174325 | 1.16E-06 | 8.53E-05 | 5.187885 |
| ZFP3 | -1.24468 | 5.765251 | -5.17372 | 1.16E-06 | 8.53E-05 | 5.18544 |
| S100A8 | 1.768333 | 10.81639 | 5.16662 | 1.19E-06 | 8.76E-05 | 5.156867 |
| C15orf48 | 1.732357 | 7.588728 | 5.16318 | 1.21E-06 | 8.86E-05 | 5.143027 |
| B3GALT2 | -1.52316 | 6.095988 | -5.12941 | 1.40E-06 | 0.0001 | 5.007463 |
| TNFAIP6 | 1.49277 | 6.462733 | 5.125134 | 1.42E-06 | 0.000102 | 4.990322 |
| MPEG1 | 1.842614 | 7.799304 | 5.11754 | 1.47E-06 | 0.000105 | 4.959918 |
| SULF1 | 1.33876 | 5.254576 | 5.111133 | 1.51E-06 | 0.000107 | 4.93429 |
| DUSP6 | 1.192619 | 7.891277 | 5.073529 | 1.77E-06 | 0.000121 | 4.784241 |
| RAD54B | -1.15775 | 7.113256 | -5.0677 | 1.81E-06 | 0.000124 | 4.761042 |
| KCNJ2 | 1.687087 | 8.237925 | 5.051764 | 1.94E-06 | 0.000131 | 4.697688 |
| DUSP4 | 1.112679 | 7.983248 | 5.045125 | 1.99E-06 | 0.000133 | 4.671333 |
| IDI2-AS1 | 1.012754 | 6.884554 | 5.02727 | 2.14E-06 | 0.000142 | 4.600546 |
| OGN | 1.1796 | 4.12624 | 5.026559 | 2.15E-06 | 0.000142 | 4.597732 |
| EDN1 | 1.139983 | 6.888924 | 5.024049 | 2.17E-06 | 0.000143 | 4.587793 |
| PTGDR | -1.00925 | 7.005617 | -5.00002 | 2.40E-06 | 0.000155 | 4.492823 |
| RELT | 1.028073 | 6.647214 | 4.980972 | 2.60E-06 | 0.000166 | 4.417696 |
| CLEC4A | 1.312078 | 7.046202 | 4.978823 | 2.62E-06 | 0.000167 | 4.409232 |
| FN1 | 1.017171 | 6.425621 | 4.972031 | 2.70E-06 | 0.00017 | 4.382502 |
| FAM49A | 1.134102 | 6.361498 | 4.963809 | 2.79E-06 | 0.000174 | 4.35017 |
| UTY | 1.33556 | 6.934594 | 4.952504 | 2.92E-06 | 0.000181 | 4.305765 |
| CLEC12A | 1.109034 | 5.560006 | 4.941937 | 3.06E-06 | 0.000188 | 4.264316 |
| CTB-31O20.2 | 1.486814 | 7.716819 | 4.936673 | 3.12E-06 | 0.00019 | 4.243689 |
| RP11-747H7.3 | -1.03878 | 8.121565 | -4.92929 | 3.22E-06 | 0.000194 | 4.21478 |
| LILRB3 | 1.040694 | 7.966904 | 4.913562 | 3.44E-06 | 0.000205 | 4.153281 |
| TNFAIP3 | 1.305685 | 10.82638 | 4.913076 | 3.44E-06 | 0.000205 | 4.151383 |
| IL23R | -1.10174 | 5.468734 | -4.89397 | 3.72E-06 | 0.00022 | 4.07683 |
| CEBPB | 1.415763 | 9.300176 | 4.888596 | 3.81E-06 | 0.000225 | 4.055905 |
| LINC00260 | -1.10959 | 6.560768 | -4.87126 | 4.09E-06 | 0.000237 | 3.988455 |
| GIMAP1 | -1.04203 | 7.355367 | -4.8688 | 4.13E-06 | 0.000238 | 3.9789 |
| ELOVL4 | -1.24154 | 7.773886 | -4.85481 | 4.37E-06 | 0.000249 | 3.924593 |
| HIST2H2BE | 1.231775 | 9.676212 | 4.84917 | 4.47E-06 | 0.000252 | 3.902747 |
| BRE-AS1 | 1.064996 | 5.098291 | 4.843478 | 4.58E-06 | 0.000256 | 3.880698 |
| TMEM176A | 1.187557 | 5.907551 | 4.839586 | 4.65E-06 | 0.000258 | 3.865628 |
| RASGEF1B | 1.096194 | 5.912715 | 4.836961 | 4.70E-06 | 0.00026 | 3.855474 |
| SIGLEC5 | 1.233305 | 6.047061 | 4.814719 | 5.15E-06 | 0.00028 | 3.769539 |
| ALDH1A1 | 1.425838 | 5.565833 | 4.813012 | 5.19E-06 | 0.00028 | 3.762953 |
| SLC15A3 | 1.113248 | 6.113394 | 4.812951 | 5.19E-06 | 0.00028 | 3.762719 |
| FGR | 1.821338 | 8.324391 | 4.805661 | 5.34E-06 | 0.000285 | 3.734612 |
| NRGN | 1.285091 | 7.448957 | 4.801221 | 5.44E-06 | 0.000289 | 3.71751 |
| DENND2D | -1.09734 | 8.691704 | -4.79849 | 5.50E-06 | 0.000291 | 3.706995 |
| SLC31A2 | 1.179006 | 7.995438 | 4.779777 | 5.94E-06 | 0.00031 | 3.635034 |
| LOC645984 | 1.351483 | 5.759009 | 4.743209 | 6.88E-06 | 0.000344 | 3.494927 |
| KIAA1598 | 1.118977 | 6.434448 | 4.727225 | 7.34E-06 | 0.000361 | 3.433899 |
| LOC731424 | 1.092091 | 5.926635 | 4.724322 | 7.43E-06 | 0.000365 | 3.422827 |
| GCA | 1.35466 | 7.898236 | 4.719616 | 7.57E-06 | 0.00037 | 3.404889 |
| RP11-140I16.3 | -1.30825 | 5.939438 | -4.71879 | 7.60E-06 | 0.000371 | 3.401761 |
| ZNF786 | -1.06337 | 7.042994 | -4.71208 | 7.81E-06 | 0.00038 | 3.376189 |
| AMPD2 | 1.063656 | 9.220397 | 4.709654 | 7.88E-06 | 0.000383 | 3.366959 |
| HMG20A | -1.06295 | 8.516057 | -4.70098 | 8.16E-06 | 0.000394 | 3.333983 |
| CXCL3 | 1.362645 | 6.557052 | 4.698953 | 8.23E-06 | 0.000396 | 3.326267 |
| RP11-373D23.2 | 1.276354 | 8.863936 | 4.693179 | 8.42E-06 | 0.000402 | 3.304338 |
| LGALS2 | 1.121542 | 6.641693 | 4.651281 | 9.96E-06 | 0.000469 | 3.145715 |
| VSIG1 | -1.11106 | 6.289854 | -4.62888 | 1.09E-05 | 0.000506 | 3.06128 |
| TNF | 1.24812 | 7.960339 | 4.619896 | 1.13E-05 | 0.00052 | 3.027492 |
| GVINP1 | -1.13434 | 8.3169 | -4.60205 | 1.21E-05 | 0.000549 | 2.960484 |
| FCGR1B | 1.249607 | 6.19757 | 4.601924 | 1.21E-05 | 0.000549 | 2.960027 |
| ZNF260 | -1.06409 | 8.512812 | -4.57171 | 1.37E-05 | 0.000608 | 2.846982 |
| MARCKS | 1.026299 | 8.98133 | 4.533993 | 1.59E-05 | 0.000688 | 2.706559 |
| FFAR2 | 1.121435 | 5.940505 | 4.501431 | 1.80E-05 | 0.00077 | 2.585939 |
| ARRDC4 | 1.3343 | 5.846341 | 4.497152 | 1.83E-05 | 0.000778 | 2.57013 |
| CFP | 1.342132 | 6.686641 | 4.488562 | 1.90E-05 | 0.000797 | 2.538424 |
| TCEB3-AS1 | -1.04808 | 6.906104 | -4.48103 | 1.95E-05 | 0.000815 | 2.510657 |
| AB488780 | -1.425 | 8.186092 | -4.46587 | 2.07E-05 | 0.000858 | 2.45488 |
| EIF1AY | 1.878602 | 6.35009 | 4.463878 | 2.09E-05 | 0.000863 | 2.447541 |
| TXLNGY | 1.849854 | 6.588421 | 4.453297 | 2.18E-05 | 0.000892 | 2.408683 |
| RPH3A | 1.02646 | 4.467388 | 4.436894 | 2.32E-05 | 0.000933 | 2.348568 |
| CSF1R | 1.082092 | 5.423404 | 4.42014 | 2.48E-05 | 0.000987 | 2.287321 |
| CTD-3025N20.3 | -1.10007 | 4.654458 | -4.39313 | 2.75E-05 | 0.001068 | 2.18891 |
| SGK1 | 1.139896 | 10.53675 | 4.386752 | 2.82E-05 | 0.001089 | 2.165727 |
| GIN1 | -1.16762 | 6.591422 | -4.37564 | 2.94E-05 | 0.001121 | 2.125409 |
| FTH1 | 1.092598 | 10.77532 | 4.367062 | 3.04E-05 | 0.001152 | 2.09431 |
| LEO1 | -1.06089 | 7.292612 | -4.36126 | 3.11E-05 | 0.001174 | 2.073323 |
| POP5 | -1.03417 | 9.197147 | -4.35875 | 3.14E-05 | 0.001181 | 2.06421 |
| ZNF331 | 1.057709 | 8.891511 | 4.357502 | 3.15E-05 | 0.001185 | 2.059711 |
| PLEK | 1.005314 | 8.724739 | 4.351618 | 3.22E-05 | 0.001206 | 2.038446 |
| HCK | 1.097549 | 6.963627 | 4.347955 | 3.27E-05 | 0.001221 | 2.025213 |
| RP11-722E23.2 | -1.0183 | 6.3742 | -4.34704 | 3.28E-05 | 0.001223 | 2.021918 |
| AMIGO2 | -1.25218 | 7.173222 | -4.34656 | 3.29E-05 | 0.001223 | 2.020178 |
| IRS2 | 1.097401 | 10.52027 | 4.33726 | 3.41E-05 | 0.001253 | 1.986631 |
| GZMB | 1.746151 | 7.01897 | 4.328807 | 3.52E-05 | 0.001292 | 1.95618 |
| PLA2G7 | 1.170702 | 4.284442 | 4.30918 | 3.79E-05 | 0.001376 | 1.885629 |
| PRSS35 | -1.25913 | 5.373192 | -4.30842 | 3.81E-05 | 0.001378 | 1.882887 |
| KLF10 | 1.152167 | 8.97255 | 4.30171 | 3.90E-05 | 0.001404 | 1.858838 |
| TNFAIP8L2 | -1.06428 | 8.007471 | -4.28436 | 4.17E-05 | 0.001485 | 1.796736 |
| LINC00959 | -1.0281 | 6.743245 | -4.27682 | 4.29E-05 | 0.001523 | 1.769786 |
| ZNF185 | 1.004778 | 7.183419 | 4.215771 | 5.41E-05 | 0.001835 | 1.552901 |
| EGR1 | 1.081433 | 9.776592 | 4.214483 | 5.43E-05 | 0.001839 | 1.548349 |
| EGR3 | 1.302892 | 7.81348 | 4.205111 | 5.63E-05 | 0.001896 | 1.515251 |
| TTTY15 | 1.328633 | 5.81936 | 4.196859 | 5.81E-05 | 0.001928 | 1.486151 |
| RGS2 | 1.18658 | 10.48251 | 4.194379 | 5.86E-05 | 0.001941 | 1.477412 |
| LIF | 1.013473 | 5.574514 | 4.169893 | 6.42E-05 | 0.002083 | 1.391336 |
| SPARCL1 | 1.247947 | 5.58445 | 4.168185 | 6.47E-05 | 0.002093 | 1.385346 |
| IER5 | 1.136013 | 8.788056 | 4.16701 | 6.49E-05 | 0.002099 | 1.381225 |
| USP9Y | 1.852057 | 6.134891 | 4.138191 | 7.23E-05 | 0.002303 | 1.280418 |
| CSF2RB | 1.190641 | 8.971872 | 4.1346 | 7.33E-05 | 0.002327 | 1.26789 |
| TLR8 | 1.152901 | 5.501845 | 4.111653 | 7.98E-05 | 0.002487 | 1.188025 |
| LOC101927069 | 1.259215 | 6.877557 | 4.110401 | 8.02E-05 | 0.002495 | 1.183675 |
| CTC-510F12.4 | 1.111439 | 7.534411 | 4.098943 | 8.37E-05 | 0.002585 | 1.143925 |
| CTA-29F11.1 | 1.174709 | 7.885983 | 4.096287 | 8.45E-05 | 0.002607 | 1.13472 |
| DNMBP | 1.097624 | 7.949329 | 4.081434 | 8.93E-05 | 0.00273 | 1.083329 |
| CD1D | 1.291182 | 6.186738 | 4.060098 | 9.66E-05 | 0.002905 | 1.009741 |
| NOG | -1.09645 | 7.131423 | -4.0592 | 9.69E-05 | 0.00291 | 1.006634 |
| FGL2 | 1.152095 | 7.848412 | 4.029737 | 0.000108 | 0.003172 | 0.905503 |
| A2M-AS1 | -1.16944 | 8.699878 | -4.02179 | 0.000111 | 0.003231 | 0.878308 |
| RTN1 | 1.031091 | 4.726045 | 4.009558 | 0.000116 | 0.003325 | 0.83653 |
| NCF2 | 1.181593 | 8.567334 | 4.003742 | 0.000119 | 0.003383 | 0.816698 |
| TMEM60 | -1.14071 | 7.717649 | -3.96811 | 0.000135 | 0.003758 | 0.695661 |
| PTGS2 | 1.434986 | 7.744198 | 3.962971 | 0.000138 | 0.003814 | 0.678255 |
| GZMK | -1.11849 | 10.57964 | -3.95262 | 0.000143 | 0.003906 | 0.643257 |
| RPS4Y1 | 2.459363 | 9.730992 | 3.946648 | 0.000146 | 0.003965 | 0.623115 |
| CREB5 | 1.060596 | 6.836759 | 3.942723 | 0.000148 | 0.004011 | 0.60988 |
| OR52K3P | 1.103848 | 3.805304 | 3.941173 | 0.000149 | 0.004022 | 0.604656 |
| KDM5D | 2.377003 | 8.098353 | 3.904012 | 0.00017 | 0.004507 | 0.479872 |
| PFKFB3 | 1.122373 | 11.53168 | 3.903442 | 0.000171 | 0.004507 | 0.477966 |
| LOC283357 | -1.20806 | 8.112336 | -3.88866 | 0.00018 | 0.004686 | 0.428577 |
| GEMIN5 | -1.06688 | 6.244414 | -3.87422 | 0.00019 | 0.004876 | 0.380476 |
| BTLA | -1.00913 | 7.899958 | -3.85802 | 0.000201 | 0.005117 | 0.326642 |
| ZFY | 1.079486 | 4.277797 | 3.855523 | 0.000203 | 0.005145 | 0.318355 |
| ZNF792 | -1.19412 | 6.236169 | -3.83423 | 0.000219 | 0.00546 | 0.247916 |
| MEST | -1.03895 | 5.272948 | -3.83028 | 0.000222 | 0.005519 | 0.234869 |
| CLEC1A | 1.022269 | 3.383349 | 3.798358 | 0.000248 | 0.006032 | 0.129858 |
| GJA1 | 1.483537 | 7.709951 | 3.79436 | 0.000252 | 0.006091 | 0.116754 |
| PKI55 | -1.00786 | 6.503707 | -3.75546 | 0.000289 | 0.006786 | -0.0102 |
| SRGN | 1.014535 | 10.4976 | 3.73748 | 0.000307 | 0.007111 | -0.06857 |
| LRRK2 | 1.350486 | 8.684755 | 3.718574 | 0.000328 | 0.007452 | -0.1297 |
| MOP-1 | 1.039443 | 4.780655 | 3.68792 | 0.000365 | 0.008135 | -0.22832 |
| MNDA | 1.190706 | 8.110909 | 3.671653 | 0.000386 | 0.008466 | -0.28041 |
| LOC100130357 | 1.108657 | 6.603887 | 3.664843 | 0.000395 | 0.008607 | -0.30216 |
| MS4A7 | 1.034893 | 5.722688 | 3.648136 | 0.000419 | 0.00899 | -0.35539 |
| TUBB2A | 1.332125 | 7.028538 | 3.634348 | 0.000439 | 0.009297 | -0.39918 |
| ZNF189 | -1.07013 | 6.88264 | -3.53493 | 0.000616 | 0.01199 | -0.71119 |
| LINC00094 | -1.02384 | 8.072624 | -3.51054 | 0.000668 | 0.012806 | -0.7867 |
| SLC22A4 | 1.08243 | 5.334862 | 3.420342 | 0.000902 | 0.016019 | -1.06242 |
| SERPINB2 | 1.162139 | 4.550771 | 3.380205 | 0.001029 | 0.017581 | -1.18329 |
| ZNF204P | -1.00851 | 5.681549 | -3.28876 | 0.001383 | 0.021941 | -1.45442 |
| PPBP | 1.104023 | 10.09968 | 3.07181 | 0.002731 | 0.03614 | -2.07356 |
| ASH1L-AS1 | 1.027575 | 7.054221 | 3.007197 | 0.003323 | 0.041497 | -2.25123 |

Supplementary Table 2. The results of DEGs in AMI.
