# Supplementary material for: Exploring the pathogenesis and key genes associated of acute myocardial infarction complicated with Alzheimer’s disease
Source: Sci Rep. 2024 Jan 16;14:1449. doi: 10.1038/s41598-024-52094-4 (PMC10791667; doi:10.1038/s41598-024-52094-4)
Supplement: Supplementary file 3 — Supplementary Table 3. [file 41598_2024_52094_MOESM3_ESM.docx]

| ONTOLOGY | ID | Description | GeneRatio | BgRatio | pvalue | p.adjust | qvalue | geneID | Count |
| --- | --- | --- | --- | --- | --- | --- | --- | --- | --- |
| BP | GO:0050727 | regulation of inflammatory response | 5月19日 | 386/18723 | 3.33E-05 | 0.033217 | 0.019915 | BCL6/NFKBIA/CEBPB/KLF4/FGR | 5 |
| BP | GO:0045185 | maintenance of protein location | 3月19日 | 94/18723 | 1.12E-04 | 0.034222 | 0.020518 | NFKBIA/GJA1/SRGN | 3 |
| BP | GO:0120162 | positive regulation of cold-induced thermogenesis | 3月19日 | 97/18723 | 1.23E-04 | 0.034222 | 0.020518 | CEBPB/GJA1/EPAS1 | 3 |
| BP | GO:0032496 | response to lipopolysaccharide | 4月19日 | 343/18723 | 3.45E-04 | 0.034222 | 0.020518 | NFKBIA/CEBPB/GJA1/CXCL1 | 4 |
| BP | GO:1903038 | negative regulation of leukocyte cell-cell adhesion | 3月19日 | 141/18723 | 3.71E-04 | 0.034222 | 0.020518 | BCL6/CEBPB/KLF4 | 3 |
| BP | GO:0001890 | placenta development | 3月19日 | 144/18723 | 3.94E-04 | 0.034222 | 0.020518 | CEBPB/GJA1/EPAS1 | 3 |
| BP | GO:0106106 | cold-induced thermogenesis | 3月19日 | 144/18723 | 3.94E-04 | 0.034222 | 0.020518 | CEBPB/GJA1/EPAS1 | 3 |
| BP | GO:0120161 | regulation of cold-induced thermogenesis | 3月19日 | 144/18723 | 3.94E-04 | 0.034222 | 0.020518 | CEBPB/GJA1/EPAS1 | 3 |
| BP | GO:0002237 | response to molecule of bacterial origin | 4月19日 | 363/18723 | 4.28E-04 | 0.034222 | 0.020518 | NFKBIA/CEBPB/GJA1/CXCL1 | 4 |
| BP | GO:0001701 | in utero embryonic development | 4月19日 | 367/18723 | 4.46E-04 | 0.034222 | 0.020518 | MAFF/CEBPB/KLF4/EPAS1 | 4 |
| BP | GO:0048333 | mesodermal cell differentiation | 2月19日 | 33/18723 | 5.06E-04 | 0.034222 | 0.020518 | GJA1/KLF4 | 2 |
| BP | GO:0051250 | negative regulation of lymphocyte activation | 3月19日 | 157/18723 | 5.08E-04 | 0.034222 | 0.020518 | BCL6/CEBPB/FGR | 3 |
| BP | GO:1990845 | adaptive thermogenesis | 3月19日 | 157/18723 | 5.08E-04 | 0.034222 | 0.020518 | CEBPB/GJA1/EPAS1 | 3 |
| BP | GO:0030099 | myeloid cell differentiation | 4月19日 | 381/18723 | 5.13E-04 | 0.034222 | 0.020518 | BCL6/NFKBIA/CEBPB/EPAS1 | 4 |
| BP | GO:0034405 | response to fluid shear stress | 2月19日 | 35/18723 | 5.69E-04 | 0.034222 | 0.020518 | GJA1/KLF4 | 2 |
| BP | GO:0045746 | negative regulation of Notch signaling pathway | 2月19日 | 36/18723 | 6.02E-04 | 0.034222 | 0.020518 | BCL6/NFKBIA | 2 |
| BP | GO:0031214 | biomineral tissue development | 3月19日 | 169/18723 | 6.29E-04 | 0.034222 | 0.020518 | CEBPB/SRGN/FGR | 3 |
| BP | GO:0110148 | biomineralization | 3月19日 | 171/18723 | 6.51E-04 | 0.034222 | 0.020518 | CEBPB/SRGN/FGR | 3 |
| BP | GO:0001503 | ossification | 4月19日 | 408/18723 | 6.64E-04 | 0.034222 | 0.020518 | VCAN/CEBPB/SRGN/FGR | 4 |
| BP | GO:0001659 | temperature homeostasis | 3月19日 | 174/18723 | 6.85E-04 | 0.034222 | 0.020518 | CEBPB/GJA1/EPAS1 | 3 |
| BP | GO:0002695 | negative regulation of leukocyte activation | 3月19日 | 187/18723 | 8.44E-04 | 0.04017 | 0.024084 | BCL6/CEBPB/FGR | 3 |
| BP | GO:0022408 | negative regulation of cell-cell adhesion | 3月19日 | 196/18723 | 9.67E-04 | 0.04393 | 0.026338 | BCL6/CEBPB/KLF4 | 3 |
| BP | GO:0043618 | regulation of transcription from RNA polymerase II promoter in response to stress | 2月19日 | 47/18723 | 1.03E-03 | 0.04458 | 0.026728 | CEBPB/EPAS1 | 2 |
| BP | GO:0071222 | cellular response to lipopolysaccharide | 3月19日 | 209/18723 | 1.16E-03 | 0.047172 | 0.028282 | NFKBIA/CEBPB/CXCL1 | 3 |
| BP | GO:0050866 | negative regulation of cell activation | 3月19日 | 210/18723 | 1.18E-03 | 0.047172 | 0.028282 | BCL6/CEBPB/FGR | 3 |
| BP | GO:0009612 | response to mechanical stimulus | 3月19日 | 216/18723 | 1.28E-03 | 0.048234 | 0.028918 | NFKBIA/GJA1/CHI3L1 | 3 |
| BP | GO:0043620 | regulation of DNA-templated transcription in response to stress | 2月19日 | 53/18723 | 1.30E-03 | 0.048234 | 0.028918 | CEBPB/EPAS1 | 2 |
| BP | GO:0071219 | cellular response to molecule of bacterial origin | 3月19日 | 221/18723 | 1.37E-03 | 0.048779 | 0.029245 | NFKBIA/CEBPB/CXCL1 | 3 |
| BP | GO:0043551 | regulation of phosphatidylinositol 3-kinase activity | 2月19日 | 59/18723 | 1.61E-03 | 0.055524 | 0.033289 | KLF4/FGR | 2 |
| BP | GO:1903670 | regulation of sprouting angiogenesis | 2月19日 | 60/18723 | 1.67E-03 | 0.055524 | 0.033289 | GLUL/KLF4 | 2 |
| BP | GO:0071216 | cellular response to biotic stimulus | 3月19日 | 246/18723 | 1.86E-03 | 0.059882 | 0.035902 | NFKBIA/CEBPB/CXCL1 | 3 |
| BP | GO:0032507 | maintenance of protein location in cell | 2月19日 | 65/18723 | 1.95E-03 | 0.060986 | 0.036564 | GJA1/SRGN | 2 |
| BP | GO:0043550 | regulation of lipid kinase activity | 2月19日 | 71/18723 | 2.33E-03 | 0.070236 | 0.04211 | KLF4/FGR | 2 |
| BP | GO:0001707 | mesoderm formation | 2月19日 | 72/18723 | 2.39E-03 | 0.070236 | 0.04211 | GJA1/KLF4 | 2 |
| BP | GO:0048332 | mesoderm morphogenesis | 2月19日 | 74/18723 | 2.52E-03 | 0.072013 | 0.043175 | GJA1/KLF4 | 2 |
| BP | GO:0031349 | positive regulation of defense response | 3月19日 | 278/18723 | 2.63E-03 | 0.073012 | 0.043774 | NFKBIA/CEBPB/GJA1 | 3 |
| BP | GO:1903532 | positive regulation of secretion by cell | 3月19日 | 282/18723 | 2.74E-03 | 0.073566 | 0.044106 | GLUL/GJA1/FGR | 3 |
| BP | GO:0032024 | positive regulation of insulin secretion | 2月19日 | 78/18723 | 2.80E-03 | 0.073566 | 0.044106 | GLUL/GJA1 | 2 |
| BP | GO:0009791 | post-embryonic development | 2月19日 | 80/18723 | 2.94E-03 | 0.075336 | 0.045167 | CSRNP1/KLF4 | 2 |
| BP | GO:0001892 | embryonic placenta development | 2月19日 | 82/18723 | 3.09E-03 | 0.077102 | 0.046226 | CEBPB/EPAS1 | 2 |
| BP | GO:0007162 | negative regulation of cell adhesion | 3月19日 | 303/18723 | 3.36E-03 | 0.081759 | 0.049018 | BCL6/CEBPB/KLF4 | 3 |
| BP | GO:0051047 | positive regulation of secretion | 3月19日 | 310/18723 | 3.58E-03 | 0.085109 | 0.051027 | GLUL/GJA1/FGR | 3 |
| BP | GO:0032088 | negative regulation of NF-kappaB transcription factor activity | 2月19日 | 93/18723 | 3.95E-03 | 0.089787 | 0.053831 | NFKBIA/KLF4 | 2 |
| BP | GO:0008593 | regulation of Notch signaling pathway | 2月19日 | 95/18723 | 4.12E-03 | 0.089787 | 0.053831 | BCL6/NFKBIA | 2 |
| BP | GO:0050764 | regulation of phagocytosis | 2月19日 | 95/18723 | 4.12E-03 | 0.089787 | 0.053831 | RAB31/FGR | 2 |
| BP | GO:0051235 | maintenance of location | 3月19日 | 327/18723 | 4.16E-03 | 0.089787 | 0.053831 | NFKBIA/GJA1/SRGN | 3 |
| BP | GO:0070167 | regulation of biomineral tissue development | 2月19日 | 97/18723 | 4.29E-03 | 0.089787 | 0.053831 | CEBPB/SRGN | 2 |
| BP | GO:0110149 | regulation of biomineralization | 2月19日 | 99/18723 | 4.46E-03 | 0.089787 | 0.053831 | CEBPB/SRGN | 2 |
| BP | GO:1903037 | regulation of leukocyte cell-cell adhesion | 3月19日 | 336/18723 | 4.48E-03 | 0.089787 | 0.053831 | BCL6/CEBPB/KLF4 | 3 |
| BP | GO:0045765 | regulation of angiogenesis | 3月19日 | 342/18723 | 4.71E-03 | 0.089787 | 0.053831 | GLUL/KLF4/CHI3L1 | 3 |
| BP | GO:0032677 | regulation of interleukin-8 production | 2月19日 | 102/18723 | 4.73E-03 | 0.089787 | 0.053831 | KLF4/CHI3L1 | 2 |
| BP | GO:0032637 | interleukin-8 production | 2月19日 | 103/18723 | 4.82E-03 | 0.089787 | 0.053831 | KLF4/CHI3L1 | 2 |
| BP | GO:0090277 | positive regulation of peptide hormone secretion | 2月19日 | 103/18723 | 4.82E-03 | 0.089787 | 0.053831 | GLUL/GJA1 | 2 |
| BP | GO:1901342 | regulation of vasculature development | 3月19日 | 348/18723 | 4.95E-03 | 0.089787 | 0.053831 | GLUL/KLF4/CHI3L1 | 3 |
| BP | GO:0002793 | positive regulation of peptide secretion | 2月19日 | 106/18723 | 5.10E-03 | 0.089787 | 0.053831 | GLUL/GJA1 | 2 |
| BP | GO:2000278 | regulation of DNA biosynthetic process | 2月19日 | 106/18723 | 5.10E-03 | 0.089787 | 0.053831 | GJA1/KLF4 | 2 |
| BP | GO:0032526 | response to retinoic acid | 2月19日 | 107/18723 | 5.19E-03 | 0.089787 | 0.053831 | GJA1/KLF4 | 2 |
| BP | GO:0001818 | negative regulation of cytokine production | 3月19日 | 357/18723 | 5.31E-03 | 0.089787 | 0.053831 | BCL6/SRGN/KLF4 | 3 |
| BP | GO:0051052 | regulation of DNA metabolic process | 3月19日 | 359/18723 | 5.39E-03 | 0.089787 | 0.053831 | BCL6/GJA1/KLF4 | 3 |
| BP | GO:1901653 | cellular response to peptide | 3月19日 | 359/18723 | 5.39E-03 | 0.089787 | 0.053831 | GJA1/RAB31/KLF4 | 3 |
| BP | GO:0014066 | regulation of phosphatidylinositol 3-kinase signaling | 2月19日 | 111/18723 | 5.58E-03 | 0.091329 | 0.054756 | KLF4/FGR | 2 |
| BP | GO:1903706 | regulation of hemopoiesis | 3月19日 | 367/18723 | 5.73E-03 | 0.09155 | 0.054888 | BCL6/NFKBIA/CEBPB | 3 |
| BP | GO:0071347 | cellular response to interleukin-1 | 2月19日 | 113/18723 | 5.77E-03 | 0.09155 | 0.054888 | CEBPB/CHI3L1 | 2 |
| BP | GO:0007159 | leukocyte cell-cell adhesion | 3月19日 | 371/18723 | 5.91E-03 | 0.092215 | 0.055287 | BCL6/CEBPB/KLF4 | 3 |
| BP | GO:0051101 | regulation of DNA binding | 2月19日 | 118/18723 | 6.28E-03 | 0.096504 | 0.057859 | NFKBIA/KLF4 | 2 |
| BP | GO:0030282 | bone mineralization | 2月19日 | 119/18723 | 6.38E-03 | 0.096608 | 0.057921 | SRGN/FGR | 2 |
| BP | GO:0030218 | erythrocyte differentiation | 2月19日 | 120/18723 | 6.49E-03 | 0.096721 | 0.057989 | BCL6/EPAS1 | 2 |
| BP | GO:0001704 | formation of primary germ layer | 2月19日 | 121/18723 | 6.59E-03 | 0.096842 | 0.058061 | GJA1/KLF4 | 2 |
| BP | GO:0050868 | negative regulation of T cell activation | 2月19日 | 122/18723 | 6.70E-03 | 0.09697 | 0.058138 | BCL6/CEBPB | 2 |
| BP | GO:0051053 | negative regulation of DNA metabolic process | 2月19日 | 125/18723 | 7.02E-03 | 0.097727 | 0.058592 | BCL6/GJA1 | 2 |
| BP | GO:0007498 | mesoderm development | 2月19日 | 129/18723 | 7.46E-03 | 0.097727 | 0.058592 | GJA1/KLF4 | 2 |
| BP | GO:0034101 | erythrocyte homeostasis | 2月19日 | 129/18723 | 7.46E-03 | 0.097727 | 0.058592 | BCL6/EPAS1 | 2 |
| BP | GO:0046887 | positive regulation of hormone secretion | 2月19日 | 133/18723 | 7.91E-03 | 0.097727 | 0.058592 | GLUL/GJA1 | 2 |
| BP | GO:0032102 | negative regulation of response to external stimulus | 3月19日 | 420/18723 | 8.31E-03 | 0.097727 | 0.058592 | GJA1/KLF4/FGR | 3 |
| BP | GO:0050714 | positive regulation of protein secretion | 2月19日 | 137/18723 | 8.38E-03 | 0.097727 | 0.058592 | GLUL/GJA1 | 2 |
| BP | GO:0048608 | reproductive structure development | 3月19日 | 424/18723 | 8.53E-03 | 0.097727 | 0.058592 | CEBPB/GJA1/EPAS1 | 3 |
| BP | GO:0061458 | reproductive system development | 3月19日 | 427/18723 | 8.70E-03 | 0.097727 | 0.058592 | CEBPB/GJA1/EPAS1 | 3 |
| BP | GO:0050729 | positive regulation of inflammatory response | 2月19日 | 142/18723 | 8.98E-03 | 0.097727 | 0.058592 | NFKBIA/CEBPB | 2 |
| BP | GO:0002683 | negative regulation of immune system process | 3月19日 | 434/18723 | 9.09E-03 | 0.097727 | 0.058592 | BCL6/CEBPB/FGR | 3 |
| BP | GO:0038061 | NIK/NF-kappaB signaling | 2月19日 | 143/18723 | 9.10E-03 | 0.097727 | 0.058592 | NFKBIA/CHI3L1 | 2 |
| BP | GO:0070555 | response to interleukin-1 | 2月19日 | 143/18723 | 9.10E-03 | 0.097727 | 0.058592 | CEBPB/CHI3L1 | 2 |
| BP | GO:0014065 | phosphatidylinositol 3-kinase signaling | 2月19日 | 144/18723 | 9.22E-03 | 0.097727 | 0.058592 | KLF4/FGR | 2 |
| BP | GO:0050673 | epithelial cell proliferation | 3月19日 | 437/18723 | 9.27E-03 | 0.097727 | 0.058592 | GLUL/CEBPB/GJA1 | 3 |
| BP | GO:0022407 | regulation of cell-cell adhesion | 3月19日 | 448/18723 | 9.92E-03 | 0.097727 | 0.058592 | BCL6/CEBPB/KLF4 | 3 |
| BP | GO:2000434 | regulation of protein neddylation | 1月19日 | 10/18723 | 1.01E-02 | 0.097727 | 0.058592 | EPAS1 | 1 |
| BP | GO:0030856 | regulation of epithelial cell differentiation | 2月19日 | 154/18723 | 1.05E-02 | 0.097727 | 0.058592 | MAFF/CEBPB | 2 |
| BP | GO:0002262 | myeloid cell homeostasis | 2月19日 | 157/18723 | 1.09E-02 | 0.097727 | 0.058592 | BCL6/EPAS1 | 2 |
| BP | GO:0001819 | positive regulation of cytokine production | 3月19日 | 467/18723 | 1.11E-02 | 0.097727 | 0.058592 | CEBPB/FGR/CHI3L1 | 3 |
| BP | GO:0002357 | defense response to tumor cell | 1月19日 | 11/18723 | 1.11E-02 | 0.097727 | 0.058592 | KLF4 | 1 |
| BP | GO:0003093 | regulation of glomerular filtration | 1月19日 | 11/18723 | 1.11E-02 | 0.097727 | 0.058592 | GJA1 | 1 |
| BP | GO:0032276 | regulation of gonadotropin secretion | 1月19日 | 11/18723 | 1.11E-02 | 0.097727 | 0.058592 | GJA1 | 1 |
| BP | GO:0034115 | negative regulation of heterotypic cell-cell adhesion | 1月19日 | 11/18723 | 1.11E-02 | 0.097727 | 0.058592 | KLF4 | 1 |
| BP | GO:0043619 | regulation of transcription from RNA polymerase II promoter in response to oxidative stress | 1月19日 | 11/18723 | 1.11E-02 | 0.097727 | 0.058592 | EPAS1 | 1 |
| BP | GO:0045628 | regulation of T-helper 2 cell differentiation | 1月19日 | 11/18723 | 1.11E-02 | 0.097727 | 0.058592 | BCL6 | 1 |
| BP | GO:0070417 | cellular response to cold | 1月19日 | 11/18723 | 1.11E-02 | 0.097727 | 0.058592 | NFKBIA | 1 |
| BP | GO:0070587 | regulation of cell-cell adhesion involved in gastrulation | 1月19日 | 11/18723 | 1.11E-02 | 0.097727 | 0.058592 | KLF4 | 1 |
| BP | GO:1990440 | positive regulation of transcription from RNA polymerase II promoter in response to endoplasmic reticulum stress | 1月19日 | 11/18723 | 1.11E-02 | 0.097727 | 0.058592 | CEBPB | 1 |
| BP | GO:0001667 | ameboidal-type cell migration | 3月19日 | 475/18723 | 1.16E-02 | 0.097727 | 0.058592 | GLUL/GJA1/KLF4 | 3 |
| BP | GO:0050796 | regulation of insulin secretion | 2月19日 | 165/18723 | 1.20E-02 | 0.097727 | 0.058592 | GLUL/GJA1 | 2 |
| BP | GO:0002903 | negative regulation of B cell apoptotic process | 1月19日 | 12/18723 | 1.21E-02 | 0.097727 | 0.058592 | BCL6 | 1 |
| BP | GO:0043129 | surfactant homeostasis | 1月19日 | 12/18723 | 1.21E-02 | 0.097727 | 0.058592 | EPAS1 | 1 |
| BP | GO:0043380 | regulation of memory T cell differentiation | 1月19日 | 12/18723 | 1.21E-02 | 0.097727 | 0.058592 | BCL6 | 1 |
| BP | GO:0070586 | cell-cell adhesion involved in gastrulation | 1月19日 | 12/18723 | 1.21E-02 | 0.097727 | 0.058592 | KLF4 | 1 |
| BP | GO:0071107 | response to parathyroid hormone | 1月19日 | 12/18723 | 1.21E-02 | 0.097727 | 0.058592 | GJA1 | 1 |
| BP | GO:1903034 | regulation of response to wounding | 2月19日 | 167/18723 | 1.22E-02 | 0.097727 | 0.058592 | GJA1/KLF4 | 2 |
| BP | GO:0007219 | Notch signaling pathway | 2月19日 | 172/18723 | 1.29E-02 | 0.097727 | 0.058592 | BCL6/NFKBIA | 2 |
| BP | GO:0002638 | negative regulation of immunoglobulin production | 1月19日 | 13/18723 | 1.31E-02 | 0.097727 | 0.058592 | BCL6 | 1 |
| BP | GO:0006000 | fructose metabolic process | 1月19日 | 13/18723 | 1.31E-02 | 0.097727 | 0.058592 | PFKFB3 | 1 |
| BP | GO:0007567 | parturition | 1月19日 | 13/18723 | 1.31E-02 | 0.097727 | 0.058592 | MAFF | 1 |
| BP | GO:0010745 | negative regulation of macrophage derived foam cell differentiation | 1月19日 | 13/18723 | 1.31E-02 | 0.097727 | 0.058592 | NFKBIA | 1 |
| BP | GO:0014745 | negative regulation of muscle adaptation | 1月19日 | 13/18723 | 1.31E-02 | 0.097727 | 0.058592 | KLF4 | 1 |
| BP | GO:0033262 | regulation of nuclear cell cycle DNA replication | 1月19日 | 13/18723 | 1.31E-02 | 0.097727 | 0.058592 | BCL6 | 1 |
| BP | GO:0042541 | hemoglobin biosynthetic process | 1月19日 | 13/18723 | 1.31E-02 | 0.097727 | 0.058592 | KLF4 | 1 |
| BP | GO:0043379 | memory T cell differentiation | 1月19日 | 13/18723 | 1.31E-02 | 0.097727 | 0.058592 | BCL6 | 1 |
| BP | GO:0060100 | positive regulation of phagocytosis, engulfment | 1月19日 | 13/18723 | 1.31E-02 | 0.097727 | 0.058592 | RAB31 | 1 |
| BP | GO:1904995 | negative regulation of leukocyte adhesion to vascular endothelial cell | 1月19日 | 13/18723 | 1.31E-02 | 0.097727 | 0.058592 | KLF4 | 1 |
| BP | GO:1905155 | positive regulation of membrane invagination | 1月19日 | 13/18723 | 1.31E-02 | 0.097727 | 0.058592 | RAB31 | 1 |
| BP | GO:2001198 | regulation of dendritic cell differentiation | 1月19日 | 13/18723 | 1.31E-02 | 0.097727 | 0.058592 | CEBPB | 1 |
| BP | GO:0048771 | tissue remodeling | 2月19日 | 175/18723 | 1.34E-02 | 0.097727 | 0.058592 | GJA1/EPAS1 | 2 |
| BP | GO:0050728 | negative regulation of inflammatory response | 2月19日 | 176/18723 | 1.35E-02 | 0.097727 | 0.058592 | KLF4/FGR | 2 |
| BP | GO:0030324 | lung development | 2月19日 | 177/18723 | 1.37E-02 | 0.097727 | 0.058592 | EPAS1/CHI3L1 | 2 |
| BP | GO:0048015 | phosphatidylinositol-mediated signaling | 2月19日 | 178/18723 | 1.38E-02 | 0.097727 | 0.058592 | KLF4/FGR | 2 |
| BP | GO:0048660 | regulation of smooth muscle cell proliferation | 2月19日 | 180/18723 | 1.41E-02 | 0.097727 | 0.058592 | GJA1/KLF4 | 2 |
| BP | GO:0071897 | DNA biosynthetic process | 2月19日 | 180/18723 | 1.41E-02 | 0.097727 | 0.058592 | GJA1/KLF4 | 2 |
| BP | GO:0002467 | germinal center formation | 1月19日 | 14/18723 | 1.41E-02 | 0.097727 | 0.058592 | BCL6 | 1 |
| BP | GO:0002829 | negative regulation of type 2 immune response | 1月19日 | 14/18723 | 1.41E-02 | 0.097727 | 0.058592 | BCL6 | 1 |
| BP | GO:0033008 | positive regulation of mast cell activation involved in immune response | 1月19日 | 14/18723 | 1.41E-02 | 0.097727 | 0.058592 | FGR | 1 |
| BP | GO:0034616 | response to laminar fluid shear stress | 1月19日 | 14/18723 | 1.41E-02 | 0.097727 | 0.058592 | KLF4 | 1 |
| BP | GO:0035437 | maintenance of protein localization in endoplasmic reticulum | 1月19日 | 14/18723 | 1.41E-02 | 0.097727 | 0.058592 | GJA1 | 1 |
| BP | GO:0035672 | oligopeptide transmembrane transport | 1月19日 | 14/18723 | 1.41E-02 | 0.097727 | 0.058592 | GJA1 | 1 |
| BP | GO:0042415 | norepinephrine metabolic process | 1月19日 | 14/18723 | 1.41E-02 | 0.097727 | 0.058592 | EPAS1 | 1 |
| BP | GO:0043306 | positive regulation of mast cell degranulation | 1月19日 | 14/18723 | 1.41E-02 | 0.097727 | 0.058592 | FGR | 1 |
| BP | GO:0046348 | amino sugar catabolic process | 1月19日 | 14/18723 | 1.41E-02 | 0.097727 | 0.058592 | CHI3L1 | 1 |
| BP | GO:0048875 | chemical homeostasis within a tissue | 1月19日 | 14/18723 | 1.41E-02 | 0.097727 | 0.058592 | EPAS1 | 1 |
| BP | GO:0090715 | immunological memory formation process | 1月19日 | 14/18723 | 1.41E-02 | 0.097727 | 0.058592 | BCL6 | 1 |
| BP | GO:1902969 | mitotic DNA replication | 1月19日 | 14/18723 | 1.41E-02 | 0.097727 | 0.058592 | BCL6 | 1 |
| BP | GO:0030323 | respiratory tube development | 2月19日 | 181/18723 | 1.43E-02 | 0.097727 | 0.058592 | EPAS1/CHI3L1 | 2 |
| BP | GO:0045766 | positive regulation of angiogenesis | 2月19日 | 181/18723 | 1.43E-02 | 0.097727 | 0.058592 | KLF4/CHI3L1 | 2 |
| BP | GO:1904018 | positive regulation of vasculature development | 2月19日 | 181/18723 | 1.43E-02 | 0.097727 | 0.058592 | KLF4/CHI3L1 | 2 |
| BP | GO:0048017 | inositol lipid-mediated signaling | 2月19日 | 182/18723 | 1.44E-02 | 0.097727 | 0.058592 | KLF4/FGR | 2 |
| BP | GO:0048659 | smooth muscle cell proliferation | 2月19日 | 184/18723 | 1.47E-02 | 0.097727 | 0.058592 | GJA1/KLF4 | 2 |
| BP | GO:0002040 | sprouting angiogenesis | 2月19日 | 185/18723 | 1.49E-02 | 0.097727 | 0.058592 | GLUL/KLF4 | 2 |
| BP | GO:0007369 | gastrulation | 2月19日 | 185/18723 | 1.49E-02 | 0.097727 | 0.058592 | GJA1/KLF4 | 2 |
| BP | GO:0043433 | negative regulation of DNA-binding transcription factor activity | 2月19日 | 185/18723 | 1.49E-02 | 0.097727 | 0.058592 | NFKBIA/KLF4 | 2 |
| BP | GO:0051896 | regulation of protein kinase B signaling | 2月19日 | 185/18723 | 1.49E-02 | 0.097727 | 0.058592 | KLF4/CHI3L1 | 2 |
| BP | GO:0042994 | cytoplasmic sequestering of transcription factor | 1月19日 | 15/18723 | 1.51E-02 | 0.097727 | 0.058592 | NFKBIA | 1 |
| BP | GO:0045064 | T-helper 2 cell differentiation | 1月19日 | 15/18723 | 1.51E-02 | 0.097727 | 0.058592 | BCL6 | 1 |
| BP | GO:0048569 | post-embryonic animal organ development | 1月19日 | 15/18723 | 1.51E-02 | 0.097727 | 0.058592 | KLF4 | 1 |
| BP | GO:0060099 | regulation of phagocytosis, engulfment | 1月19日 | 15/18723 | 1.51E-02 | 0.097727 | 0.058592 | RAB31 | 1 |
| BP | GO:2000104 | negative regulation of DNA-dependent DNA replication | 1月19日 | 15/18723 | 1.51E-02 | 0.097727 | 0.058592 | BCL6 | 1 |
| BP | GO:0030308 | negative regulation of cell growth | 2月19日 | 188/18723 | 1.53E-02 | 0.097727 | 0.058592 | BCL6/GJA1 | 2 |
| BP | GO:0001977 | renal system process involved in regulation of blood volume | 1月19日 | 16/18723 | 1.61E-02 | 0.097727 | 0.058592 | GJA1 | 1 |
| BP | GO:0002713 | negative regulation of B cell mediated immunity | 1月19日 | 16/18723 | 1.61E-02 | 0.097727 | 0.058592 | BCL6 | 1 |
| BP | GO:0002890 | negative regulation of immunoglobulin mediated immune response | 1月19日 | 16/18723 | 1.61E-02 | 0.097727 | 0.058592 | BCL6 | 1 |
| BP | GO:0006857 | oligopeptide transport | 1月19日 | 16/18723 | 1.61E-02 | 0.097727 | 0.058592 | GJA1 | 1 |
| BP | GO:0010649 | regulation of cell communication by electrical coupling | 1月19日 | 16/18723 | 1.61E-02 | 0.097727 | 0.058592 | GJA1 | 1 |
| BP | GO:0032274 | gonadotropin secretion | 1月19日 | 16/18723 | 1.61E-02 | 0.097727 | 0.058592 | GJA1 | 1 |
| BP | GO:0070431 | nucleotide-binding oligomerization domain containing 2 signaling pathway | 1月19日 | 16/18723 | 1.61E-02 | 0.097727 | 0.058592 | NFKBIA | 1 |
| BP | GO:1901163 | regulation of trophoblast cell migration | 1月19日 | 16/18723 | 1.61E-02 | 0.097727 | 0.058592 | GJA1 | 1 |
| BP | GO:1902570 | protein localization to nucleolus | 1月19日 | 16/18723 | 1.61E-02 | 0.097727 | 0.058592 | GLUL | 1 |
| BP | GO:1905153 | regulation of membrane invagination | 1月19日 | 16/18723 | 1.61E-02 | 0.097727 | 0.058592 | RAB31 | 1 |
| BP | GO:0050777 | negative regulation of immune response | 2月19日 | 194/18723 | 1.63E-02 | 0.097727 | 0.058592 | BCL6/FGR | 2 |
| BP | GO:0030073 | insulin secretion | 2月19日 | 195/18723 | 1.64E-02 | 0.097727 | 0.058592 | GLUL/GJA1 | 2 |
| BP | GO:0090276 | regulation of peptide hormone secretion | 2月19日 | 196/18723 | 1.66E-02 | 0.097727 | 0.058592 | GLUL/GJA1 | 2 |
| BP | GO:0031099 | regeneration | 2月19日 | 198/18723 | 1.69E-02 | 0.097727 | 0.058592 | CEBPB/KLF4 | 2 |
| BP | GO:0001710 | mesodermal cell fate commitment | 1月19日 | 17/18723 | 1.71E-02 | 0.097727 | 0.058592 | KLF4 | 1 |
| BP | GO:0009084 | glutamine family amino acid biosynthetic process | 1月19日 | 17/18723 | 1.71E-02 | 0.097727 | 0.058592 | GLUL | 1 |
| BP | GO:0015867 | ATP transport | 1月19日 | 17/18723 | 1.71E-02 | 0.097727 | 0.058592 | GJA1 | 1 |
| BP | GO:0016264 | gap junction assembly | 1月19日 | 17/18723 | 1.71E-02 | 0.097727 | 0.058592 | GJA1 | 1 |
| BP | GO:0020027 | hemoglobin metabolic process | 1月19日 | 17/18723 | 1.71E-02 | 0.097727 | 0.058592 | KLF4 | 1 |
| BP | GO:0043649 | dicarboxylic acid catabolic process | 1月19日 | 17/18723 | 1.71E-02 | 0.097727 | 0.058592 | GLUL | 1 |
| BP | GO:0045591 | positive regulation of regulatory T cell differentiation | 1月19日 | 17/18723 | 1.71E-02 | 0.097727 | 0.058592 | BCL6 | 1 |
| BP | GO:0060644 | mammary gland epithelial cell differentiation | 1月19日 | 17/18723 | 1.71E-02 | 0.097727 | 0.058592 | CEBPB | 1 |
| BP | GO:0061450 | trophoblast cell migration | 1月19日 | 17/18723 | 1.71E-02 | 0.097727 | 0.058592 | GJA1 | 1 |
| BP | GO:0090713 | immunological memory process | 1月19日 | 17/18723 | 1.71E-02 | 0.097727 | 0.058592 | BCL6 | 1 |
| BP | GO:0002791 | regulation of peptide secretion | 2月19日 | 200/18723 | 1.72E-02 | 0.097796 | 0.058633 | GLUL/GJA1 | 2 |
| BP | GO:0009913 | epidermal cell differentiation | 2月19日 | 202/18723 | 1.76E-02 | 0.098527 | 0.059072 | MAFF/KLF4 | 2 |
| BP | GO:0090087 | regulation of peptide transport | 2月19日 | 202/18723 | 1.76E-02 | 0.098527 | 0.059072 | GLUL/GJA1 | 2 |
| BP | GO:0060541 | respiratory system development | 2月19日 | 203/18723 | 1.77E-02 | 0.098892 | 0.059291 | EPAS1/CHI3L1 | 2 |
| BP | GO:0002544 | chronic inflammatory response | 1月19日 | 18/18723 | 1.81E-02 | 0.098898 | 0.059294 | GJA1 | 1 |
| BP | GO:0007250 | activation of NF-kappaB-inducing kinase activity | 1月19日 | 18/18723 | 1.81E-02 | 0.098898 | 0.059294 | CHI3L1 | 1 |
| BP | GO:0045116 | protein neddylation | 1月19日 | 18/18723 | 1.81E-02 | 0.098898 | 0.059294 | EPAS1 | 1 |
| BP | GO:0045623 | negative regulation of T-helper cell differentiation | 1月19日 | 18/18723 | 1.81E-02 | 0.098898 | 0.059294 | BCL6 | 1 |
| BP | GO:0008016 | regulation of heart contraction | 2月19日 | 206/18723 | 1.82E-02 | 0.098898 | 0.059294 | GJA1/EPAS1 | 2 |
| BP | GO:0045637 | regulation of myeloid cell differentiation | 2月19日 | 210/18723 | 1.89E-02 | 0.099458 | 0.05963 | NFKBIA/CEBPB | 2 |
| BP | GO:0043491 | protein kinase B signaling | 2月19日 | 211/18723 | 1.91E-02 | 0.099458 | 0.05963 | KLF4/CHI3L1 | 2 |
| BP | GO:0002902 | regulation of B cell apoptotic process | 1月19日 | 19/18723 | 1.91E-02 | 0.099458 | 0.05963 | BCL6 | 1 |
| BP | GO:0014067 | negative regulation of phosphatidylinositol 3-kinase signaling | 1月19日 | 19/18723 | 1.91E-02 | 0.099458 | 0.05963 | KLF4 | 1 |
| BP | GO:0031065 | positive regulation of histone deacetylation | 1月19日 | 19/18723 | 1.91E-02 | 0.099458 | 0.05963 | BCL6 | 1 |
| BP | GO:0086014 | atrial cardiac muscle cell action potential | 1月19日 | 19/18723 | 1.91E-02 | 0.099458 | 0.05963 | GJA1 | 1 |
| BP | GO:0086026 | atrial cardiac muscle cell to AV node cell signaling | 1月19日 | 19/18723 | 1.91E-02 | 0.099458 | 0.05963 | GJA1 | 1 |
| BP | GO:0086066 | atrial cardiac muscle cell to AV node cell communication | 1月19日 | 19/18723 | 1.91E-02 | 0.099458 | 0.05963 | GJA1 | 1 |
| BP | GO:0009749 | response to glucose | 2月19日 | 212/18723 | 1.92E-02 | 0.099513 | 0.059663 | GLUL/GJA1 | 2 |
| BP | GO:0051651 | maintenance of location in cell | 2月19日 | 214/18723 | 1.96E-02 | 0.100457 | 0.060229 | GJA1/SRGN | 2 |
| BP | GO:0014047 | glutamate secretion | 1月19日 | 20/18723 | 2.01E-02 | 0.100457 | 0.060229 | GJA1 | 1 |
| BP | GO:0032495 | response to muramyl dipeptide | 1月19日 | 20/18723 | 2.01E-02 | 0.100457 | 0.060229 | NFKBIA | 1 |
| BP | GO:0033005 | positive regulation of mast cell activation | 1月19日 | 20/18723 | 2.01E-02 | 0.100457 | 0.060229 | FGR | 1 |
| BP | GO:0060044 | negative regulation of cardiac muscle cell proliferation | 1月19日 | 20/18723 | 2.01E-02 | 0.100457 | 0.060229 | GJA1 | 1 |
| BP | GO:0072567 | chemokine (C-X-C motif) ligand 2 production | 1月19日 | 20/18723 | 2.01E-02 | 0.100457 | 0.060229 | KLF4 | 1 |
| BP | GO:2000341 | regulation of chemokine (C-X-C motif) ligand 2 production | 1月19日 | 20/18723 | 2.01E-02 | 0.100457 | 0.060229 | KLF4 | 1 |
| BP | GO:0009746 | response to hexose | 2月19日 | 219/18723 | 2.04E-02 | 0.101324 | 0.060748 | GLUL/GJA1 | 2 |
| BP | GO:0044706 | multi-multicellular organism process | 2月19日 | 220/18723 | 2.06E-02 | 0.101324 | 0.060748 | MAFF/GJA1 | 2 |
| BP | GO:0048705 | skeletal system morphogenesis | 2月19日 | 220/18723 | 2.06E-02 | 0.101324 | 0.060748 | CSRNP1/FGR | 2 |
| BP | GO:0030502 | negative regulation of bone mineralization | 1月19日 | 21/18723 | 2.11E-02 | 0.101324 | 0.060748 | SRGN | 1 |
| BP | GO:0071498 | cellular response to fluid shear stress | 1月19日 | 21/18723 | 2.11E-02 | 0.101324 | 0.060748 | KLF4 | 1 |
| BP | GO:0034284 | response to monosaccharide | 2月19日 | 225/18723 | 2.15E-02 | 0.101324 | 0.060748 | GLUL/GJA1 | 2 |
| BP | GO:0050670 | regulation of lymphocyte proliferation | 2月19日 | 225/18723 | 2.15E-02 | 0.101324 | 0.060748 | BCL6/CEBPB | 2 |
| BP | GO:0002703 | regulation of leukocyte mediated immunity | 2月19日 | 226/18723 | 2.17E-02 | 0.101324 | 0.060748 | BCL6/FGR | 2 |
| BP | GO:0032944 | regulation of mononuclear cell proliferation | 2月19日 | 227/18723 | 2.19E-02 | 0.101324 | 0.060748 | BCL6/CEBPB | 2 |
| BP | GO:0035024 | negative regulation of Rho protein signal transduction | 1月19日 | 22/18723 | 2.21E-02 | 0.101324 | 0.060748 | BCL6 | 1 |
| BP | GO:0043371 | negative regulation of CD4-positive, alpha-beta T cell differentiation | 1月19日 | 22/18723 | 2.21E-02 | 0.101324 | 0.060748 | BCL6 | 1 |
| BP | GO:0051220 | cytoplasmic sequestering of protein | 1月19日 | 22/18723 | 2.21E-02 | 0.101324 | 0.060748 | NFKBIA | 1 |
| BP | GO:0060563 | neuroepithelial cell differentiation | 1月19日 | 22/18723 | 2.21E-02 | 0.101324 | 0.060748 | CEBPB | 1 |
| BP | GO:0072574 | hepatocyte proliferation | 1月19日 | 22/18723 | 2.21E-02 | 0.101324 | 0.060748 | CEBPB | 1 |
| BP | GO:0072575 | epithelial cell proliferation involved in liver morphogenesis | 1月19日 | 22/18723 | 2.21E-02 | 0.101324 | 0.060748 | CEBPB | 1 |
| BP | GO:2000773 | negative regulation of cellular senescence | 1月19日 | 22/18723 | 2.21E-02 | 0.101324 | 0.060748 | BCL6 | 1 |
| BP | GO:0001649 | osteoblast differentiation | 2月19日 | 229/18723 | 2.22E-02 | 0.101324 | 0.060748 | VCAN/CEBPB | 2 |
| BP | GO:0045444 | fat cell differentiation | 2月19日 | 229/18723 | 2.22E-02 | 0.101324 | 0.060748 | CEBPB/KLF4 | 2 |
| BP | GO:0071356 | cellular response to tumor necrosis factor | 2月19日 | 229/18723 | 2.22E-02 | 0.101324 | 0.060748 | NFKBIA/CHI3L1 | 2 |
| BP | GO:0045055 | regulated exocytosis | 2月19日 | 230/18723 | 2.24E-02 | 0.101687 | 0.060966 | RAB31/FGR | 2 |
| BP | GO:0010594 | regulation of endothelial cell migration | 2月19日 | 232/18723 | 2.28E-02 | 0.101734 | 0.060994 | GLUL/KLF4 | 2 |
| BP | GO:0006541 | glutamine metabolic process | 1月19日 | 23/18723 | 2.31E-02 | 0.101734 | 0.060994 | GLUL | 1 |
| BP | GO:0010888 | negative regulation of lipid storage | 1月19日 | 23/18723 | 2.31E-02 | 0.101734 | 0.060994 | NFKBIA | 1 |
| BP | GO:0035994 | response to muscle stretch | 1月19日 | 23/18723 | 2.31E-02 | 0.101734 | 0.060994 | NFKBIA | 1 |
| BP | GO:0072576 | liver morphogenesis | 1月19日 | 23/18723 | 2.31E-02 | 0.101734 | 0.060994 | CEBPB | 1 |
| BP | GO:2000810 | regulation of bicellular tight junction assembly | 1月19日 | 23/18723 | 2.31E-02 | 0.101734 | 0.060994 | GJA1 | 1 |
| BP | GO:0045930 | negative regulation of mitotic cell cycle | 2月19日 | 235/18723 | 2.33E-02 | 0.101734 | 0.060994 | BCL6/KLF4 | 2 |
| BP | GO:0030072 | peptide hormone secretion | 2月19日 | 236/18723 | 2.35E-02 | 0.101734 | 0.060994 | GLUL/GJA1 | 2 |
| BP | GO:0001783 | B cell apoptotic process | 1月19日 | 24/18723 | 2.41E-02 | 0.101734 | 0.060994 | BCL6 | 1 |
| BP | GO:0006817 | phosphate ion transport | 1月19日 | 24/18723 | 2.41E-02 | 0.101734 | 0.060994 | CEBPB | 1 |
| BP | GO:0036003 | positive regulation of transcription from RNA polymerase II promoter in response to stress | 1月19日 | 24/18723 | 2.41E-02 | 0.101734 | 0.060994 | CEBPB | 1 |
| BP | GO:0043302 | positive regulation of leukocyte degranulation | 1月19日 | 24/18723 | 2.41E-02 | 0.101734 | 0.060994 | FGR | 1 |
| BP | GO:2000679 | positive regulation of transcription regulatory region DNA binding | 1月19日 | 24/18723 | 2.41E-02 | 0.101734 | 0.060994 | KLF4 | 1 |
| BP | GO:0060047 | heart contraction | 2月19日 | 241/18723 | 2.44E-02 | 0.101734 | 0.060994 | GJA1/EPAS1 | 2 |
| BP | GO:0002790 | peptide secretion | 2月19日 | 242/18723 | 2.46E-02 | 0.101734 | 0.060994 | GLUL/GJA1 | 2 |
| BP | GO:0003071 | renal system process involved in regulation of systemic arterial blood pressure | 1月19日 | 25/18723 | 2.51E-02 | 0.101734 | 0.060994 | GJA1 | 1 |
| BP | GO:0003094 | glomerular filtration | 1月19日 | 25/18723 | 2.51E-02 | 0.101734 | 0.060994 | GJA1 | 1 |
| BP | GO:0032753 | positive regulation of interleukin-4 production | 1月19日 | 25/18723 | 2.51E-02 | 0.101734 | 0.060994 | CEBPB | 1 |
| BP | GO:0046835 | carbohydrate phosphorylation | 1月19日 | 25/18723 | 2.51E-02 | 0.101734 | 0.060994 | PFKFB3 | 1 |
| BP | GO:0070423 | nucleotide-binding oligomerization domain containing signaling pathway | 1月19日 | 25/18723 | 2.51E-02 | 0.101734 | 0.060994 | NFKBIA | 1 |
| BP | GO:0086064 | cell communication by electrical coupling involved in cardiac conduction | 1月19日 | 25/18723 | 2.51E-02 | 0.101734 | 0.060994 | GJA1 | 1 |
| BP | GO:0090312 | positive regulation of protein deacetylation | 1月19日 | 25/18723 | 2.51E-02 | 0.101734 | 0.060994 | BCL6 | 1 |
| BP | GO:0090382 | phagosome maturation | 1月19日 | 25/18723 | 2.51E-02 | 0.101734 | 0.060994 | RAB31 | 1 |
| BP | GO:0070663 | regulation of leukocyte proliferation | 2月19日 | 245/18723 | 2.52E-02 | 0.101734 | 0.060994 | BCL6/CEBPB | 2 |
| BP | GO:0033002 | muscle cell proliferation | 2月19日 | 248/18723 | 2.58E-02 | 0.101734 | 0.060994 | GJA1/KLF4 | 2 |
| BP | GO:0045926 | negative regulation of growth | 2月19日 | 249/18723 | 2.60E-02 | 0.101734 | 0.060994 | BCL6/GJA1 | 2 |
| BP | GO:0046883 | regulation of hormone secretion | 2月19日 | 249/18723 | 2.60E-02 | 0.101734 | 0.060994 | GLUL/GJA1 | 2 |
| BP | GO:0010875 | positive regulation of cholesterol efflux | 1月19日 | 26/18723 | 2.61E-02 | 0.101734 | 0.060994 | NFKBIA | 1 |
| BP | GO:0032682 | negative regulation of chemokine production | 1月19日 | 26/18723 | 2.61E-02 | 0.101734 | 0.060994 | KLF4 | 1 |
| BP | GO:0033598 | mammary gland epithelial cell proliferation | 1月19日 | 26/18723 | 2.61E-02 | 0.101734 | 0.060994 | CEBPB | 1 |
| BP | GO:0034114 | regulation of heterotypic cell-cell adhesion | 1月19日 | 26/18723 | 2.61E-02 | 0.101734 | 0.060994 | KLF4 | 1 |
| BP | GO:0035872 | nucleotide-binding domain, leucine rich repeat containing receptor signaling pathway | 1月19日 | 26/18723 | 2.61E-02 | 0.101734 | 0.060994 | NFKBIA | 1 |
| BP | GO:0046639 | negative regulation of alpha-beta T cell differentiation | 1月19日 | 26/18723 | 2.61E-02 | 0.101734 | 0.060994 | BCL6 | 1 |
| BP | GO:0046697 | decidualization | 1月19日 | 26/18723 | 2.61E-02 | 0.101734 | 0.060994 | GJA1 | 1 |
| BP | GO:0097205 | renal filtration | 1月19日 | 26/18723 | 2.61E-02 | 0.101734 | 0.060994 | GJA1 | 1 |
| BP | GO:1901071 | glucosamine-containing compound metabolic process | 1月19日 | 26/18723 | 2.61E-02 | 0.101734 | 0.060994 | CHI3L1 | 1 |
| BP | GO:0003015 | heart process | 2月19日 | 251/18723 | 2.63E-02 | 0.102264 | 0.061312 | GJA1/EPAS1 | 2 |
| BP | GO:0009743 | response to carbohydrate | 2月19日 | 253/18723 | 2.67E-02 | 0.102264 | 0.061312 | GLUL/GJA1 | 2 |
| BP | GO:0034612 | response to tumor necrosis factor | 2月19日 | 253/18723 | 2.67E-02 | 0.102264 | 0.061312 | NFKBIA/CHI3L1 | 2 |
| BP | GO:0002433 | immune response-regulating cell surface receptor signaling pathway involved in phagocytosis | 1月19日 | 27/18723 | 2.71E-02 | 0.102264 | 0.061312 | FGR | 1 |
| BP | GO:0002719 | negative regulation of cytokine production involved in immune response | 1月19日 | 27/18723 | 2.71E-02 | 0.102264 | 0.061312 | BCL6 | 1 |
| BP | GO:0038096 | Fc-gamma receptor signaling pathway involved in phagocytosis | 1月19日 | 27/18723 | 2.71E-02 | 0.102264 | 0.061312 | FGR | 1 |
| BP | GO:0048520 | positive regulation of behavior | 1月19日 | 27/18723 | 2.71E-02 | 0.102264 | 0.061312 | GJA1 | 1 |
| BP | GO:1903522 | regulation of blood circulation | 2月19日 | 256/18723 | 2.73E-02 | 0.102264 | 0.061312 | GJA1/EPAS1 | 2 |
| BP | GO:0031348 | negative regulation of defense response | 2月19日 | 258/18723 | 2.77E-02 | 0.102264 | 0.061312 | KLF4/FGR | 2 |
| BP | GO:0045165 | cell fate commitment | 2月19日 | 258/18723 | 2.77E-02 | 0.102264 | 0.061312 | KLF4/EPAS1 | 2 |
| BP | GO:0009065 | glutamine family amino acid catabolic process | 1月19日 | 28/18723 | 2.80E-02 | 0.102264 | 0.061312 | GLUL | 1 |
| BP | GO:0038094 | Fc-gamma receptor signaling pathway | 1月19日 | 28/18723 | 2.80E-02 | 0.102264 | 0.061312 | FGR | 1 |
| BP | GO:0045589 | regulation of regulatory T cell differentiation | 1月19日 | 28/18723 | 2.80E-02 | 0.102264 | 0.061312 | BCL6 | 1 |
| BP | GO:0051503 | adenine nucleotide transport | 1月19日 | 28/18723 | 2.80E-02 | 0.102264 | 0.061312 | GJA1 | 1 |
| BP | GO:0060325 | face morphogenesis | 1月19日 | 28/18723 | 2.80E-02 | 0.102264 | 0.061312 | CSRNP1 | 1 |
| BP | GO:0061082 | myeloid leukocyte cytokine production | 1月19日 | 28/18723 | 2.80E-02 | 0.102264 | 0.061312 | BCL6 | 1 |
| BP | GO:0090344 | negative regulation of cell aging | 1月19日 | 28/18723 | 2.80E-02 | 0.102264 | 0.061312 | BCL6 | 1 |
| BP | GO:1903672 | positive regulation of sprouting angiogenesis | 1月19日 | 28/18723 | 2.80E-02 | 0.102264 | 0.061312 | KLF4 | 1 |
| BP | GO:0015833 | peptide transport | 2月19日 | 264/18723 | 2.89E-02 | 0.103863 | 0.062271 | GLUL/GJA1 | 2 |
| BP | GO:0002862 | negative regulation of inflammatory response to antigenic stimulus | 1月19日 | 29/18723 | 2.90E-02 | 0.103863 | 0.062271 | FGR | 1 |
| BP | GO:0015868 | purine ribonucleotide transport | 1月19日 | 29/18723 | 2.90E-02 | 0.103863 | 0.062271 | GJA1 | 1 |
| BP | GO:0048679 | regulation of axon regeneration | 1月19日 | 29/18723 | 2.90E-02 | 0.103863 | 0.062271 | KLF4 | 1 |
| BP | GO:0001894 | tissue homeostasis | 2月19日 | 268/18723 | 2.97E-02 | 0.103863 | 0.062271 | GJA1/EPAS1 | 2 |
| BP | GO:0050708 | regulation of protein secretion | 2月19日 | 268/18723 | 2.97E-02 | 0.103863 | 0.062271 | GLUL/GJA1 | 2 |
| BP | GO:0000132 | establishment of mitotic spindle orientation | 1月19日 | 30/18723 | 3.00E-02 | 0.103863 | 0.062271 | GJA1 | 1 |
| BP | GO:0010800 | positive regulation of peptidyl-threonine phosphorylation | 1月19日 | 30/18723 | 3.00E-02 | 0.103863 | 0.062271 | CHI3L1 | 1 |
| BP | GO:0015865 | purine nucleotide transport | 1月19日 | 30/18723 | 3.00E-02 | 0.103863 | 0.062271 | GJA1 | 1 |
| BP | GO:0043304 | regulation of mast cell degranulation | 1月19日 | 30/18723 | 3.00E-02 | 0.103863 | 0.062271 | FGR | 1 |
| BP | GO:0051968 | positive regulation of synaptic transmission, glutamatergic | 1月19日 | 30/18723 | 3.00E-02 | 0.103863 | 0.062271 | GLUL | 1 |
| BP | GO:0070168 | negative regulation of biomineral tissue development | 1月19日 | 30/18723 | 3.00E-02 | 0.103863 | 0.062271 | SRGN | 1 |
| BP | GO:0070229 | negative regulation of lymphocyte apoptotic process | 1月19日 | 30/18723 | 3.00E-02 | 0.103863 | 0.062271 | BCL6 | 1 |
| BP | GO:2000648 | positive regulation of stem cell proliferation | 1月19日 | 30/18723 | 3.00E-02 | 0.103863 | 0.062271 | GJA1 | 1 |
| BP | GO:0048872 | homeostasis of number of cells | 2月19日 | 272/18723 | 3.06E-02 | 0.103863 | 0.062271 | BCL6/EPAS1 | 2 |
| BP | GO:0002828 | regulation of type 2 immune response | 1月19日 | 31/18723 | 3.10E-02 | 0.103863 | 0.062271 | BCL6 | 1 |
| BP | GO:0031063 | regulation of histone deacetylation | 1月19日 | 31/18723 | 3.10E-02 | 0.103863 | 0.062271 | BCL6 | 1 |
| BP | GO:0033006 | regulation of mast cell activation involved in immune response | 1月19日 | 31/18723 | 3.10E-02 | 0.103863 | 0.062271 | FGR | 1 |
| BP | GO:0045066 | regulatory T cell differentiation | 1月19日 | 31/18723 | 3.10E-02 | 0.103863 | 0.062271 | BCL6 | 1 |
| BP | GO:0055022 | negative regulation of cardiac muscle tissue growth | 1月19日 | 31/18723 | 3.10E-02 | 0.103863 | 0.062271 | GJA1 | 1 |
| BP | GO:0060795 | cell fate commitment involved in formation of primary germ layer | 1月19日 | 31/18723 | 3.10E-02 | 0.103863 | 0.062271 | KLF4 | 1 |
| BP | GO:0061117 | negative regulation of heart growth | 1月19日 | 31/18723 | 3.10E-02 | 0.103863 | 0.062271 | GJA1 | 1 |
| BP | GO:0110150 | negative regulation of biomineralization | 1月19日 | 31/18723 | 3.10E-02 | 0.103863 | 0.062271 | SRGN | 1 |
| BP | GO:0002366 | leukocyte activation involved in immune response | 2月19日 | 275/18723 | 3.12E-02 | 0.103863 | 0.062271 | BCL6/FGR | 2 |
| BP | GO:0006026 | aminoglycan catabolic process | 1月19日 | 32/18723 | 3.20E-02 | 0.103863 | 0.062271 | CHI3L1 | 1 |
| BP | GO:0008156 | negative regulation of DNA replication | 1月19日 | 32/18723 | 3.20E-02 | 0.103863 | 0.062271 | BCL6 | 1 |
| BP | GO:0010644 | cell communication by electrical coupling | 1月19日 | 32/18723 | 3.20E-02 | 0.103863 | 0.062271 | GJA1 | 1 |
| BP | GO:0010743 | regulation of macrophage derived foam cell differentiation | 1月19日 | 32/18723 | 3.20E-02 | 0.103863 | 0.062271 | NFKBIA | 1 |
| BP | GO:0060323 | head morphogenesis | 1月19日 | 32/18723 | 3.20E-02 | 0.103863 | 0.062271 | CSRNP1 | 1 |
| BP | GO:0072337 | modified amino acid transport | 1月19日 | 32/18723 | 3.20E-02 | 0.103863 | 0.062271 | GJA1 | 1 |
| BP | GO:2000515 | negative regulation of CD4-positive, alpha-beta T cell activation | 1月19日 | 32/18723 | 3.20E-02 | 0.103863 | 0.062271 | BCL6 | 1 |
| BP | GO:0002263 | cell activation involved in immune response | 2月19日 | 279/18723 | 3.20E-02 | 0.103863 | 0.062271 | BCL6/FGR | 2 |
| BP | GO:0043542 | endothelial cell migration | 2月19日 | 279/18723 | 3.20E-02 | 0.103863 | 0.062271 | GLUL/KLF4 | 2 |
| BP | GO:1902105 | regulation of leukocyte differentiation | 2月19日 | 279/18723 | 3.20E-02 | 0.103863 | 0.062271 | BCL6/CEBPB | 2 |
| BP | GO:0007249 | I-kappaB kinase/NF-kappaB signaling | 2月19日 | 281/18723 | 3.24E-02 | 0.103927 | 0.062309 | NFKBIA/GJA1 | 2 |
| BP | GO:0002431 | Fc receptor mediated stimulatory signaling pathway | 1月19日 | 33/18723 | 3.30E-02 | 0.103927 | 0.062309 | FGR | 1 |
| BP | GO:0006536 | glutamate metabolic process | 1月19日 | 33/18723 | 3.30E-02 | 0.103927 | 0.062309 | GLUL | 1 |
| BP | GO:0018345 | protein palmitoylation | 1月19日 | 33/18723 | 3.30E-02 | 0.103927 | 0.062309 | GLUL | 1 |
| BP | GO:0032633 | interleukin-4 production | 1月19日 | 33/18723 | 3.30E-02 | 0.103927 | 0.062309 | CEBPB | 1 |
| BP | GO:0032673 | regulation of interleukin-4 production | 1月19日 | 33/18723 | 3.30E-02 | 0.103927 | 0.062309 | CEBPB | 1 |
| BP | GO:0043552 | positive regulation of phosphatidylinositol 3-kinase activity | 1月19日 | 33/18723 | 3.30E-02 | 0.103927 | 0.062309 | FGR | 1 |
| BP | GO:0048730 | epidermis morphogenesis | 1月19日 | 33/18723 | 3.30E-02 | 0.103927 | 0.062309 | KLF4 | 1 |
| BP | GO:0070570 | regulation of neuron projection regeneration | 1月19日 | 33/18723 | 3.30E-02 | 0.103927 | 0.062309 | KLF4 | 1 |
| BP | GO:0034599 | cellular response to oxidative stress | 2月19日 | 288/18723 | 3.39E-02 | 0.104203 | 0.062475 | KLF4/EPAS1 | 2 |
| BP | GO:0046651 | lymphocyte proliferation | 2月19日 | 288/18723 | 3.39E-02 | 0.104203 | 0.062475 | BCL6/CEBPB | 2 |
| BP | GO:0001662 | behavioral fear response | 1月19日 | 34/18723 | 3.40E-02 | 0.104203 | 0.062475 | GJA1 | 1 |
| BP | GO:0050869 | negative regulation of B cell activation | 1月19日 | 34/18723 | 3.40E-02 | 0.104203 | 0.062475 | BCL6 | 1 |
| BP | GO:0051973 | positive regulation of telomerase activity | 1月19日 | 34/18723 | 3.40E-02 | 0.104203 | 0.062475 | KLF4 | 1 |
| BP | GO:0086019 | cell-cell signaling involved in cardiac conduction | 1月19日 | 34/18723 | 3.40E-02 | 0.104203 | 0.062475 | GJA1 | 1 |
| BP | GO:0034504 | protein localization to nucleus | 2月19日 | 290/18723 | 3.44E-02 | 0.104203 | 0.062475 | NFKBIA/GLUL | 2 |
| BP | GO:0032943 | mononuclear cell proliferation | 2月19日 | 291/18723 | 3.46E-02 | 0.104203 | 0.062475 | BCL6/CEBPB | 2 |
| BP | GO:0010632 | regulation of epithelial cell migration | 2月19日 | 292/18723 | 3.48E-02 | 0.104203 | 0.062475 | GLUL/KLF4 | 2 |
| BP | GO:0001893 | maternal placenta development | 1月19日 | 35/18723 | 3.49E-02 | 0.104203 | 0.062475 | GJA1 | 1 |
| BP | GO:0002209 | behavioral defense response | 1月19日 | 35/18723 | 3.49E-02 | 0.104203 | 0.062475 | GJA1 | 1 |
| BP | GO:0032814 | regulation of natural killer cell activation | 1月19日 | 35/18723 | 3.49E-02 | 0.104203 | 0.062475 | FGR | 1 |
| BP | GO:0035633 | maintenance of blood-brain barrier | 1月19日 | 35/18723 | 3.49E-02 | 0.104203 | 0.062475 | GJA1 | 1 |
| BP | GO:0040001 | establishment of mitotic spindle localization | 1月19日 | 35/18723 | 3.49E-02 | 0.104203 | 0.062475 | GJA1 | 1 |
| BP | GO:0045907 | positive regulation of vasoconstriction | 1月19日 | 35/18723 | 3.49E-02 | 0.104203 | 0.062475 | GJA1 | 1 |
| BP | GO:0048821 | erythrocyte development | 1月19日 | 35/18723 | 3.49E-02 | 0.104203 | 0.062475 | BCL6 | 1 |
| BP | GO:0097421 | liver regeneration | 1月19日 | 35/18723 | 3.49E-02 | 0.104203 | 0.062475 | CEBPB | 1 |
| BP | GO:0098801 | regulation of renal system process | 1月19日 | 35/18723 | 3.49E-02 | 0.104203 | 0.062475 | GJA1 | 1 |
| BP | GO:0010948 | negative regulation of cell cycle process | 2月19日 | 294/18723 | 3.52E-02 | 0.104794 | 0.062829 | BCL6/KLF4 | 2 |
| BP | GO:0046879 | hormone secretion | 2月19日 | 295/18723 | 3.55E-02 | 0.105133 | 0.063032 | GLUL/GJA1 | 2 |
| BP | GO:0042092 | type 2 immune response | 1月19日 | 36/18723 | 3.59E-02 | 0.105504 | 0.063254 | BCL6 | 1 |
| BP | GO:0044060 | regulation of endocrine process | 1月19日 | 36/18723 | 3.59E-02 | 0.105504 | 0.063254 | GJA1 | 1 |
| BP | GO:0045191 | regulation of isotype switching | 1月19日 | 36/18723 | 3.59E-02 | 0.105504 | 0.063254 | BCL6 | 1 |
| BP | GO:0042886 | amide transport | 2月19日 | 301/18723 | 3.68E-02 | 0.105504 | 0.063254 | GLUL/GJA1 | 2 |
| BP | GO:0003161 | cardiac conduction system development | 1月19日 | 37/18723 | 3.69E-02 | 0.105504 | 0.063254 | GJA1 | 1 |
| BP | GO:0006862 | nucleotide transport | 1月19日 | 37/18723 | 3.69E-02 | 0.105504 | 0.063254 | GJA1 | 1 |
| BP | GO:0033260 | nuclear DNA replication | 1月19日 | 37/18723 | 3.69E-02 | 0.105504 | 0.063254 | BCL6 | 1 |
| BP | GO:0033363 | secretory granule organization | 1月19日 | 37/18723 | 3.69E-02 | 0.105504 | 0.063254 | SRGN | 1 |
| BP | GO:0051294 | establishment of spindle orientation | 1月19日 | 37/18723 | 3.69E-02 | 0.105504 | 0.063254 | GJA1 | 1 |
| BP | GO:0090218 | positive regulation of lipid kinase activity | 1月19日 | 37/18723 | 3.69E-02 | 0.105504 | 0.063254 | FGR | 1 |
| BP | GO:1905332 | positive regulation of morphogenesis of an epithelium | 1月19日 | 37/18723 | 3.69E-02 | 0.105504 | 0.063254 | GJA1 | 1 |
| BP | GO:0051056 | regulation of small GTPase mediated signal transduction | 2月19日 | 302/18723 | 3.70E-02 | 0.105504 | 0.063254 | ARHGEF40/BCL6 | 2 |
| BP | GO:0051222 | positive regulation of protein transport | 2月19日 | 303/18723 | 3.72E-02 | 0.105504 | 0.063254 | GLUL/GJA1 | 2 |
| BP | GO:0010742 | macrophage derived foam cell differentiation | 1月19日 | 38/18723 | 3.79E-02 | 0.105504 | 0.063254 | NFKBIA | 1 |
| BP | GO:0030279 | negative regulation of ossification | 1月19日 | 38/18723 | 3.79E-02 | 0.105504 | 0.063254 | SRGN | 1 |
| BP | GO:0032373 | positive regulation of sterol transport | 1月19日 | 38/18723 | 3.79E-02 | 0.105504 | 0.063254 | NFKBIA | 1 |
| BP | GO:0032376 | positive regulation of cholesterol transport | 1月19日 | 38/18723 | 3.79E-02 | 0.105504 | 0.063254 | NFKBIA | 1 |
| BP | GO:0032717 | negative regulation of interleukin-8 production | 1月19日 | 38/18723 | 3.79E-02 | 0.105504 | 0.063254 | KLF4 | 1 |
| BP | GO:0042596 | fear response | 1月19日 | 38/18723 | 3.79E-02 | 0.105504 | 0.063254 | GJA1 | 1 |
| BP | GO:0090077 | foam cell differentiation | 1月19日 | 38/18723 | 3.79E-02 | 0.105504 | 0.063254 | NFKBIA | 1 |
| BP | GO:2000279 | negative regulation of DNA biosynthetic process | 1月19日 | 38/18723 | 3.79E-02 | 0.105504 | 0.063254 | GJA1 | 1 |
| BP | GO:0009914 | hormone transport | 2月19日 | 306/18723 | 3.79E-02 | 0.105504 | 0.063254 | GLUL/GJA1 | 2 |
| BP | GO:0006909 | phagocytosis | 2月19日 | 308/18723 | 3.84E-02 | 0.106365 | 0.063771 | RAB31/FGR | 2 |
| BP | GO:0070372 | regulation of ERK1 and ERK2 cascade | 2月19日 | 309/18723 | 3.86E-02 | 0.106365 | 0.063771 | KLF4/CHI3L1 | 2 |
| BP | GO:0002347 | response to tumor cell | 1月19日 | 39/18723 | 3.89E-02 | 0.106365 | 0.063771 | KLF4 | 1 |
| BP | GO:0002701 | negative regulation of production of molecular mediator of immune response | 1月19日 | 39/18723 | 3.89E-02 | 0.106365 | 0.063771 | BCL6 | 1 |
| BP | GO:0045622 | regulation of T-helper cell differentiation | 1月19日 | 39/18723 | 3.89E-02 | 0.106365 | 0.063771 | BCL6 | 1 |
| BP | GO:0046621 | negative regulation of organ growth | 1月19日 | 39/18723 | 3.89E-02 | 0.106365 | 0.063771 | GJA1 | 1 |
| BP | GO:0060249 | anatomical structure homeostasis | 2月19日 | 314/18723 | 3.97E-02 | 0.107858 | 0.064666 | GJA1/EPAS1 | 2 |
| BP | GO:0043001 | Golgi to plasma membrane protein transport | 1月19日 | 40/18723 | 3.98E-02 | 0.107858 | 0.064666 | RAB31 | 1 |
| BP | GO:0045429 | positive regulation of nitric oxide biosynthetic process | 1月19日 | 40/18723 | 3.98E-02 | 0.107858 | 0.064666 | KLF4 | 1 |
| BP | GO:1904994 | regulation of leukocyte adhesion to vascular endothelial cell | 1月19日 | 40/18723 | 3.98E-02 | 0.107858 | 0.064666 | KLF4 | 1 |
| BP | GO:0070661 | leukocyte proliferation | 2月19日 | 318/18723 | 4.07E-02 | 0.109222 | 0.065484 | BCL6/CEBPB | 2 |
| BP | GO:0001953 | negative regulation of cell-matrix adhesion | 1月19日 | 41/18723 | 4.08E-02 | 0.109222 | 0.065484 | BCL6 | 1 |
| BP | GO:0044786 | cell cycle DNA replication | 1月19日 | 41/18723 | 4.08E-02 | 0.109222 | 0.065484 | BCL6 | 1 |
| BP | GO:1904407 | positive regulation of nitric oxide metabolic process | 1月19日 | 41/18723 | 4.08E-02 | 0.109222 | 0.065484 | KLF4 | 1 |
| BP | GO:1904951 | positive regulation of establishment of protein localization | 2月19日 | 319/18723 | 4.09E-02 | 0.109222 | 0.065484 | GLUL/GJA1 | 2 |
| BP | GO:0071496 | cellular response to external stimulus | 2月19日 | 320/18723 | 4.11E-02 | 0.10955 | 0.06568 | GLUL/GJA1 | 2 |
| BP | GO:0006040 | amino sugar metabolic process | 1月19日 | 42/18723 | 4.18E-02 | 0.109579 | 0.065698 | CHI3L1 | 1 |
| BP | GO:0030890 | positive regulation of B cell proliferation | 1月19日 | 42/18723 | 4.18E-02 | 0.109579 | 0.065698 | BCL6 | 1 |
| BP | GO:0033003 | regulation of mast cell activation | 1月19日 | 42/18723 | 4.18E-02 | 0.109579 | 0.065698 | FGR | 1 |
| BP | GO:0072595 | maintenance of protein localization in organelle | 1月19日 | 42/18723 | 4.18E-02 | 0.109579 | 0.065698 | GJA1 | 1 |
| BP | GO:0090051 | negative regulation of cell migration involved in sprouting angiogenesis | 1月19日 | 42/18723 | 4.18E-02 | 0.109579 | 0.065698 | KLF4 | 1 |
| BP | GO:1902895 | positive regulation of pri-miRNA transcription by RNA polymerase II | 1月19日 | 42/18723 | 4.18E-02 | 0.109579 | 0.065698 | KLF4 | 1 |
| BP | GO:0008544 | epidermis development | 2月19日 | 324/18723 | 4.21E-02 | 0.109828 | 0.065847 | MAFF/KLF4 | 2 |
| BP | GO:0001709 | cell fate determination | 1月19日 | 43/18723 | 4.28E-02 | 0.109828 | 0.065847 | KLF4 | 1 |
| BP | GO:0002861 | regulation of inflammatory response to antigenic stimulus | 1月19日 | 43/18723 | 4.28E-02 | 0.109828 | 0.065847 | FGR | 1 |
| BP | GO:0009268 | response to pH | 1月19日 | 43/18723 | 4.28E-02 | 0.109828 | 0.065847 | GJA1 | 1 |
| BP | GO:0010171 | body morphogenesis | 1月19日 | 43/18723 | 4.28E-02 | 0.109828 | 0.065847 | CSRNP1 | 1 |
| BP | GO:0034142 | toll-like receptor 4 signaling pathway | 1月19日 | 43/18723 | 4.28E-02 | 0.109828 | 0.065847 | NFKBIA | 1 |
| BP | GO:0046636 | negative regulation of alpha-beta T cell activation | 1月19日 | 43/18723 | 4.28E-02 | 0.109828 | 0.065847 | BCL6 | 1 |
| BP | GO:1903793 | positive regulation of anion transport | 1月19日 | 43/18723 | 4.28E-02 | 0.109828 | 0.065847 | CEBPB | 1 |
| BP | GO:0050863 | regulation of T cell activation | 2月19日 | 329/18723 | 4.32E-02 | 0.110757 | 0.066404 | BCL6/CEBPB | 2 |
| BP | GO:0070371 | ERK1 and ERK2 cascade | 2月19日 | 330/18723 | 4.35E-02 | 0.111081 | 0.066598 | KLF4/CHI3L1 | 2 |
| BP | GO:0060324 | face development | 1月19日 | 44/18723 | 4.37E-02 | 0.111469 | 0.066831 | CSRNP1 | 1 |
| BP | GO:0045910 | negative regulation of DNA recombination | 1月19日 | 45/18723 | 4.47E-02 | 0.11337 | 0.06797 | BCL6 | 1 |
| BP | GO:1904646 | cellular response to amyloid-beta | 1月19日 | 45/18723 | 4.47E-02 | 0.11337 | 0.06797 | GJA1 | 1 |
| BP | GO:0062197 | cellular response to chemical stress | 2月19日 | 337/18723 | 4.52E-02 | 0.114096 | 0.068406 | KLF4/EPAS1 | 2 |
| BP | GO:0002697 | regulation of immune effector process | 2月19日 | 339/18723 | 4.56E-02 | 0.114096 | 0.068406 | BCL6/FGR | 2 |
| BP | GO:0001974 | blood vessel remodeling | 1月19日 | 46/18723 | 4.57E-02 | 0.114096 | 0.068406 | EPAS1 | 1 |
| BP | GO:0043300 | regulation of leukocyte degranulation | 1月19日 | 46/18723 | 4.57E-02 | 0.114096 | 0.068406 | FGR | 1 |
| BP | GO:0090329 | regulation of DNA-dependent DNA replication | 1月19日 | 46/18723 | 4.57E-02 | 0.114096 | 0.068406 | BCL6 | 1 |
| BP | GO:2000107 | negative regulation of leukocyte apoptotic process | 1月19日 | 46/18723 | 4.57E-02 | 0.114096 | 0.068406 | BCL6 | 1 |
| BP | GO:0010799 | regulation of peptidyl-threonine phosphorylation | 1月19日 | 47/18723 | 4.67E-02 | 0.114493 | 0.068644 | CHI3L1 | 1 |
| BP | GO:0042311 | vasodilation | 1月19日 | 47/18723 | 4.67E-02 | 0.114493 | 0.068644 | GJA1 | 1 |
| BP | GO:0045581 | negative regulation of T cell differentiation | 1月19日 | 47/18723 | 4.67E-02 | 0.114493 | 0.068644 | BCL6 | 1 |
| BP | GO:0051972 | regulation of telomerase activity | 1月19日 | 47/18723 | 4.67E-02 | 0.114493 | 0.068644 | KLF4 | 1 |
| BP | GO:0097028 | dendritic cell differentiation | 1月19日 | 47/18723 | 4.67E-02 | 0.114493 | 0.068644 | CEBPB | 1 |
| BP | GO:0030336 | negative regulation of cell migration | 2月19日 | 344/18723 | 4.69E-02 | 0.114493 | 0.068644 | GJA1/KLF4 | 2 |
| BP | GO:0042908 | xenobiotic transport | 1月19日 | 48/18723 | 4.76E-02 | 0.114493 | 0.068644 | GJA1 | 1 |
| BP | GO:0043303 | mast cell degranulation | 1月19日 | 48/18723 | 4.76E-02 | 0.114493 | 0.068644 | FGR | 1 |
| BP | GO:0090311 | regulation of protein deacetylation | 1月19日 | 48/18723 | 4.76E-02 | 0.114493 | 0.068644 | BCL6 | 1 |
| BP | GO:0043087 | regulation of GTPase activity | 2月19日 | 348/18723 | 4.78E-02 | 0.114493 | 0.068644 | BCL6/RGS1 | 2 |
| BP | GO:0042742 | defense response to bacterium | 2月19日 | 350/18723 | 4.83E-02 | 0.114493 | 0.068644 | CEBPB/FGR | 2 |
| BP | GO:0002204 | somatic recombination of immunoglobulin genes involved in immune response | 1月19日 | 49/18723 | 4.86E-02 | 0.114493 | 0.068644 | BCL6 | 1 |
| BP | GO:0002208 | somatic diversification of immunoglobulins involved in immune response | 1月19日 | 49/18723 | 4.86E-02 | 0.114493 | 0.068644 | BCL6 | 1 |
| BP | GO:0002279 | mast cell activation involved in immune response | 1月19日 | 49/18723 | 4.86E-02 | 0.114493 | 0.068644 | FGR | 1 |
| BP | GO:0006953 | acute-phase response | 1月19日 | 49/18723 | 4.86E-02 | 0.114493 | 0.068644 | CEBPB | 1 |
| BP | GO:0009409 | response to cold | 1月19日 | 49/18723 | 4.86E-02 | 0.114493 | 0.068644 | NFKBIA | 1 |
| BP | GO:0043330 | response to exogenous dsRNA | 1月19日 | 49/18723 | 4.86E-02 | 0.114493 | 0.068644 | NFKBIA | 1 |
| BP | GO:0045190 | isotype switching | 1月19日 | 49/18723 | 4.86E-02 | 0.114493 | 0.068644 | BCL6 | 1 |
| BP | GO:0046580 | negative regulation of Ras protein signal transduction | 1月19日 | 49/18723 | 4.86E-02 | 0.114493 | 0.068644 | BCL6 | 1 |
| BP | GO:0060986 | endocrine hormone secretion | 1月19日 | 49/18723 | 4.86E-02 | 0.114493 | 0.068644 | GJA1 | 1 |
| BP | GO:0070169 | positive regulation of biomineral tissue development | 1月19日 | 49/18723 | 4.86E-02 | 0.114493 | 0.068644 | CEBPB | 1 |
| BP | GO:0070741 | response to interleukin-6 | 1月19日 | 49/18723 | 4.86E-02 | 0.114493 | 0.068644 | CHI3L1 | 1 |
| BP | GO:0072091 | regulation of stem cell proliferation | 1月19日 | 49/18723 | 4.86E-02 | 0.114493 | 0.068644 | GJA1 | 1 |
| BP | GO:1904707 | positive regulation of vascular associated smooth muscle cell proliferation | 1月19日 | 49/18723 | 4.86E-02 | 0.114493 | 0.068644 | GJA1 | 1 |
| BP | GO:0006887 | exocytosis | 2月19日 | 352/18723 | 4.88E-02 | 0.114778 | 0.068815 | RAB31/FGR | 2 |
| BP | GO:0002448 | mast cell mediated immunity | 1月19日 | 50/18723 | 4.96E-02 | 0.115145 | 0.069035 | FGR | 1 |
| BP | GO:0038093 | Fc receptor signaling pathway | 1月19日 | 50/18723 | 4.96E-02 | 0.115145 | 0.069035 | FGR | 1 |
| BP | GO:0050873 | brown fat cell differentiation | 1月19日 | 50/18723 | 4.96E-02 | 0.115145 | 0.069035 | CEBPB | 1 |
| BP | GO:0110151 | positive regulation of biomineralization | 1月19日 | 50/18723 | 4.96E-02 | 0.115145 | 0.069035 | CEBPB | 1 |
| BP | GO:2000677 | regulation of transcription regulatory region DNA binding | 1月19日 | 50/18723 | 4.96E-02 | 0.115145 | 0.069035 | KLF4 | 1 |
| CC | GO:0034774 | secretory granule lumen | 4月19日 | 322/19550 | 2.30E-04 | 0.007082 | 0.00557 | SRGN/FGR/CXCL1/CHI3L1 | 4 |
| CC | GO:0060205 | cytoplasmic vesicle lumen | 4月19日 | 325/19550 | 2.39E-04 | 0.007082 | 0.00557 | SRGN/FGR/CXCL1/CHI3L1 | 4 |
| CC | GO:0031983 | vesicle lumen | 4月19日 | 327/19550 | 2.44E-04 | 0.007082 | 0.00557 | SRGN/FGR/CXCL1/CHI3L1 | 4 |
| CC | GO:0035580 | specific granule lumen | 2月19日 | 62/19550 | 1.63E-03 | 0.035548 | 0.027956 | CXCL1/CHI3L1 | 2 |
| CC | GO:0031234 | extrinsic component of cytoplasmic side of plasma membrane | 2月19日 | 101/19550 | 4.27E-03 | 0.074251 | 0.058395 | FGR/RGS1 | 2 |
| CC | GO:0005667 | transcription regulator complex | 3月19日 | 413/19550 | 7.05E-03 | 0.09644 | 0.075845 | CEBPB/KLF4/EPAS1 | 3 |
| CC | GO:0005916 | fascia adherens | 1月19日 | 10/19550 | 9.68E-03 | 0.09644 | 0.075845 | GJA1 | 1 |
| CC | GO:0042581 | specific granule | 2月19日 | 160/19550 | 1.04E-02 | 0.09644 | 0.075845 | CXCL1/CHI3L1 | 2 |
| CC | GO:0032009 | early phagosome | 1月19日 | 12/19550 | 1.16E-02 | 0.09644 | 0.075845 | RAB31 | 1 |
| CC | GO:0009898 | cytoplasmic side of plasma membrane | 2月19日 | 172/19550 | 1.19E-02 | 0.09644 | 0.075845 | FGR/RGS1 | 2 |
| CC | GO:0019897 | extrinsic component of plasma membrane | 2月19日 | 174/19550 | 1.22E-02 | 0.09644 | 0.075845 | FGR/RGS1 | 2 |
| CC | GO:0098562 | cytoplasmic side of membrane | 2月19日 | 197/19550 | 1.54E-02 | 0.111903 | 0.088006 | FGR/RGS1 | 2 |
| CC | GO:0099091 | postsynaptic specialization, intracellular component | 1月19日 | 19/19550 | 1.83E-02 | 0.122558 | 0.096385 | SRGN | 1 |
| CC | GO:0005922 | connexin complex | 1月19日 | 21/19550 | 2.02E-02 | 0.125667 | 0.098831 | GJA1 | 1 |
| CC | GO:0001891 | phagocytic cup | 1月19日 | 28/19550 | 2.69E-02 | 0.155884 | 0.122595 | RAB31 | 1 |
| CC | GO:0005921 | gap junction | 1月19日 | 32/19550 | 3.07E-02 | 0.157941 | 0.124213 | GJA1 | 1 |
| CC | GO:0097386 | glial cell projection | 1月19日 | 33/19550 | 3.16E-02 | 0.157941 | 0.124213 | GLUL | 1 |
| CC | GO:0016235 | aggresome | 1月19日 | 35/19550 | 3.35E-02 | 0.157941 | 0.124213 | FGR | 1 |
| CC | GO:0019898 | extrinsic component of membrane | 2月19日 | 309/19550 | 3.57E-02 | 0.157941 | 0.124213 | FGR/RGS1 | 2 |
| CC | GO:0000791 | euchromatin | 1月19日 | 38/19550 | 3.63E-02 | 0.157941 | 0.124213 | KLF4 | 1 |
| CC | GO:0043209 | myelin sheath | 1月19日 | 48/19550 | 4.57E-02 | 0.187889 | 0.147765 | GLUL | 1 |
| CC | GO:0014704 | intercalated disc | 1月19日 | 50/19550 | 4.75E-02 | 0.187889 | 0.147765 | GJA1 | 1 |
| MF | GO:0001228 | DNA-binding transcription activator activity, RNA polymerase II-specific | 6月19日 | 450/18368 | 4.33E-06 | 0.000236 | 0.00013 | MAFF/CEBPB/CSRNP1/CEBPD/KLF4/EPAS1 | 6 |
| MF | GO:0001216 | DNA-binding transcription activator activity | 6月19日 | 456/18368 | 4.67E-06 | 0.000236 | 0.00013 | MAFF/CEBPB/CSRNP1/CEBPD/KLF4/EPAS1 | 6 |
| MF | GO:0001221 | transcription coregulator binding | 2月19日 | 78/18368 | 2.90E-03 | 0.069459 | 0.038367 | KLF4/EPAS1 | 2 |
| MF | GO:0061629 | RNA polymerase II-specific DNA-binding transcription factor binding | 3月19日 | 299/18368 | 3.41E-03 | 0.069459 | 0.038367 | NFKBIA/CEBPB/KLF4 | 3 |
| MF | GO:0008013 | beta-catenin binding | 2月19日 | 85/18368 | 3.44E-03 | 0.069459 | 0.038367 | GJA1/KLF4 | 2 |
| MF | GO:0140297 | DNA-binding transcription factor binding | 3月19日 | 394/18368 | 7.35E-03 | 0.08797 | 0.048592 | NFKBIA/CEBPB/KLF4 | 3 |
| MF | GO:0042826 | histone deacetylase binding | 2月19日 | 127/18368 | 7.51E-03 | 0.08797 | 0.048592 | CEBPB/KLF4 | 2 |
| MF | GO:0016595 | glutamate binding | 1月19日 | 10/18368 | 1.03E-02 | 0.08797 | 0.048592 | GLUL | 1 |
| MF | GO:0019203 | carbohydrate phosphatase activity | 1月19日 | 10/18368 | 1.03E-02 | 0.08797 | 0.048592 | PFKFB3 | 1 |
| MF | GO:0045503 | dynein light chain binding | 1月19日 | 10/18368 | 1.03E-02 | 0.08797 | 0.048592 | GLUL | 1 |
| MF | GO:0050308 | sugar-phosphatase activity | 1月19日 | 10/18368 | 1.03E-02 | 0.08797 | 0.048592 | PFKFB3 | 1 |
| MF | GO:0001161 | intronic transcription regulatory region sequence-specific DNA binding | 1月19日 | 11/18368 | 1.13E-02 | 0.08797 | 0.048592 | BCL6 | 1 |
| MF | GO:0035673 | oligopeptide transmembrane transporter activity | 1月19日 | 11/18368 | 1.13E-02 | 0.08797 | 0.048592 | GJA1 | 1 |
| MF | GO:0005201 | extracellular matrix structural constituent | 2月19日 | 172/18368 | 1.34E-02 | 0.090836 | 0.050175 | VCAN/CHI3L1 | 2 |
| MF | GO:0015562 | efflux transmembrane transporter activity | 1月19日 | 14/18368 | 1.44E-02 | 0.090836 | 0.050175 | GJA1 | 1 |
| MF | GO:0035259 | glucocorticoid receptor binding | 1月19日 | 14/18368 | 1.44E-02 | 0.090836 | 0.050175 | CEBPB | 1 |
| MF | GO:1904680 | peptide transmembrane transporter activity | 1月19日 | 15/18368 | 1.54E-02 | 0.090998 | 0.050265 | GJA1 | 1 |
| MF | GO:0045236 | CXCR chemokine receptor binding | 1月19日 | 18/18368 | 1.85E-02 | 0.090998 | 0.050265 | CXCL1 | 1 |
| MF | GO:0000979 | RNA polymerase II core promoter sequence-specific DNA binding | 1月19日 | 20/18368 | 2.05E-02 | 0.090998 | 0.050265 | CEBPB | 1 |
| MF | GO:0035014 | phosphatidylinositol 3-kinase regulator activity | 1月19日 | 20/18368 | 2.05E-02 | 0.090998 | 0.050265 | KLF4 | 1 |
| MF | GO:0005243 | gap junction channel activity | 1月19日 | 21/18368 | 2.15E-02 | 0.090998 | 0.050265 | GJA1 | 1 |
| MF | GO:0019200 | carbohydrate kinase activity | 1月19日 | 21/18368 | 2.15E-02 | 0.090998 | 0.050265 | PFKFB3 | 1 |
| MF | GO:0005540 | hyaluronic acid binding | 1月19日 | 22/18368 | 2.25E-02 | 0.090998 | 0.050265 | VCAN | 1 |
| MF | GO:0030021 | extracellular matrix structural constituent conferring compression resistance | 1月19日 | 22/18368 | 2.25E-02 | 0.090998 | 0.050265 | VCAN | 1 |
| MF | GO:0072349 | modified amino acid transmembrane transporter activity | 1月19日 | 22/18368 | 2.25E-02 | 0.090998 | 0.050265 | GJA1 | 1 |
| MF | GO:0001965 | G-protein alpha-subunit binding | 1月19日 | 24/18368 | 2.45E-02 | 0.092528 | 0.05111 | RGS1 | 1 |
| MF | GO:0035035 | histone acetyltransferase binding | 1月19日 | 25/18368 | 2.56E-02 | 0.092528 | 0.05111 | CEBPB | 1 |
| MF | GO:0019706 | protein-cysteine S-palmitoyltransferase activity | 1月19日 | 26/18368 | 2.66E-02 | 0.092528 | 0.05111 | GLUL | 1 |
| MF | GO:0019707 | protein-cysteine S-acyltransferase activity | 1月19日 | 26/18368 | 2.66E-02 | 0.092528 | 0.05111 | GLUL | 1 |
| MF | GO:0008139 | nuclear localization sequence binding | 1月19日 | 27/18368 | 2.76E-02 | 0.092839 | 0.051282 | NFKBIA | 1 |
| MF | GO:0051059 | NF-kappaB binding | 1月19日 | 29/18368 | 2.96E-02 | 0.093396 | 0.051589 | NFKBIA | 1 |
| MF | GO:0016417 | S-acyltransferase activity | 1月19日 | 30/18368 | 3.06E-02 | 0.093396 | 0.051589 | GLUL | 1 |
| MF | GO:0022829 | wide pore channel activity | 1月19日 | 30/18368 | 3.06E-02 | 0.093396 | 0.051589 | GJA1 | 1 |
| MF | GO:0030246 | carbohydrate binding | 2月19日 | 271/18368 | 3.14E-02 | 0.093396 | 0.051589 | VCAN/CHI3L1 | 2 |
| MF | GO:0001223 | transcription coactivator binding | 1月19日 | 32/18368 | 3.26E-02 | 0.094083 | 0.051968 | EPAS1 | 1 |
| MF | GO:0042887 | amide transmembrane transporter activity | 1月19日 | 35/18368 | 3.56E-02 | 0.099898 | 0.055181 | GJA1 | 1 |
| MF | GO:0097718 | disordered domain specific binding | 1月19日 | 36/18368 | 3.66E-02 | 0.099926 | 0.055196 | GJA1 | 1 |
| MF | GO:0016409 | palmitoyltransferase activity | 1月19日 | 37/18368 | 3.76E-02 | 0.099951 | 0.05521 | GLUL | 1 |
| MF | GO:0001227 | DNA-binding transcription repressor activity, RNA polymerase II-specific | 2月19日 | 310/18368 | 4.02E-02 | 0.102252 | 0.056481 | BCL6/CEBPB | 2 |
| MF | GO:0001217 | DNA-binding transcription repressor activity | 2月19日 | 313/18368 | 4.09E-02 | 0.102252 | 0.056481 | BCL6/CEBPB | 2 |
| MF | GO:0044389 | ubiquitin-like protein ligase binding | 2月19日 | 316/18368 | 4.16E-02 | 0.102252 | 0.056481 | NFKBIA/CEBPB | 2 |
| MF | GO:0001046 | core promoter sequence-specific DNA binding | 1月19日 | 42/18368 | 4.26E-02 | 0.102252 | 0.056481 | CEBPB | 1 |
| MF | GO:0046982 | protein heterodimerization activity | 2月19日 | 328/18368 | 4.45E-02 | 0.102252 | 0.056481 | CEBPB/EPAS1 | 2 |
| MF | GO:0001784 | phosphotyrosine residue binding | 1月19日 | 44/18368 | 4.46E-02 | 0.102252 | 0.056481 | FGR | 1 |
| MF | GO:0004715 | non-membrane spanning protein tyrosine kinase activity | 1月19日 | 45/18368 | 4.56E-02 | 0.102252 | 0.056481 | FGR | 1 |
| MF | GO:0003924 | GTPase activity | 2月19日 | 337/18368 | 4.67E-02 | 0.102613 | 0.056681 | RAB31/RGS1 | 2 |
| MF | GO:0016879 | ligase activity, forming carbon-nitrogen bonds | 1月19日 | 48/18368 | 4.85E-02 | 0.103902 | 0.057393 | GLUL | 1 |
| MF | GO:0008009 | chemokine activity | 1月19日 | 49/18368 | 4.95E-02 | 0.103902 | 0.057393 | CXCL1 | 1 |

Supplementary Table 3. The GO results.
