# Supplementary material for: Exploring the pathogenesis and key genes associated of acute myocardial infarction complicated with Alzheimer’s disease
Source: Sci Rep. 2024 Jan 16;14:1449. doi: 10.1038/s41598-024-52094-4 (PMC10791667; doi:10.1038/s41598-024-52094-4)
Supplement: Supplementary file 4 — Supplementary Table 4. [file 41598_2024_52094_MOESM4_ESM.docx]

| ID | Description | GeneRatio | BgRatio | pvalue | p.adjust | qvalue | geneID | Count |
| --- | --- | --- | --- | --- | --- | --- | --- | --- |
| hsa04657 | IL-17 signaling pathway | 3月12日 | 94/8461 | 2.72E-04 | 1.73E-02 | 0.014138 | NFKBIA/CEBPB/CXCL1 | 3 |
| hsa04668 | TNF signaling pathway | 3月12日 | 114/8461 | 4.80E-04 | 1.73E-02 | 0.014138 | NFKBIA/CEBPB/CXCL1 | 3 |
| hsa04062 | Chemokine signaling pathway | 3月12日 | 192/8461 | 2.18E-03 | 5.07E-02 | 0.041533 | NFKBIA/FGR/CXCL1 | 3 |
| hsa05134 | Legionellosis | 2月12日 | 57/8461 | 2.82E-03 | 5.07E-02 | 0.041533 | NFKBIA/CXCL1 | 2 |
| hsa05120 | Epithelial cell signaling in Helicobacter pylori infection | 2月12日 | 70/8461 | 4.22E-03 | 6.08E-02 | 0.049764 | NFKBIA/CXCL1 | 2 |
| hsa04064 | NF-kappa B signaling pathway | 2月12日 | 104/8461 | 9.11E-03 | 1.09E-01 | 0.089543 | NFKBIA/CXCL1 | 2 |
| hsa04936 | Alcoholic liver disease | 2月12日 | 142/8461 | 1.65E-02 | 1.70E-01 | 0.139228 | NFKBIA/CXCL1 | 2 |
| hsa00910 | Nitrogen metabolism | 1月12日 | 17/8461 | 2.39E-02 | 1.84E-01 | 0.151045 | GLUL | 1 |
| hsa04621 | NOD-like receptor signaling pathway | 2月12日 | 186/8461 | 2.74E-02 | 1.84E-01 | 0.151045 | NFKBIA/CXCL1 | 2 |
| hsa05202 | Transcriptional misregulation in cancer | 2月12日 | 193/8461 | 2.94E-02 | 1.84E-01 | 0.151045 | BCL6/CEBPB | 2 |
| hsa05167 | Kaposi sarcoma-associated herpesvirus infection | 2月12日 | 194/8461 | 2.97E-02 | 1.84E-01 | 0.151045 | NFKBIA/CXCL1 | 2 |
| hsa00220 | Arginine biosynthesis | 1月12日 | 22/8461 | 3.08E-02 | 1.84E-01 | 0.151045 | GLUL | 1 |
| hsa05207 | Chemical carcinogenesis - receptor activation | 2月12日 | 212/8461 | 3.50E-02 | 1.84E-01 | 0.151045 | BCL6/KLF4 | 2 |
| hsa05417 | Lipid and atherosclerosis | 2月12日 | 215/8461 | 3.59E-02 | 1.84E-01 | 0.151045 | NFKBIA/CXCL1 | 2 |
| hsa00630 | Glyoxylate and dicarboxylate metabolism | 1月12日 | 30/8461 | 4.18E-02 | 2.00E-01 | 0.164089 | GLUL | 1 |
| hsa00051 | Fructose and mannose metabolism | 1月12日 | 33/8461 | 4.58E-02 | 2.06E-01 | 0.168888 | PFKFB3 | 1 |

Supplementary Table 4. The KEGG results.
