# Supplementary material for: Exploring the pathogenesis and key genes associated of acute myocardial infarction complicated with Alzheimer’s disease
Source: Sci Rep. 2024 Jan 16;14:1449. doi: 10.1038/s41598-024-52094-4 (PMC10791667; doi:10.1038/s41598-024-52094-4)
Supplement: Supplementary file 5 — Supplementary Table 5. [file 41598_2024_52094_MOESM5_ESM.docx]

| ID |
| --- |
| \| GJA1 \| \| --- \| \| CEBPD \| \| RGS1 \| \| SRGN \| \| FGR \| \| NFKBIA \| \| KLF4 \| \| BCL6 \| \| CXCL1 \| \| CEBPB \| |

Supplementary Table 5. The top ten key genes.
