# Supplementary material for: Exploring the pathogenesis and key genes associated of acute myocardial infarction complicated with Alzheimer’s disease
Source: Sci Rep. 2024 Jan 16;14:1449. doi: 10.1038/s41598-024-52094-4 (PMC10791667; doi:10.1038/s41598-024-52094-4)
Supplement: Supplementary file 6 — Supplementary Table 6. [file 41598_2024_52094_MOESM6_ESM.docx]

| ONTOLOGY | ID | Description | GeneRatio | BgRatio | pvalue | p.adjust | qvalue | geneID | Count |
| --- | --- | --- | --- | --- | --- | --- | --- | --- | --- |
| BP | GO:0050727 | regulation of inflammatory response | 5月10日 | 414/18614 | 1.22E-06 | 0.00089 | 0.000402 | FGR/NFKBIA/KLF4/BCL6/CEBPB | 5 |
| BP | GO:0001503 | ossification | 4月10日 | 429/18614 | 5.23E-05 | 0.012797 | 0.005783 | CEBPD/SRGN/FGR/CEBPB | 4 |
| BP | GO:1903038 | negative regulation of leukocyte cell-cell adhesion | 3月10日 | 150/18614 | 5.91E-05 | 0.012797 | 0.005783 | KLF4/BCL6/CEBPB | 3 |
| BP | GO:0051250 | negative regulation of lymphocyte activation | 3月10日 | 168/18614 | 8.27E-05 | 0.012797 | 0.005783 | FGR/BCL6/CEBPB | 3 |
| BP | GO:0031214 | biomineral tissue development | 3月10日 | 173/18614 | 9.02E-05 | 0.012797 | 0.005783 | SRGN/FGR/CEBPB | 3 |
| BP | GO:0002695 | negative regulation of leukocyte activation | 3月10日 | 202/18614 | 1.43E-04 | 0.012797 | 0.005783 | FGR/BCL6/CEBPB | 3 |
| BP | GO:0022408 | negative regulation of cell-cell adhesion | 3月10日 | 205/18614 | 1.49E-04 | 0.012797 | 0.005783 | KLF4/BCL6/CEBPB | 3 |
| BP | GO:0048333 | mesodermal cell differentiation | 2月10日 | 35/18614 | 1.53E-04 | 0.012797 | 0.005783 | GJA1/KLF4 | 2 |
| BP | GO:0045746 | negative regulation of Notch signaling pathway | 2月10日 | 37/18614 | 1.71E-04 | 0.012797 | 0.005783 | NFKBIA/BCL6 | 2 |
| BP | GO:0071222 | cellular response to lipopolysaccharide | 3月10日 | 224/18614 | 1.94E-04 | 0.012797 | 0.005783 | NFKBIA/CXCL1/CEBPB | 3 |
| BP | GO:0050866 | negative regulation of cell activation | 3月10日 | 225/18614 | 1.96E-04 | 0.012797 | 0.005783 | FGR/BCL6/CEBPB | 3 |
| BP | GO:0140467 | integrated stress response signaling | 2月10日 | 41/18614 | 2.11E-04 | 0.012797 | 0.005783 | CEBPD/CEBPB | 2 |
| BP | GO:0071219 | cellular response to molecule of bacterial origin | 3月10日 | 237/18614 | 2.29E-04 | 0.012838 | 0.005802 | NFKBIA/CXCL1/CEBPB | 3 |
| BP | GO:0045444 | fat cell differentiation | 3月10日 | 246/18614 | 2.55E-04 | 0.013303 | 0.006012 | CEBPD/KLF4/CEBPB | 3 |
| BP | GO:0071216 | cellular response to biotic stimulus | 3月10日 | 264/18614 | 3.14E-04 | 0.015281 | 0.006906 | NFKBIA/CXCL1/CEBPB | 3 |
| BP | GO:0007162 | negative regulation of cell adhesion | 3月10日 | 312/18614 | 5.13E-04 | 0.023368 | 0.010561 | KLF4/BCL6/CEBPB | 3 |
| BP | GO:0032496 | response to lipopolysaccharide | 3月10日 | 345/18614 | 6.88E-04 | 0.027085 | 0.012241 | NFKBIA/CXCL1/CEBPB | 3 |
| BP | GO:0001707 | mesoderm formation | 2月10日 | 75/18614 | 7.06E-04 | 0.027085 | 0.012241 | GJA1/KLF4 | 2 |
| BP | GO:0045669 | positive regulation of osteoblast differentiation | 2月10日 | 75/18614 | 7.06E-04 | 0.027085 | 0.012241 | CEBPD/CEBPB | 2 |
| BP | GO:0048332 | mesoderm morphogenesis | 2月10日 | 77/18614 | 7.44E-04 | 0.027116 | 0.012255 | GJA1/KLF4 | 2 |
| BP | GO:0002237 | response to molecule of bacterial origin | 3月10日 | 366/18614 | 8.16E-04 | 0.027234 | 0.012309 | NFKBIA/CXCL1/CEBPB | 3 |
| BP | GO:0001818 | negative regulation of cytokine production | 3月10日 | 371/18614 | 8.49E-04 | 0.027234 | 0.012309 | SRGN/KLF4/BCL6 | 3 |
| BP | GO:0061515 | myeloid cell development | 2月10日 | 84/18614 | 8.85E-04 | 0.027234 | 0.012309 | BCL6/CEBPB | 2 |
| BP | GO:1903037 | regulation of leukocyte cell-cell adhesion | 3月10日 | 378/18614 | 8.97E-04 | 0.027234 | 0.012309 | KLF4/BCL6/CEBPB | 3 |
| BP | GO:0045185 | maintenance of protein location | 2月10日 | 92/18614 | 1.06E-03 | 0.027899 | 0.012609 | SRGN/NFKBIA | 2 |
| BP | GO:0032088 | negative regulation of NF-kappaB transcription factor activity | 2月10日 | 96/18614 | 1.15E-03 | 0.027899 | 0.012609 | NFKBIA/KLF4 | 2 |
| BP | GO:0070664 | negative regulation of leukocyte proliferation | 2月10日 | 96/18614 | 1.15E-03 | 0.027899 | 0.012609 | BCL6/CEBPB | 2 |
| BP | GO:0007159 | leukocyte cell-cell adhesion | 3月10日 | 415/18614 | 1.17E-03 | 0.027899 | 0.012609 | KLF4/BCL6/CEBPB | 3 |
| BP | GO:1903706 | regulation of hemopoiesis | 3月10日 | 415/18614 | 1.17E-03 | 0.027899 | 0.012609 | NFKBIA/BCL6/CEBPB | 3 |
| BP | GO:0008593 | regulation of Notch signaling pathway | 2月10日 | 97/18614 | 1.18E-03 | 0.027899 | 0.012609 | NFKBIA/BCL6 | 2 |
| BP | GO:0120162 | positive regulation of cold-induced thermogenesis | 2月10日 | 98/18614 | 1.20E-03 | 0.027899 | 0.012609 | GJA1/CEBPB | 2 |
| BP | GO:0030099 | myeloid cell differentiation | 3月10日 | 421/18614 | 1.22E-03 | 0.027899 | 0.012609 | NFKBIA/BCL6/CEBPB | 3 |
| BP | GO:0070167 | regulation of biomineral tissue development | 2月10日 | 101/18614 | 1.28E-03 | 0.028169 | 0.012731 | SRGN/CEBPB | 2 |
| BP | GO:0002065 | columnar/cuboidal epithelial cell differentiation | 2月10日 | 118/18614 | 1.73E-03 | 0.03657 | 0.016528 | KLF4/CEBPB | 2 |
| BP | GO:0051101 | regulation of DNA binding | 2月10日 | 120/18614 | 1.79E-03 | 0.03657 | 0.016528 | NFKBIA/KLF4 | 2 |
| BP | GO:0030282 | bone mineralization | 2月10日 | 121/18614 | 1.82E-03 | 0.03657 | 0.016528 | SRGN/FGR | 2 |
| BP | GO:0002683 | negative regulation of immune system process | 3月10日 | 487/18614 | 1.86E-03 | 0.03657 | 0.016528 | FGR/BCL6/CEBPB | 3 |
| BP | GO:0022407 | regulation of cell-cell adhesion | 3月10日 | 491/18614 | 1.91E-03 | 0.03657 | 0.016528 | KLF4/BCL6/CEBPB | 3 |
| BP | GO:0001704 | formation of primary germ layer | 2月10日 | 127/18614 | 2.01E-03 | 0.037483 | 0.01694 | GJA1/KLF4 | 2 |
| BP | GO:0050868 | negative regulation of T cell activation | 2月10日 | 131/18614 | 2.13E-03 | 0.038849 | 0.017558 | BCL6/CEBPB | 2 |
| BP | GO:0007498 | mesoderm development | 2月10日 | 137/18614 | 2.33E-03 | 0.041395 | 0.018709 | GJA1/KLF4 | 2 |
| BP | GO:0045667 | regulation of osteoblast differentiation | 2月10日 | 147/18614 | 2.67E-03 | 0.044899 | 0.020292 | CEBPD/CEBPB | 2 |
| BP | GO:0120161 | regulation of cold-induced thermogenesis | 2月10日 | 147/18614 | 2.67E-03 | 0.044899 | 0.020292 | GJA1/CEBPB | 2 |
| BP | GO:0106106 | cold-induced thermogenesis | 2月10日 | 148/18614 | 2.71E-03 | 0.044899 | 0.020292 | GJA1/CEBPB | 2 |
| BP | GO:0050729 | positive regulation of inflammatory response | 2月10日 | 154/18614 | 2.93E-03 | 0.047463 | 0.021451 | NFKBIA/CEBPB | 2 |
| BP | GO:1990845 | adaptive thermogenesis | 2月10日 | 161/18614 | 3.20E-03 | 0.050661 | 0.022896 | GJA1/CEBPB | 2 |
| BP | GO:0001659 | temperature homeostasis | 2月10日 | 179/18614 | 3.93E-03 | 0.059742 | 0.027 | GJA1/CEBPB | 2 |
| BP | GO:0007219 | Notch signaling pathway | 2月10日 | 179/18614 | 3.93E-03 | 0.059742 | 0.027 | NFKBIA/BCL6 | 2 |
| BP | GO:0042129 | regulation of T cell proliferation | 2月10日 | 183/18614 | 4.11E-03 | 0.061105 | 0.027616 | BCL6/CEBPB | 2 |
| BP | GO:0050728 | negative regulation of inflammatory response | 2月10日 | 187/18614 | 4.28E-03 | 0.061413 | 0.027756 | FGR/KLF4 | 2 |
| BP | GO:0031099 | regeneration | 2月10日 | 190/18614 | 4.42E-03 | 0.061413 | 0.027756 | KLF4/CEBPB | 2 |
| BP | GO:0030308 | negative regulation of cell growth | 2月10日 | 191/18614 | 4.46E-03 | 0.061413 | 0.027756 | GJA1/BCL6 | 2 |
| BP | GO:0050777 | negative regulation of immune response | 2月10日 | 191/18614 | 4.46E-03 | 0.061413 | 0.027756 | FGR/BCL6 | 2 |
| BP | GO:0043433 | negative regulation of DNA-binding transcription factor activity | 2月10日 | 194/18614 | 4.60E-03 | 0.062136 | 0.028082 | NFKBIA/KLF4 | 2 |
| BP | GO:0007369 | gastrulation | 2月10日 | 196/18614 | 4.70E-03 | 0.062238 | 0.028129 | GJA1/KLF4 | 2 |
| BP | GO:0032815 | negative regulation of natural killer cell activation | 1月10日 | 10/18614 | 5.36E-03 | 0.063249 | 0.028586 | FGR | 1 |
| BP | GO:0044341 | sodium-dependent phosphate transport | 1月10日 | 10/18614 | 5.36E-03 | 0.063249 | 0.028586 | CEBPB | 1 |
| BP | GO:0042098 | T cell proliferation | 2月10日 | 213/18614 | 5.52E-03 | 0.063249 | 0.028586 | BCL6/CEBPB | 2 |
| BP | GO:0045637 | regulation of myeloid cell differentiation | 2月10日 | 214/18614 | 5.57E-03 | 0.063249 | 0.028586 | NFKBIA/CEBPB | 2 |
| BP | GO:0032276 | regulation of gonadotropin secretion | 1月10日 | 11/18614 | 5.90E-03 | 0.063249 | 0.028586 | GJA1 | 1 |
| BP | GO:0034115 | negative regulation of heterotypic cell-cell adhesion | 1月10日 | 11/18614 | 5.90E-03 | 0.063249 | 0.028586 | KLF4 | 1 |
| BP | GO:0045628 | regulation of T-helper 2 cell differentiation | 1月10日 | 11/18614 | 5.90E-03 | 0.063249 | 0.028586 | BCL6 | 1 |
| BP | GO:0070587 | regulation of cell-cell adhesion involved in gastrulation | 1月10日 | 11/18614 | 5.90E-03 | 0.063249 | 0.028586 | KLF4 | 1 |
| BP | GO:1990440 | positive regulation of transcription from RNA polymerase II promoter in response to endoplasmic reticulum stress | 1月10日 | 11/18614 | 5.90E-03 | 0.063249 | 0.028586 | CEBPB | 1 |
| BP | GO:0043380 | regulation of memory T cell differentiation | 1月10日 | 12/18614 | 6.43E-03 | 0.063249 | 0.028586 | BCL6 | 1 |
| BP | GO:0070586 | cell-cell adhesion involved in gastrulation | 1月10日 | 12/18614 | 6.43E-03 | 0.063249 | 0.028586 | KLF4 | 1 |
| BP | GO:2001198 | regulation of dendritic cell differentiation | 1月10日 | 12/18614 | 6.43E-03 | 0.063249 | 0.028586 | CEBPB | 1 |
| BP | GO:0050670 | regulation of lymphocyte proliferation | 2月10日 | 239/18614 | 6.90E-03 | 0.063249 | 0.028586 | BCL6/CEBPB | 2 |
| BP | GO:0002357 | defense response to tumor cell | 1月10日 | 13/18614 | 6.96E-03 | 0.063249 | 0.028586 | KLF4 | 1 |
| BP | GO:0002638 | negative regulation of immunoglobulin production | 1月10日 | 13/18614 | 6.96E-03 | 0.063249 | 0.028586 | BCL6 | 1 |
| BP | GO:0010745 | negative regulation of macrophage derived foam cell differentiation | 1月10日 | 13/18614 | 6.96E-03 | 0.063249 | 0.028586 | NFKBIA | 1 |
| BP | GO:0033262 | regulation of nuclear cell cycle DNA replication | 1月10日 | 13/18614 | 6.96E-03 | 0.063249 | 0.028586 | BCL6 | 1 |
| BP | GO:0034616 | response to laminar fluid shear stress | 1月10日 | 13/18614 | 6.96E-03 | 0.063249 | 0.028586 | KLF4 | 1 |
| BP | GO:0043379 | memory T cell differentiation | 1月10日 | 13/18614 | 6.96E-03 | 0.063249 | 0.028586 | BCL6 | 1 |
| BP | GO:0070417 | cellular response to cold | 1月10日 | 13/18614 | 6.96E-03 | 0.063249 | 0.028586 | NFKBIA | 1 |
| BP | GO:1904995 | negative regulation of leukocyte adhesion to vascular endothelial cell | 1月10日 | 13/18614 | 6.96E-03 | 0.063249 | 0.028586 | KLF4 | 1 |
| BP | GO:0032944 | regulation of mononuclear cell proliferation | 2月10日 | 243/18614 | 7.13E-03 | 0.063249 | 0.028586 | BCL6/CEBPB | 2 |
| BP | GO:0045930 | negative regulation of mitotic cell cycle | 2月10日 | 243/18614 | 7.13E-03 | 0.063249 | 0.028586 | KLF4/BCL6 | 2 |
| BP | GO:0002703 | regulation of leukocyte mediated immunity | 2月10日 | 246/18614 | 7.30E-03 | 0.063249 | 0.028586 | FGR/BCL6 | 2 |
| BP | GO:0002467 | germinal center formation | 1月10日 | 14/18614 | 7.50E-03 | 0.063249 | 0.028586 | BCL6 | 1 |
| BP | GO:0002829 | negative regulation of type 2 immune response | 1月10日 | 14/18614 | 7.50E-03 | 0.063249 | 0.028586 | BCL6 | 1 |
| BP | GO:0021859 | pyramidal neuron differentiation | 1月10日 | 14/18614 | 7.50E-03 | 0.063249 | 0.028586 | BCL6 | 1 |
| BP | GO:0042541 | hemoglobin biosynthetic process | 1月10日 | 14/18614 | 7.50E-03 | 0.063249 | 0.028586 | KLF4 | 1 |
| BP | GO:0090715 | immunological memory formation process | 1月10日 | 14/18614 | 7.50E-03 | 0.063249 | 0.028586 | BCL6 | 1 |
| BP | GO:1902969 | mitotic DNA replication | 1月10日 | 14/18614 | 7.50E-03 | 0.063249 | 0.028586 | BCL6 | 1 |
| BP | GO:0001649 | osteoblast differentiation | 2月10日 | 250/18614 | 7.53E-03 | 0.063249 | 0.028586 | CEBPD/CEBPB | 2 |
| BP | GO:0045926 | negative regulation of growth | 2月10日 | 252/18614 | 7.65E-03 | 0.063249 | 0.028586 | GJA1/BCL6 | 2 |
| BP | GO:0033008 | positive regulation of mast cell activation involved in immune response | 1月10日 | 15/18614 | 8.03E-03 | 0.063249 | 0.028586 | FGR | 1 |
| BP | GO:0042994 | cytoplasmic sequestering of transcription factor | 1月10日 | 15/18614 | 8.03E-03 | 0.063249 | 0.028586 | NFKBIA | 1 |
| BP | GO:0043306 | positive regulation of mast cell degranulation | 1月10日 | 15/18614 | 8.03E-03 | 0.063249 | 0.028586 | FGR | 1 |
| BP | GO:0002713 | negative regulation of B cell mediated immunity | 1月10日 | 16/18614 | 8.56E-03 | 0.063249 | 0.028586 | BCL6 | 1 |
| BP | GO:0002890 | negative regulation of immunoglobulin mediated immune response | 1月10日 | 16/18614 | 8.56E-03 | 0.063249 | 0.028586 | BCL6 | 1 |
| BP | GO:0002903 | negative regulation of B cell apoptotic process | 1月10日 | 16/18614 | 8.56E-03 | 0.063249 | 0.028586 | BCL6 | 1 |
| BP | GO:0070431 | nucleotide-binding oligomerization domain containing 2 signaling pathway | 1月10日 | 16/18614 | 8.56E-03 | 0.063249 | 0.028586 | NFKBIA | 1 |
| BP | GO:2000104 | negative regulation of DNA-templated DNA replication | 1月10日 | 16/18614 | 8.56E-03 | 0.063249 | 0.028586 | BCL6 | 1 |
| BP | GO:0070663 | regulation of leukocyte proliferation | 2月10日 | 268/18614 | 8.61E-03 | 0.063249 | 0.028586 | BCL6/CEBPB | 2 |
| BP | GO:0032274 | gonadotropin secretion | 1月10日 | 17/18614 | 9.10E-03 | 0.063249 | 0.028586 | GJA1 | 1 |
| BP | GO:0090713 | immunological memory process | 1月10日 | 17/18614 | 9.10E-03 | 0.063249 | 0.028586 | BCL6 | 1 |
| BP | GO:1901163 | regulation of trophoblast cell migration | 1月10日 | 17/18614 | 9.10E-03 | 0.063249 | 0.028586 | GJA1 | 1 |
| BP | GO:0031348 | negative regulation of defense response | 2月10日 | 284/18614 | 9.63E-03 | 0.063249 | 0.028586 | FGR/KLF4 | 2 |
| BP | GO:0001710 | mesodermal cell fate commitment | 1月10日 | 18/18614 | 9.63E-03 | 0.063249 | 0.028586 | KLF4 | 1 |
| BP | GO:0016264 | gap junction assembly | 1月10日 | 18/18614 | 9.63E-03 | 0.063249 | 0.028586 | GJA1 | 1 |
| BP | GO:0020027 | hemoglobin metabolic process | 1月10日 | 18/18614 | 9.63E-03 | 0.063249 | 0.028586 | KLF4 | 1 |
| BP | GO:0036003 | positive regulation of transcription from RNA polymerase II promoter in response to stress | 1月10日 | 18/18614 | 9.63E-03 | 0.063249 | 0.028586 | CEBPB | 1 |
| BP | GO:0045064 | T-helper 2 cell differentiation | 1月10日 | 18/18614 | 9.63E-03 | 0.063249 | 0.028586 | BCL6 | 1 |
| BP | GO:0045623 | negative regulation of T-helper cell differentiation | 1月10日 | 18/18614 | 9.63E-03 | 0.063249 | 0.028586 | BCL6 | 1 |
| BP | GO:0048569 | post-embryonic animal organ development | 1月10日 | 18/18614 | 9.63E-03 | 0.063249 | 0.028586 | KLF4 | 1 |
| BP | GO:0060644 | mammary gland epithelial cell differentiation | 1月10日 | 18/18614 | 9.63E-03 | 0.063249 | 0.028586 | CEBPB | 1 |
| BP | GO:0061450 | trophoblast cell migration | 1月10日 | 18/18614 | 9.63E-03 | 0.063249 | 0.028586 | GJA1 | 1 |
| BP | GO:0072567 | chemokine (C-X-C motif) ligand 2 production | 1月10日 | 18/18614 | 9.63E-03 | 0.063249 | 0.028586 | KLF4 | 1 |
| BP | GO:2000341 | regulation of chemokine (C-X-C motif) ligand 2 production | 1月10日 | 18/18614 | 9.63E-03 | 0.063249 | 0.028586 | KLF4 | 1 |
| BP | GO:0086014 | atrial cardiac muscle cell action potential | 1月10日 | 19/18614 | 1.02E-02 | 0.064803 | 0.029288 | GJA1 | 1 |
| BP | GO:0086026 | atrial cardiac muscle cell to AV node cell signaling | 1月10日 | 19/18614 | 1.02E-02 | 0.064803 | 0.029288 | GJA1 | 1 |
| BP | GO:0086066 | atrial cardiac muscle cell to AV node cell communication | 1月10日 | 19/18614 | 1.02E-02 | 0.064803 | 0.029288 | GJA1 | 1 |
| BP | GO:0007249 | I-kappaB kinase/NF-kappaB signaling | 2月10日 | 293/18614 | 1.02E-02 | 0.064803 | 0.029288 | GJA1/NFKBIA | 2 |
| BP | GO:0002366 | leukocyte activation involved in immune response | 2月10日 | 296/18614 | 1.04E-02 | 0.064959 | 0.029358 | FGR/BCL6 | 2 |
| BP | GO:0032495 | response to muramyl dipeptide | 1月10日 | 20/18614 | 1.07E-02 | 0.064959 | 0.029358 | NFKBIA | 1 |
| BP | GO:0002263 | cell activation involved in immune response | 2月10日 | 300/18614 | 1.07E-02 | 0.064959 | 0.029358 | FGR/BCL6 | 2 |
| BP | GO:0002429 | immune response-activating cell surface receptor signaling pathway | 2月10日 | 302/18614 | 1.08E-02 | 0.064959 | 0.029358 | FGR/NFKBIA | 2 |
| BP | GO:0046651 | lymphocyte proliferation | 2月10日 | 306/18614 | 1.11E-02 | 0.064959 | 0.029358 | BCL6/CEBPB | 2 |
| BP | GO:0002902 | regulation of B cell apoptotic process | 1月10日 | 21/18614 | 1.12E-02 | 0.064959 | 0.029358 | BCL6 | 1 |
| BP | GO:0010888 | negative regulation of lipid storage | 1月10日 | 21/18614 | 1.12E-02 | 0.064959 | 0.029358 | NFKBIA | 1 |
| BP | GO:0030502 | negative regulation of bone mineralization | 1月10日 | 21/18614 | 1.12E-02 | 0.064959 | 0.029358 | SRGN | 1 |
| BP | GO:0033005 | positive regulation of mast cell activation | 1月10日 | 21/18614 | 1.12E-02 | 0.064959 | 0.029358 | FGR | 1 |
| BP | GO:0051220 | cytoplasmic sequestering of protein | 1月10日 | 21/18614 | 1.12E-02 | 0.064959 | 0.029358 | NFKBIA | 1 |
| BP | GO:0071498 | cellular response to fluid shear stress | 1月10日 | 21/18614 | 1.12E-02 | 0.064959 | 0.029358 | KLF4 | 1 |
| BP | GO:0032943 | mononuclear cell proliferation | 2月10日 | 313/18614 | 1.16E-02 | 0.065882 | 0.029776 | BCL6/CEBPB | 2 |
| BP | GO:0042742 | defense response to bacterium | 2月10日 | 314/18614 | 1.17E-02 | 0.065882 | 0.029776 | FGR/CEBPB | 2 |
| BP | GO:0035024 | negative regulation of Rho protein signal transduction | 1月10日 | 22/18614 | 1.18E-02 | 0.065882 | 0.029776 | BCL6 | 1 |
| BP | GO:0043371 | negative regulation of CD4-positive, alpha-beta T cell differentiation | 1月10日 | 22/18614 | 1.18E-02 | 0.065882 | 0.029776 | BCL6 | 1 |
| BP | GO:0010948 | negative regulation of cell cycle process | 2月10日 | 317/18614 | 1.19E-02 | 0.065882 | 0.029776 | KLF4/BCL6 | 2 |
| BP | GO:1902105 | regulation of leukocyte differentiation | 2月10日 | 319/18614 | 1.20E-02 | 0.065882 | 0.029776 | BCL6/CEBPB | 2 |
| BP | GO:0031065 | positive regulation of histone deacetylation | 1月10日 | 23/18614 | 1.23E-02 | 0.065882 | 0.029776 | BCL6 | 1 |
| BP | GO:0045591 | positive regulation of regulatory T cell differentiation | 1月10日 | 23/18614 | 1.23E-02 | 0.065882 | 0.029776 | BCL6 | 1 |
| BP | GO:0072574 | hepatocyte proliferation | 1月10日 | 23/18614 | 1.23E-02 | 0.065882 | 0.029776 | CEBPB | 1 |
| BP | GO:0072575 | epithelial cell proliferation involved in liver morphogenesis | 1月10日 | 23/18614 | 1.23E-02 | 0.065882 | 0.029776 | CEBPB | 1 |
| BP | GO:0002768 | immune response-regulating cell surface receptor signaling pathway | 2月10日 | 329/18614 | 1.28E-02 | 0.066507 | 0.030058 | FGR/NFKBIA | 2 |
| BP | GO:0072576 | liver morphogenesis | 1月10日 | 24/18614 | 1.28E-02 | 0.066507 | 0.030058 | CEBPB | 1 |
| BP | GO:0014047 | glutamate secretion | 1月10日 | 25/18614 | 1.34E-02 | 0.066507 | 0.030058 | GJA1 | 1 |
| BP | GO:0034114 | regulation of heterotypic cell-cell adhesion | 1月10日 | 25/18614 | 1.34E-02 | 0.066507 | 0.030058 | KLF4 | 1 |
| BP | GO:0043302 | positive regulation of leukocyte degranulation | 1月10日 | 25/18614 | 1.34E-02 | 0.066507 | 0.030058 | FGR | 1 |
| BP | GO:0070423 | nucleotide-binding oligomerization domain containing signaling pathway | 1月10日 | 25/18614 | 1.34E-02 | 0.066507 | 0.030058 | NFKBIA | 1 |
| BP | GO:0086064 | cell communication by electrical coupling involved in cardiac conduction | 1月10日 | 25/18614 | 1.34E-02 | 0.066507 | 0.030058 | GJA1 | 1 |
| BP | GO:2000679 | positive regulation of transcription regulatory region DNA binding | 1月10日 | 25/18614 | 1.34E-02 | 0.066507 | 0.030058 | KLF4 | 1 |
| BP | GO:0002433 | immune response-regulating cell surface receptor signaling pathway involved in phagocytosis | 1月10日 | 26/18614 | 1.39E-02 | 0.066507 | 0.030058 | FGR | 1 |
| BP | GO:0006817 | phosphate ion transport | 1月10日 | 26/18614 | 1.39E-02 | 0.066507 | 0.030058 | CEBPB | 1 |
| BP | GO:0033598 | mammary gland epithelial cell proliferation | 1月10日 | 26/18614 | 1.39E-02 | 0.066507 | 0.030058 | CEBPB | 1 |
| BP | GO:0035872 | nucleotide-binding domain, leucine rich repeat containing receptor signaling pathway | 1月10日 | 26/18614 | 1.39E-02 | 0.066507 | 0.030058 | NFKBIA | 1 |
| BP | GO:0035994 | response to muscle stretch | 1月10日 | 26/18614 | 1.39E-02 | 0.066507 | 0.030058 | NFKBIA | 1 |
| BP | GO:0038096 | Fc-gamma receptor signaling pathway involved in phagocytosis | 1月10日 | 26/18614 | 1.39E-02 | 0.066507 | 0.030058 | FGR | 1 |
| BP | GO:2000773 | negative regulation of cellular senescence | 1月10日 | 26/18614 | 1.39E-02 | 0.066507 | 0.030058 | BCL6 | 1 |
| BP | GO:0051235 | maintenance of location | 2月10日 | 347/18614 | 1.41E-02 | 0.066507 | 0.030058 | SRGN/NFKBIA | 2 |
| BP | GO:0070661 | leukocyte proliferation | 2月10日 | 348/18614 | 1.42E-02 | 0.066507 | 0.030058 | BCL6/CEBPB | 2 |
| BP | GO:0001783 | B cell apoptotic process | 1月10日 | 27/18614 | 1.44E-02 | 0.066507 | 0.030058 | BCL6 | 1 |
| BP | GO:0010875 | positive regulation of cholesterol efflux | 1月10日 | 27/18614 | 1.44E-02 | 0.066507 | 0.030058 | NFKBIA | 1 |
| BP | GO:0032682 | negative regulation of chemokine production | 1月10日 | 27/18614 | 1.44E-02 | 0.066507 | 0.030058 | KLF4 | 1 |
| BP | GO:0032753 | positive regulation of interleukin-4 production | 1月10日 | 27/18614 | 1.44E-02 | 0.066507 | 0.030058 | CEBPB | 1 |
| BP | GO:0046639 | negative regulation of alpha-beta T cell differentiation | 1月10日 | 27/18614 | 1.44E-02 | 0.066507 | 0.030058 | BCL6 | 1 |
| BP | GO:0048679 | regulation of axon regeneration | 1月10日 | 28/18614 | 1.49E-02 | 0.067564 | 0.030536 | KLF4 | 1 |
| BP | GO:1903672 | positive regulation of sprouting angiogenesis | 1月10日 | 28/18614 | 1.49E-02 | 0.067564 | 0.030536 | KLF4 | 1 |
| BP | GO:0043087 | regulation of GTPase activity | 2月10日 | 364/18614 | 1.55E-02 | 0.067564 | 0.030536 | RGS1/BCL6 | 2 |
| BP | GO:0061436 | establishment of skin barrier | 1月10日 | 29/18614 | 1.55E-02 | 0.067564 | 0.030536 | KLF4 | 1 |
| BP | GO:0051098 | regulation of binding | 2月10日 | 369/18614 | 1.59E-02 | 0.067564 | 0.030536 | NFKBIA/KLF4 | 2 |
| BP | GO:0070168 | negative regulation of biomineral tissue development | 1月10日 | 30/18614 | 1.60E-02 | 0.067564 | 0.030536 | SRGN | 1 |
| BP | GO:0090312 | positive regulation of protein deacetylation | 1月10日 | 30/18614 | 1.60E-02 | 0.067564 | 0.030536 | BCL6 | 1 |
| BP | GO:0097421 | liver regeneration | 1月10日 | 30/18614 | 1.60E-02 | 0.067564 | 0.030536 | CEBPB | 1 |
| BP | GO:0045088 | regulation of innate immune response | 2月10日 | 373/18614 | 1.62E-02 | 0.067564 | 0.030536 | FGR/NFKBIA | 2 |
| BP | GO:0030336 | negative regulation of cell migration | 2月10日 | 375/18614 | 1.64E-02 | 0.067564 | 0.030536 | GJA1/KLF4 | 2 |
| BP | GO:0043304 | regulation of mast cell degranulation | 1月10日 | 31/18614 | 1.65E-02 | 0.067564 | 0.030536 | FGR | 1 |
| BP | GO:0070570 | regulation of neuron projection regeneration | 1月10日 | 31/18614 | 1.65E-02 | 0.067564 | 0.030536 | KLF4 | 1 |
| BP | GO:0002696 | positive regulation of leukocyte activation | 2月10日 | 377/18614 | 1.65E-02 | 0.067564 | 0.030536 | FGR/BCL6 | 2 |
| BP | GO:0050863 | regulation of T cell activation | 2月10日 | 377/18614 | 1.65E-02 | 0.067564 | 0.030536 | BCL6/CEBPB | 2 |
| BP | GO:0002313 | mature B cell differentiation involved in immune response | 1月10日 | 32/18614 | 1.71E-02 | 0.067564 | 0.030536 | BCL6 | 1 |
| BP | GO:0002431 | Fc receptor mediated stimulatory signaling pathway | 1月10日 | 32/18614 | 1.71E-02 | 0.067564 | 0.030536 | FGR | 1 |
| BP | GO:0002862 | negative regulation of inflammatory response to antigenic stimulus | 1月10日 | 32/18614 | 1.71E-02 | 0.067564 | 0.030536 | FGR | 1 |
| BP | GO:0008156 | negative regulation of DNA replication | 1月10日 | 32/18614 | 1.71E-02 | 0.067564 | 0.030536 | BCL6 | 1 |
| BP | GO:0010644 | cell communication by electrical coupling | 1月10日 | 32/18614 | 1.71E-02 | 0.067564 | 0.030536 | GJA1 | 1 |
| BP | GO:0010743 | regulation of macrophage derived foam cell differentiation | 1月10日 | 32/18614 | 1.71E-02 | 0.067564 | 0.030536 | NFKBIA | 1 |
| BP | GO:0043552 | positive regulation of phosphatidylinositol 3-kinase activity | 1月10日 | 32/18614 | 1.71E-02 | 0.067564 | 0.030536 | FGR | 1 |
| BP | GO:0002697 | regulation of immune effector process | 2月10日 | 384/18614 | 1.71E-02 | 0.067564 | 0.030536 | FGR/BCL6 | 2 |
| BP | GO:0002828 | regulation of type 2 immune response | 1月10日 | 33/18614 | 1.76E-02 | 0.067564 | 0.030536 | BCL6 | 1 |
| BP | GO:0033006 | regulation of mast cell activation involved in immune response | 1月10日 | 33/18614 | 1.76E-02 | 0.067564 | 0.030536 | FGR | 1 |
| BP | GO:0038094 | Fc-gamma receptor signaling pathway | 1月10日 | 33/18614 | 1.76E-02 | 0.067564 | 0.030536 | FGR | 1 |
| BP | GO:0060795 | cell fate commitment involved in formation of primary germ layer | 1月10日 | 33/18614 | 1.76E-02 | 0.067564 | 0.030536 | KLF4 | 1 |
| BP | GO:2000146 | negative regulation of cell motility | 2月10日 | 390/18614 | 1.76E-02 | 0.067564 | 0.030536 | GJA1/KLF4 | 2 |
| BP | GO:0050867 | positive regulation of cell activation | 2月10日 | 394/18614 | 1.80E-02 | 0.067564 | 0.030536 | FGR/BCL6 | 2 |
| BP | GO:0000132 | establishment of mitotic spindle orientation | 1月10日 | 34/18614 | 1.81E-02 | 0.067564 | 0.030536 | GJA1 | 1 |
| BP | GO:0034405 | response to fluid shear stress | 1月10日 | 34/18614 | 1.81E-02 | 0.067564 | 0.030536 | KLF4 | 1 |
| BP | GO:0045577 | regulation of B cell differentiation | 1月10日 | 34/18614 | 1.81E-02 | 0.067564 | 0.030536 | BCL6 | 1 |
| BP | GO:0050869 | negative regulation of B cell activation | 1月10日 | 34/18614 | 1.81E-02 | 0.067564 | 0.030536 | BCL6 | 1 |
| BP | GO:0051450 | myoblast proliferation | 1月10日 | 34/18614 | 1.81E-02 | 0.067564 | 0.030536 | FGR | 1 |
| BP | GO:0051973 | positive regulation of telomerase activity | 1月10日 | 34/18614 | 1.81E-02 | 0.067564 | 0.030536 | KLF4 | 1 |
| BP | GO:0060563 | neuroepithelial cell differentiation | 1月10日 | 34/18614 | 1.81E-02 | 0.067564 | 0.030536 | CEBPB | 1 |
| BP | GO:2000515 | negative regulation of CD4-positive, alpha-beta T cell activation | 1月10日 | 34/18614 | 1.81E-02 | 0.067564 | 0.030536 | BCL6 | 1 |
| BP | GO:0001701 | in utero embryonic development | 2月10日 | 398/18614 | 1.83E-02 | 0.067564 | 0.030536 | KLF4/CEBPB | 2 |
| BP | GO:0002443 | leukocyte mediated immunity | 2月10日 | 401/18614 | 1.86E-02 | 0.067564 | 0.030536 | FGR/BCL6 | 2 |
| BP | GO:0002719 | negative regulation of cytokine production involved in immune response | 1月10日 | 35/18614 | 1.86E-02 | 0.067564 | 0.030536 | BCL6 | 1 |
| BP | GO:0032633 | interleukin-4 production | 1月10日 | 35/18614 | 1.86E-02 | 0.067564 | 0.030536 | CEBPB | 1 |
| BP | GO:0032673 | regulation of interleukin-4 production | 1月10日 | 35/18614 | 1.86E-02 | 0.067564 | 0.030536 | CEBPB | 1 |
| BP | GO:0035633 | maintenance of blood-brain barrier | 1月10日 | 35/18614 | 1.86E-02 | 0.067564 | 0.030536 | GJA1 | 1 |
| BP | GO:0045786 | negative regulation of cell cycle | 2月10日 | 404/18614 | 1.88E-02 | 0.067564 | 0.030536 | KLF4/BCL6 | 2 |
| BP | GO:0003161 | cardiac conduction system development | 1月10日 | 36/18614 | 1.92E-02 | 0.067564 | 0.030536 | GJA1 | 1 |
| BP | GO:0045589 | regulation of regulatory T cell differentiation | 1月10日 | 36/18614 | 1.92E-02 | 0.067564 | 0.030536 | BCL6 | 1 |
| BP | GO:0048730 | epidermis morphogenesis | 1月10日 | 36/18614 | 1.92E-02 | 0.067564 | 0.030536 | KLF4 | 1 |
| BP | GO:0090218 | positive regulation of lipid kinase activity | 1月10日 | 36/18614 | 1.92E-02 | 0.067564 | 0.030536 | FGR | 1 |
| BP | GO:1905332 | positive regulation of morphogenesis of an epithelium | 1月10日 | 36/18614 | 1.92E-02 | 0.067564 | 0.030536 | GJA1 | 1 |
| BP | GO:0086019 | cell-cell signaling involved in cardiac conduction | 1月10日 | 37/18614 | 1.97E-02 | 0.067564 | 0.030536 | GJA1 | 1 |
| BP | GO:0002335 | mature B cell differentiation | 1月10日 | 38/18614 | 2.02E-02 | 0.067564 | 0.030536 | BCL6 | 1 |
| BP | GO:0010742 | macrophage derived foam cell differentiation | 1月10日 | 38/18614 | 2.02E-02 | 0.067564 | 0.030536 | NFKBIA | 1 |
| BP | GO:0032373 | positive regulation of sterol transport | 1月10日 | 38/18614 | 2.02E-02 | 0.067564 | 0.030536 | NFKBIA | 1 |
| BP | GO:0032376 | positive regulation of cholesterol transport | 1月10日 | 38/18614 | 2.02E-02 | 0.067564 | 0.030536 | NFKBIA | 1 |
| BP | GO:0032717 | negative regulation of interleukin-8 production | 1月10日 | 38/18614 | 2.02E-02 | 0.067564 | 0.030536 | KLF4 | 1 |
| BP | GO:0033260 | nuclear DNA replication | 1月10日 | 38/18614 | 2.02E-02 | 0.067564 | 0.030536 | BCL6 | 1 |
| BP | GO:0043618 | regulation of transcription from RNA polymerase II promoter in response to stress | 1月10日 | 38/18614 | 2.02E-02 | 0.067564 | 0.030536 | CEBPB | 1 |
| BP | GO:0045191 | regulation of isotype switching | 1月10日 | 38/18614 | 2.02E-02 | 0.067564 | 0.030536 | BCL6 | 1 |
| BP | GO:0070229 | negative regulation of lymphocyte apoptotic process | 1月10日 | 38/18614 | 2.02E-02 | 0.067564 | 0.030536 | BCL6 | 1 |
| BP | GO:0001558 | regulation of cell growth | 2月10日 | 420/18614 | 2.03E-02 | 0.067564 | 0.030536 | GJA1/BCL6 | 2 |
| BP | GO:0002757 | immune response-activating signaling pathway | 2月10日 | 423/18614 | 2.05E-02 | 0.067564 | 0.030536 | FGR/NFKBIA | 2 |
| BP | GO:0030279 | negative regulation of ossification | 1月10日 | 39/18614 | 2.08E-02 | 0.067564 | 0.030536 | SRGN | 1 |
| BP | GO:0040001 | establishment of mitotic spindle localization | 1月10日 | 39/18614 | 2.08E-02 | 0.067564 | 0.030536 | GJA1 | 1 |
| BP | GO:0044060 | regulation of endocrine process | 1月10日 | 39/18614 | 2.08E-02 | 0.067564 | 0.030536 | GJA1 | 1 |
| BP | GO:0045066 | regulatory T cell differentiation | 1月10日 | 39/18614 | 2.08E-02 | 0.067564 | 0.030536 | BCL6 | 1 |
| BP | GO:0048821 | erythrocyte development | 1月10日 | 39/18614 | 2.08E-02 | 0.067564 | 0.030536 | BCL6 | 1 |
| BP | GO:0090077 | foam cell differentiation | 1月10日 | 39/18614 | 2.08E-02 | 0.067564 | 0.030536 | NFKBIA | 1 |
| BP | GO:0040013 | negative regulation of locomotion | 2月10日 | 427/18614 | 2.09E-02 | 0.067764 | 0.030626 | GJA1/KLF4 | 2 |
| BP | GO:0001953 | negative regulation of cell-matrix adhesion | 1月10日 | 40/18614 | 2.13E-02 | 0.067767 | 0.030628 | BCL6 | 1 |
| BP | GO:0031063 | regulation of histone deacetylation | 1月10日 | 40/18614 | 2.13E-02 | 0.067767 | 0.030628 | BCL6 | 1 |
| BP | GO:0042092 | type 2 immune response | 1月10日 | 40/18614 | 2.13E-02 | 0.067767 | 0.030628 | BCL6 | 1 |
| BP | GO:1904994 | regulation of leukocyte adhesion to vascular endothelial cell | 1月10日 | 40/18614 | 2.13E-02 | 0.067767 | 0.030628 | KLF4 | 1 |
| BP | GO:0051294 | establishment of spindle orientation | 1月10日 | 41/18614 | 2.18E-02 | 0.069045 | 0.031205 | GJA1 | 1 |
| BP | GO:0031349 | positive regulation of defense response | 2月10日 | 441/18614 | 2.22E-02 | 0.069045 | 0.031205 | NFKBIA/CEBPB | 2 |
| BP | GO:0043551 | regulation of phosphatidylinositol 3-kinase activity | 1月10日 | 42/18614 | 2.23E-02 | 0.069045 | 0.031205 | FGR | 1 |
| BP | GO:0045429 | positive regulation of nitric oxide biosynthetic process | 1月10日 | 42/18614 | 2.23E-02 | 0.069045 | 0.031205 | KLF4 | 1 |
| BP | GO:0045622 | regulation of T-helper cell differentiation | 1月10日 | 42/18614 | 2.23E-02 | 0.069045 | 0.031205 | BCL6 | 1 |
| BP | GO:0051656 | establishment of organelle localization | 2月10日 | 445/18614 | 2.26E-02 | 0.069045 | 0.031205 | GJA1/FGR | 2 |
| BP | GO:0032102 | negative regulation of response to external stimulus | 2月10日 | 446/18614 | 2.27E-02 | 0.069045 | 0.031205 | FGR/KLF4 | 2 |
| BP | GO:0032814 | regulation of natural killer cell activation | 1月10日 | 43/18614 | 2.29E-02 | 0.069045 | 0.031205 | FGR | 1 |
| BP | GO:0034142 | toll-like receptor 4 signaling pathway | 1月10日 | 43/18614 | 2.29E-02 | 0.069045 | 0.031205 | NFKBIA | 1 |
| BP | GO:0044786 | cell cycle DNA replication | 1月10日 | 43/18614 | 2.29E-02 | 0.069045 | 0.031205 | BCL6 | 1 |
| BP | GO:0002764 | immune response-regulating signaling pathway | 2月10日 | 450/18614 | 2.31E-02 | 0.069045 | 0.031205 | FGR/NFKBIA | 2 |
| BP | GO:0001709 | cell fate determination | 1月10日 | 44/18614 | 2.34E-02 | 0.069045 | 0.031205 | KLF4 | 1 |
| BP | GO:0002347 | response to tumor cell | 1月10日 | 44/18614 | 2.34E-02 | 0.069045 | 0.031205 | KLF4 | 1 |
| BP | GO:0033003 | regulation of mast cell activation | 1月10日 | 44/18614 | 2.34E-02 | 0.069045 | 0.031205 | FGR | 1 |
| BP | GO:0043620 | regulation of DNA-templated transcription in response to stress | 1月10日 | 44/18614 | 2.34E-02 | 0.069045 | 0.031205 | CEBPB | 1 |
| BP | GO:0090051 | negative regulation of cell migration involved in sprouting angiogenesis | 1月10日 | 44/18614 | 2.34E-02 | 0.069045 | 0.031205 | KLF4 | 1 |
| BP | GO:1904407 | positive regulation of nitric oxide metabolic process | 1月10日 | 44/18614 | 2.34E-02 | 0.069045 | 0.031205 | KLF4 | 1 |
| BP | GO:1904646 | cellular response to amyloid-beta | 1月10日 | 44/18614 | 2.34E-02 | 0.069045 | 0.031205 | GJA1 | 1 |
| BP | GO:0030890 | positive regulation of B cell proliferation | 1月10日 | 45/18614 | 2.39E-02 | 0.069472 | 0.031398 | BCL6 | 1 |
| BP | GO:0046636 | negative regulation of alpha-beta T cell activation | 1月10日 | 45/18614 | 2.39E-02 | 0.069472 | 0.031398 | BCL6 | 1 |
| BP | GO:0097028 | dendritic cell differentiation | 1月10日 | 45/18614 | 2.39E-02 | 0.069472 | 0.031398 | CEBPB | 1 |
| BP | GO:1903307 | positive regulation of regulated secretory pathway | 1月10日 | 45/18614 | 2.39E-02 | 0.069472 | 0.031398 | FGR | 1 |
| BP | GO:0002831 | regulation of response to biotic stimulus | 2月10日 | 463/18614 | 2.43E-02 | 0.070417 | 0.031825 | FGR/NFKBIA | 2 |
| BP | GO:0045910 | negative regulation of DNA recombination | 1月10日 | 46/18614 | 2.44E-02 | 0.070428 | 0.03183 | BCL6 | 1 |
| BP | GO:0051090 | regulation of DNA-binding transcription factor activity | 2月10日 | 465/18614 | 2.45E-02 | 0.070428 | 0.03183 | NFKBIA/KLF4 | 2 |
| BP | GO:0002701 | negative regulation of production of molecular mediator of immune response | 1月10日 | 47/18614 | 2.50E-02 | 0.070832 | 0.032013 | BCL6 | 1 |
| BP | GO:0002861 | regulation of inflammatory response to antigenic stimulus | 1月10日 | 47/18614 | 2.50E-02 | 0.070832 | 0.032013 | FGR | 1 |
| BP | GO:0051972 | regulation of telomerase activity | 1月10日 | 47/18614 | 2.50E-02 | 0.070832 | 0.032013 | KLF4 | 1 |
| BP | GO:1903131 | mononuclear cell differentiation | 2月10日 | 474/18614 | 2.54E-02 | 0.071487 | 0.032309 | BCL6/CEBPB | 2 |
| BP | GO:0006953 | acute-phase response | 1月10日 | 48/18614 | 2.55E-02 | 0.071487 | 0.032309 | CEBPB | 1 |
| BP | GO:0043300 | regulation of leukocyte degranulation | 1月10日 | 48/18614 | 2.55E-02 | 0.071487 | 0.032309 | FGR | 1 |
| BP | GO:0043330 | response to exogenous dsRNA | 1月10日 | 49/18614 | 2.60E-02 | 0.071902 | 0.032496 | NFKBIA | 1 |
| BP | GO:0046580 | negative regulation of Ras protein signal transduction | 1月10日 | 49/18614 | 2.60E-02 | 0.071902 | 0.032496 | BCL6 | 1 |
| BP | GO:0140895 | cell surface toll-like receptor signaling pathway | 1月10日 | 49/18614 | 2.60E-02 | 0.071902 | 0.032496 | NFKBIA | 1 |
| BP | GO:0045581 | negative regulation of T cell differentiation | 1月10日 | 50/18614 | 2.65E-02 | 0.071902 | 0.032496 | BCL6 | 1 |
| BP | GO:0001819 | positive regulation of cytokine production | 2月10日 | 489/18614 | 2.70E-02 | 0.071902 | 0.032496 | FGR/CEBPB | 2 |
| BP | GO:0043392 | negative regulation of DNA binding | 1月10日 | 51/18614 | 2.71E-02 | 0.071902 | 0.032496 | NFKBIA | 1 |
| BP | GO:0061082 | myeloid leukocyte cytokine production | 1月10日 | 51/18614 | 2.71E-02 | 0.071902 | 0.032496 | BCL6 | 1 |
| BP | GO:1902895 | positive regulation of miRNA transcription | 1月10日 | 51/18614 | 2.71E-02 | 0.071902 | 0.032496 | KLF4 | 1 |
| BP | GO:1904707 | positive regulation of vascular associated smooth muscle cell proliferation | 1月10日 | 51/18614 | 2.71E-02 | 0.071902 | 0.032496 | GJA1 | 1 |
| BP | GO:0019221 | cytokine-mediated signaling pathway | 2月10日 | 492/18614 | 2.73E-02 | 0.071902 | 0.032496 | NFKBIA/CXCL1 | 2 |
| BP | GO:0002253 | activation of immune response | 2月10日 | 495/18614 | 2.76E-02 | 0.071902 | 0.032496 | FGR/NFKBIA | 2 |
| BP | GO:0016049 | cell growth | 2月10日 | 495/18614 | 2.76E-02 | 0.071902 | 0.032496 | GJA1/BCL6 | 2 |
| BP | GO:0031103 | axon regeneration | 1月10日 | 52/18614 | 2.76E-02 | 0.071902 | 0.032496 | KLF4 | 1 |
| BP | GO:2000677 | regulation of transcription regulatory region DNA binding | 1月10日 | 52/18614 | 2.76E-02 | 0.071902 | 0.032496 | KLF4 | 1 |
| BP | GO:0001667 | ameboidal-type cell migration | 2月10日 | 496/18614 | 2.77E-02 | 0.071902 | 0.032496 | GJA1/KLF4 | 2 |
| BP | GO:0010874 | regulation of cholesterol efflux | 1月10日 | 53/18614 | 2.81E-02 | 0.071902 | 0.032496 | NFKBIA | 1 |
| BP | GO:0010883 | regulation of lipid storage | 1月10日 | 53/18614 | 2.81E-02 | 0.071902 | 0.032496 | NFKBIA | 1 |
| BP | GO:0050873 | brown fat cell differentiation | 1月10日 | 53/18614 | 2.81E-02 | 0.071902 | 0.032496 | CEBPB | 1 |
| BP | GO:0070169 | positive regulation of biomineral tissue development | 1月10日 | 53/18614 | 2.81E-02 | 0.071902 | 0.032496 | CEBPB | 1 |
| BP | GO:2000648 | positive regulation of stem cell proliferation | 1月10日 | 53/18614 | 2.81E-02 | 0.071902 | 0.032496 | GJA1 | 1 |
| BP | GO:0002204 | somatic recombination of immunoglobulin genes involved in immune response | 1月10日 | 54/18614 | 2.86E-02 | 0.071902 | 0.032496 | BCL6 | 1 |
| BP | GO:0002208 | somatic diversification of immunoglobulins involved in immune response | 1月10日 | 54/18614 | 2.86E-02 | 0.071902 | 0.032496 | BCL6 | 1 |
| BP | GO:0009409 | response to cold | 1月10日 | 54/18614 | 2.86E-02 | 0.071902 | 0.032496 | NFKBIA | 1 |
| BP | GO:0038093 | Fc receptor signaling pathway | 1月10日 | 54/18614 | 2.86E-02 | 0.071902 | 0.032496 | FGR | 1 |
| BP | GO:0043303 | mast cell degranulation | 1月10日 | 54/18614 | 2.86E-02 | 0.071902 | 0.032496 | FGR | 1 |
| BP | GO:0043550 | regulation of lipid kinase activity | 1月10日 | 54/18614 | 2.86E-02 | 0.071902 | 0.032496 | FGR | 1 |
| BP | GO:0045190 | isotype switching | 1月10日 | 54/18614 | 2.86E-02 | 0.071902 | 0.032496 | BCL6 | 1 |
| BP | GO:0086002 | cardiac muscle cell action potential involved in contraction | 1月10日 | 54/18614 | 2.86E-02 | 0.071902 | 0.032496 | GJA1 | 1 |
| BP | GO:1904645 | response to amyloid-beta | 1月10日 | 54/18614 | 2.86E-02 | 0.071902 | 0.032496 | GJA1 | 1 |
| BP | GO:0043370 | regulation of CD4-positive, alpha-beta T cell differentiation | 1月10日 | 55/18614 | 2.92E-02 | 0.071902 | 0.032496 | BCL6 | 1 |
| BP | GO:2000772 | regulation of cellular senescence | 1月10日 | 55/18614 | 2.92E-02 | 0.071902 | 0.032496 | BCL6 | 1 |
| BP | GO:0002279 | mast cell activation involved in immune response | 1月10日 | 56/18614 | 2.97E-02 | 0.071902 | 0.032496 | FGR | 1 |
| BP | GO:0002707 | negative regulation of lymphocyte mediated immunity | 1月10日 | 56/18614 | 2.97E-02 | 0.071902 | 0.032496 | BCL6 | 1 |
| BP | GO:0002752 | cell surface pattern recognition receptor signaling pathway | 1月10日 | 56/18614 | 2.97E-02 | 0.071902 | 0.032496 | NFKBIA | 1 |
| BP | GO:0002823 | negative regulation of adaptive immune response based on somatic recombination of immune receptors built from immunoglobulin superfamily domains | 1月10日 | 56/18614 | 2.97E-02 | 0.071902 | 0.032496 | BCL6 | 1 |
| BP | GO:0042908 | xenobiotic transport | 1月10日 | 56/18614 | 2.97E-02 | 0.071902 | 0.032496 | GJA1 | 1 |
| BP | GO:0043331 | response to dsRNA | 1月10日 | 56/18614 | 2.97E-02 | 0.071902 | 0.032496 | NFKBIA | 1 |
| BP | GO:0051058 | negative regulation of small GTPase mediated signal transduction | 1月10日 | 56/18614 | 2.97E-02 | 0.071902 | 0.032496 | BCL6 | 1 |
| BP | GO:0051293 | establishment of spindle localization | 1月10日 | 56/18614 | 2.97E-02 | 0.071902 | 0.032496 | GJA1 | 1 |
| BP | GO:0060986 | endocrine hormone secretion | 1月10日 | 56/18614 | 2.97E-02 | 0.071902 | 0.032496 | GJA1 | 1 |
| BP | GO:2000107 | negative regulation of leukocyte apoptotic process | 1月10日 | 56/18614 | 2.97E-02 | 0.071902 | 0.032496 | BCL6 | 1 |
| BP | GO:0001541 | ovarian follicle development | 1月10日 | 57/18614 | 3.02E-02 | 0.071973 | 0.032528 | CEBPB | 1 |
| BP | GO:0002448 | mast cell mediated immunity | 1月10日 | 57/18614 | 3.02E-02 | 0.071973 | 0.032528 | FGR | 1 |
| BP | GO:0007566 | embryo implantation | 1月10日 | 57/18614 | 3.02E-02 | 0.071973 | 0.032528 | GJA1 | 1 |
| BP | GO:0043388 | positive regulation of DNA binding | 1月10日 | 57/18614 | 3.02E-02 | 0.071973 | 0.032528 | KLF4 | 1 |
| BP | GO:0061756 | leukocyte adhesion to vascular endothelial cell | 1月10日 | 57/18614 | 3.02E-02 | 0.071973 | 0.032528 | KLF4 | 1 |
| BP | GO:0090311 | regulation of protein deacetylation | 1月10日 | 58/18614 | 3.07E-02 | 0.072742 | 0.032876 | BCL6 | 1 |
| BP | GO:0090329 | regulation of DNA-templated DNA replication | 1月10日 | 58/18614 | 3.07E-02 | 0.072742 | 0.032876 | BCL6 | 1 |
| BP | GO:0031102 | neuron projection regeneration | 1月10日 | 59/18614 | 3.13E-02 | 0.073501 | 0.033219 | KLF4 | 1 |
| BP | GO:0070228 | regulation of lymphocyte apoptotic process | 1月10日 | 59/18614 | 3.13E-02 | 0.073501 | 0.033219 | BCL6 | 1 |
| BP | GO:0002886 | regulation of myeloid leukocyte mediated immunity | 1月10日 | 60/18614 | 3.18E-02 | 0.073777 | 0.033344 | FGR | 1 |
| BP | GO:0045620 | negative regulation of lymphocyte differentiation | 1月10日 | 60/18614 | 3.18E-02 | 0.073777 | 0.033344 | BCL6 | 1 |
| BP | GO:1903670 | regulation of sprouting angiogenesis | 1月10日 | 60/18614 | 3.18E-02 | 0.073777 | 0.033344 | KLF4 | 1 |
| BP | GO:2000630 | positive regulation of miRNA metabolic process | 1月10日 | 60/18614 | 3.18E-02 | 0.073777 | 0.033344 | KLF4 | 1 |
| BP | GO:0002820 | negative regulation of adaptive immune response | 1月10日 | 61/18614 | 3.23E-02 | 0.073813 | 0.03336 | BCL6 | 1 |
| BP | GO:0015800 | acidic amino acid transport | 1月10日 | 61/18614 | 3.23E-02 | 0.073813 | 0.03336 | GJA1 | 1 |
| BP | GO:0034113 | heterotypic cell-cell adhesion | 1月10日 | 61/18614 | 3.23E-02 | 0.073813 | 0.03336 | KLF4 | 1 |
| BP | GO:0051653 | spindle localization | 1月10日 | 61/18614 | 3.23E-02 | 0.073813 | 0.03336 | GJA1 | 1 |
| BP | GO:0086065 | cell communication involved in cardiac conduction | 1月10日 | 61/18614 | 3.23E-02 | 0.073813 | 0.03336 | GJA1 | 1 |
| BP | GO:0016447 | somatic recombination of immunoglobulin gene segments | 1月10日 | 62/18614 | 3.28E-02 | 0.074306 | 0.033583 | BCL6 | 1 |
| BP | GO:0045428 | regulation of nitric oxide biosynthetic process | 1月10日 | 62/18614 | 3.28E-02 | 0.074306 | 0.033583 | KLF4 | 1 |
| BP | GO:0060261 | positive regulation of transcription initiation by RNA polymerase II | 1月10日 | 62/18614 | 3.28E-02 | 0.074306 | 0.033583 | NFKBIA | 1 |
| BP | GO:0002712 | regulation of B cell mediated immunity | 1月10日 | 63/18614 | 3.33E-02 | 0.074561 | 0.033698 | BCL6 | 1 |
| BP | GO:0002889 | regulation of immunoglobulin mediated immune response | 1月10日 | 63/18614 | 3.33E-02 | 0.074561 | 0.033698 | BCL6 | 1 |
| BP | GO:0033363 | secretory granule organization | 1月10日 | 63/18614 | 3.33E-02 | 0.074561 | 0.033698 | SRGN | 1 |
| BP | GO:0070059 | intrinsic apoptotic signaling pathway in response to endoplasmic reticulum stress | 1月10日 | 63/18614 | 3.33E-02 | 0.074561 | 0.033698 | CEBPB | 1 |
| BP | GO:1905953 | negative regulation of lipid localization | 1月10日 | 64/18614 | 3.39E-02 | 0.075274 | 0.03402 | NFKBIA | 1 |
| BP | GO:0030888 | regulation of B cell proliferation | 1月10日 | 65/18614 | 3.44E-02 | 0.075274 | 0.03402 | BCL6 | 1 |
| BP | GO:0031663 | lipopolysaccharide-mediated signaling pathway | 1月10日 | 65/18614 | 3.44E-02 | 0.075274 | 0.03402 | NFKBIA | 1 |
| BP | GO:0032507 | maintenance of protein location in cell | 1月10日 | 65/18614 | 3.44E-02 | 0.075274 | 0.03402 | SRGN | 1 |
| BP | GO:0046888 | negative regulation of hormone secretion | 1月10日 | 65/18614 | 3.44E-02 | 0.075274 | 0.03402 | GJA1 | 1 |
| BP | GO:0080164 | regulation of nitric oxide metabolic process | 1月10日 | 65/18614 | 3.44E-02 | 0.075274 | 0.03402 | KLF4 | 1 |
| BP | GO:1905330 | regulation of morphogenesis of an epithelium | 1月10日 | 65/18614 | 3.44E-02 | 0.075274 | 0.03402 | GJA1 | 1 |
| BP | GO:0010812 | negative regulation of cell-substrate adhesion | 1月10日 | 66/18614 | 3.49E-02 | 0.075958 | 0.034329 | BCL6 | 1 |
| BP | GO:0031100 | animal organ regeneration | 1月10日 | 66/18614 | 3.49E-02 | 0.075958 | 0.034329 | CEBPB | 1 |
| BP | GO:0002704 | negative regulation of leukocyte mediated immunity | 1月10日 | 68/18614 | 3.59E-02 | 0.076621 | 0.034629 | BCL6 | 1 |
| BP | GO:0016445 | somatic diversification of immunoglobulins | 1月10日 | 68/18614 | 3.59E-02 | 0.076621 | 0.034629 | BCL6 | 1 |
| BP | GO:0035019 | somatic stem cell population maintenance | 1月10日 | 68/18614 | 3.59E-02 | 0.076621 | 0.034629 | KLF4 | 1 |
| BP | GO:0045670 | regulation of osteoclast differentiation | 1月10日 | 68/18614 | 3.59E-02 | 0.076621 | 0.034629 | CEBPB | 1 |
| BP | GO:0061180 | mammary gland epithelium development | 1月10日 | 68/18614 | 3.59E-02 | 0.076621 | 0.034629 | CEBPB | 1 |
| BP | GO:1902893 | regulation of miRNA transcription | 1月10日 | 68/18614 | 3.59E-02 | 0.076621 | 0.034629 | KLF4 | 1 |
| BP | GO:2000144 | positive regulation of DNA-templated transcription initiation | 1月10日 | 68/18614 | 3.59E-02 | 0.076621 | 0.034629 | NFKBIA | 1 |
| BP | GO:0042093 | T-helper cell differentiation | 1月10日 | 69/18614 | 3.65E-02 | 0.077053 | 0.034824 | BCL6 | 1 |
| BP | GO:0045576 | mast cell activation | 1月10日 | 69/18614 | 3.65E-02 | 0.077053 | 0.034824 | FGR | 1 |
| BP | GO:0061614 | miRNA transcription | 1月10日 | 69/18614 | 3.65E-02 | 0.077053 | 0.034824 | KLF4 | 1 |
| BP | GO:0002294 | CD4-positive, alpha-beta T cell differentiation involved in immune response | 1月10日 | 71/18614 | 3.75E-02 | 0.078116 | 0.035305 | BCL6 | 1 |
| BP | GO:0002562 | somatic diversification of immune receptors via germline recombination within a single locus | 1月10日 | 71/18614 | 3.75E-02 | 0.078116 | 0.035305 | BCL6 | 1 |
| BP | GO:0016444 | somatic cell DNA recombination | 1月10日 | 71/18614 | 3.75E-02 | 0.078116 | 0.035305 | BCL6 | 1 |
| BP | GO:0033344 | cholesterol efflux | 1月10日 | 71/18614 | 3.75E-02 | 0.078116 | 0.035305 | NFKBIA | 1 |
| BP | GO:0045600 | positive regulation of fat cell differentiation | 1月10日 | 71/18614 | 3.75E-02 | 0.078116 | 0.035305 | CEBPB | 1 |
| BP | GO:0002287 | alpha-beta T cell activation involved in immune response | 1月10日 | 72/18614 | 3.80E-02 | 0.078302 | 0.035389 | BCL6 | 1 |
| BP | GO:0002293 | alpha-beta T cell differentiation involved in immune response | 1月10日 | 72/18614 | 3.80E-02 | 0.078302 | 0.035389 | BCL6 | 1 |
| BP | GO:0042130 | negative regulation of T cell proliferation | 1月10日 | 72/18614 | 3.80E-02 | 0.078302 | 0.035389 | CEBPB | 1 |
| BP | GO:0060260 | regulation of transcription initiation by RNA polymerase II | 1月10日 | 72/18614 | 3.80E-02 | 0.078302 | 0.035389 | NFKBIA | 1 |
| BP | GO:0046637 | regulation of alpha-beta T cell differentiation | 1月10日 | 73/18614 | 3.85E-02 | 0.079147 | 0.035771 | BCL6 | 1 |
| BP | GO:0002067 | glandular epithelial cell differentiation | 1月10日 | 74/18614 | 3.91E-02 | 0.079762 | 0.036049 | KLF4 | 1 |
| BP | GO:2000573 | positive regulation of DNA biosynthetic process | 1月10日 | 74/18614 | 3.91E-02 | 0.079762 | 0.036049 | KLF4 | 1 |
| BP | GO:0002381 | immunoglobulin production involved in immunoglobulin-mediated immune response | 1月10日 | 75/18614 | 3.96E-02 | 0.08037 | 0.036324 | BCL6 | 1 |
| BP | GO:2000242 | negative regulation of reproductive process | 1月10日 | 75/18614 | 3.96E-02 | 0.08037 | 0.036324 | GJA1 | 1 |
| BP | GO:0002637 | regulation of immunoglobulin production | 1月10日 | 76/18614 | 4.01E-02 | 0.080681 | 0.036464 | BCL6 | 1 |
| BP | GO:0086003 | cardiac muscle cell contraction | 1月10日 | 76/18614 | 4.01E-02 | 0.080681 | 0.036464 | GJA1 | 1 |
| BP | GO:0006809 | nitric oxide biosynthetic process | 1月10日 | 77/18614 | 4.06E-02 | 0.080681 | 0.036464 | KLF4 | 1 |
| BP | GO:0050853 | B cell receptor signaling pathway | 1月10日 | 77/18614 | 4.06E-02 | 0.080681 | 0.036464 | NFKBIA | 1 |
| BP | GO:2000514 | regulation of CD4-positive, alpha-beta T cell activation | 1月10日 | 77/18614 | 4.06E-02 | 0.080681 | 0.036464 | BCL6 | 1 |
| BP | GO:0002200 | somatic diversification of immune receptors | 1月10日 | 78/18614 | 4.11E-02 | 0.080681 | 0.036464 | BCL6 | 1 |
| BP | GO:0002220 | innate immune response activating cell surface receptor signaling pathway | 1月10日 | 78/18614 | 4.11E-02 | 0.080681 | 0.036464 | NFKBIA | 1 |
| BP | GO:0002437 | inflammatory response to antigenic stimulus | 1月10日 | 78/18614 | 4.11E-02 | 0.080681 | 0.036464 | FGR | 1 |
| BP | GO:0043154 | negative regulation of cysteine-type endopeptidase activity involved in apoptotic process | 1月10日 | 78/18614 | 4.11E-02 | 0.080681 | 0.036464 | KLF4 | 1 |
| BP | GO:0032371 | regulation of sterol transport | 1月10日 | 79/18614 | 4.16E-02 | 0.080681 | 0.036464 | NFKBIA | 1 |
| BP | GO:0032374 | regulation of cholesterol transport | 1月10日 | 79/18614 | 4.16E-02 | 0.080681 | 0.036464 | NFKBIA | 1 |
| BP | GO:0086001 | cardiac muscle cell action potential | 1月10日 | 79/18614 | 4.16E-02 | 0.080681 | 0.036464 | GJA1 | 1 |
| BP | GO:0002292 | T cell differentiation involved in immune response | 1月10日 | 80/18614 | 4.22E-02 | 0.080681 | 0.036464 | BCL6 | 1 |
| BP | GO:0007193 | adenylate cyclase-inhibiting G protein-coupled receptor signaling pathway | 1月10日 | 80/18614 | 4.22E-02 | 0.080681 | 0.036464 | RGS1 | 1 |
| BP | GO:0030500 | regulation of bone mineralization | 1月10日 | 80/18614 | 4.22E-02 | 0.080681 | 0.036464 | SRGN | 1 |
| BP | GO:0043537 | negative regulation of blood vessel endothelial cell migration | 1月10日 | 80/18614 | 4.22E-02 | 0.080681 | 0.036464 | KLF4 | 1 |
| BP | GO:0045921 | positive regulation of exocytosis | 1月10日 | 80/18614 | 4.22E-02 | 0.080681 | 0.036464 | FGR | 1 |
| BP | GO:0070227 | lymphocyte apoptotic process | 1月10日 | 80/18614 | 4.22E-02 | 0.080681 | 0.036464 | BCL6 | 1 |
| BP | GO:0071230 | cellular response to amino acid stimulus | 1月10日 | 80/18614 | 4.22E-02 | 0.080681 | 0.036464 | CEBPB | 1 |
| BP | GO:0090049 | regulation of cell migration involved in sprouting angiogenesis | 1月10日 | 80/18614 | 4.22E-02 | 0.080681 | 0.036464 | KLF4 | 1 |
| BP | GO:0140115 | export across plasma membrane | 1月10日 | 80/18614 | 4.22E-02 | 0.080681 | 0.036464 | GJA1 | 1 |
| BP | GO:2000142 | regulation of DNA-templated transcription initiation | 1月10日 | 80/18614 | 4.22E-02 | 0.080681 | 0.036464 | NFKBIA | 1 |
| BP | GO:0043299 | leukocyte degranulation | 1月10日 | 81/18614 | 4.27E-02 | 0.081243 | 0.036718 | FGR | 1 |
| BP | GO:2000628 | regulation of miRNA metabolic process | 1月10日 | 81/18614 | 4.27E-02 | 0.081243 | 0.036718 | KLF4 | 1 |
| BP | GO:0061844 | antimicrobial humoral immune response mediated by antimicrobial peptide | 1月10日 | 82/18614 | 4.32E-02 | 0.082012 | 0.037066 | CXCL1 | 1 |
| BP | GO:0014068 | positive regulation of phosphatidylinositol 3-kinase signaling | 1月10日 | 83/18614 | 4.37E-02 | 0.082137 | 0.037122 | FGR | 1 |
| BP | GO:0035023 | regulation of Rho protein signal transduction | 1月10日 | 83/18614 | 4.37E-02 | 0.082137 | 0.037122 | BCL6 | 1 |
| BP | GO:0046209 | nitric oxide metabolic process | 1月10日 | 83/18614 | 4.37E-02 | 0.082137 | 0.037122 | KLF4 | 1 |
| BP | GO:0050871 | positive regulation of B cell activation | 1月10日 | 83/18614 | 4.37E-02 | 0.082137 | 0.037122 | BCL6 | 1 |
| BP | GO:2001057 | reactive nitrogen species metabolic process | 1月10日 | 84/18614 | 4.42E-02 | 0.082893 | 0.037464 | KLF4 | 1 |
| BP | GO:0019915 | lipid storage | 1月10日 | 86/18614 | 4.53E-02 | 0.083962 | 0.037947 | NFKBIA | 1 |
| BP | GO:0032418 | lysosome localization | 1月10日 | 86/18614 | 4.53E-02 | 0.083962 | 0.037947 | FGR | 1 |
| BP | GO:1990849 | vacuolar localization | 1月10日 | 86/18614 | 4.53E-02 | 0.083962 | 0.037947 | FGR | 1 |
| BP | GO:2000134 | negative regulation of G1/S transition of mitotic cell cycle | 1月10日 | 86/18614 | 4.53E-02 | 0.083962 | 0.037947 | KLF4 | 1 |
| BP | GO:0032370 | positive regulation of lipid transport | 1月10日 | 87/18614 | 4.58E-02 | 0.084488 | 0.038185 | NFKBIA | 1 |
| BP | GO:0048678 | response to axon injury | 1月10日 | 87/18614 | 4.58E-02 | 0.084488 | 0.038185 | KLF4 | 1 |
| BP | GO:0031058 | positive regulation of histone modification | 1月10日 | 88/18614 | 4.63E-02 | 0.084674 | 0.038269 | BCL6 | 1 |
| BP | GO:0046849 | bone remodeling | 1月10日 | 88/18614 | 4.63E-02 | 0.084674 | 0.038269 | GJA1 | 1 |
| BP | GO:0050672 | negative regulation of lymphocyte proliferation | 1月10日 | 88/18614 | 4.63E-02 | 0.084674 | 0.038269 | CEBPB | 1 |
| BP | GO:0001892 | embryonic placenta development | 1月10日 | 89/18614 | 4.68E-02 | 0.084674 | 0.038269 | CEBPB | 1 |
| BP | GO:0002312 | B cell activation involved in immune response | 1月10日 | 89/18614 | 4.68E-02 | 0.084674 | 0.038269 | BCL6 | 1 |
| BP | GO:0032945 | negative regulation of mononuclear cell proliferation | 1月10日 | 89/18614 | 4.68E-02 | 0.084674 | 0.038269 | CEBPB | 1 |
| BP | GO:0043367 | CD4-positive, alpha-beta T cell differentiation | 1月10日 | 89/18614 | 4.68E-02 | 0.084674 | 0.038269 | BCL6 | 1 |
| BP | GO:0071229 | cellular response to acid chemical | 1月10日 | 89/18614 | 4.68E-02 | 0.084674 | 0.038269 | CEBPB | 1 |
| BP | GO:2000106 | regulation of leukocyte apoptotic process | 1月10日 | 90/18614 | 4.73E-02 | 0.085393 | 0.038594 | BCL6 | 1 |
| BP | GO:0070098 | chemokine-mediated signaling pathway | 1月10日 | 91/18614 | 4.78E-02 | 0.085685 | 0.038725 | CXCL1 | 1 |
| BP | GO:0072091 | regulation of stem cell proliferation | 1月10日 | 91/18614 | 4.78E-02 | 0.085685 | 0.038725 | GJA1 | 1 |
| BP | GO:2000117 | negative regulation of cysteine-type endopeptidase activity | 1月10日 | 91/18614 | 4.78E-02 | 0.085685 | 0.038725 | KLF4 | 1 |
| BP | GO:0009791 | post-embryonic development | 1月10日 | 92/18614 | 4.84E-02 | 0.085972 | 0.038855 | KLF4 | 1 |
| BP | GO:0045666 | positive regulation of neuron differentiation | 1月10日 | 92/18614 | 4.84E-02 | 0.085972 | 0.038855 | BCL6 | 1 |
| BP | GO:0060761 | negative regulation of response to cytokine stimulus | 1月10日 | 92/18614 | 4.84E-02 | 0.085972 | 0.038855 | KLF4 | 1 |
| BP | GO:0050886 | endocrine process | 1月10日 | 93/18614 | 4.89E-02 | 0.086674 | 0.039172 | GJA1 | 1 |
| BP | GO:0006835 | dicarboxylic acid transport | 1月10日 | 94/18614 | 4.94E-02 | 0.087161 | 0.039392 | GJA1 | 1 |
| BP | GO:0016575 | histone deacetylation | 1月10日 | 94/18614 | 4.94E-02 | 0.087161 | 0.039392 | BCL6 | 1 |
| BP | GO:0002753 | cytosolic pattern recognition receptor signaling pathway | 1月10日 | 95/18614 | 4.99E-02 | 0.087431 | 0.039515 | NFKBIA | 1 |
| BP | GO:0045638 | negative regulation of myeloid cell differentiation | 1月10日 | 95/18614 | 4.99E-02 | 0.087431 | 0.039515 | NFKBIA | 1 |
| BP | GO:1904705 | regulation of vascular associated smooth muscle cell proliferation | 1月10日 | 95/18614 | 4.99E-02 | 0.087431 | 0.039515 | GJA1 | 1 |
| CC | GO:0034774 | secretory granule lumen | 3月10日 | 322/19518 | 4.90E-04 | 0.008198 | 0.003955 | SRGN/FGR/CXCL1 | 3 |
| CC | GO:0060205 | cytoplasmic vesicle lumen | 3月10日 | 325/19518 | 5.03E-04 | 0.008198 | 0.003955 | SRGN/FGR/CXCL1 | 3 |
| CC | GO:0031983 | vesicle lumen | 3月10日 | 327/19518 | 5.12E-04 | 0.008198 | 0.003955 | SRGN/FGR/CXCL1 | 3 |
| CC | GO:0031234 | extrinsic component of cytoplasmic side of plasma membrane | 2月10日 | 80/19518 | 7.31E-04 | 0.00877 | 0.004231 | RGS1/FGR | 2 |
| CC | GO:0019897 | extrinsic component of plasma membrane | 2月10日 | 147/19518 | 2.44E-03 | 0.022744 | 0.010973 | RGS1/FGR | 2 |
| CC | GO:0009898 | cytoplasmic side of plasma membrane | 2月10日 | 159/19518 | 2.84E-03 | 0.022744 | 0.010973 | RGS1/FGR | 2 |
| CC | GO:0098562 | cytoplasmic side of membrane | 2月10日 | 193/19518 | 4.15E-03 | 0.02849 | 0.013745 | RGS1/FGR | 2 |
| CC | GO:0044194 | cytolytic granule | 1月10日 | 13/19518 | 6.64E-03 | 0.037791 | 0.018233 | SRGN | 1 |
| CC | GO:0090575 | RNA polymerase II transcription regulator complex | 2月10日 | 254/19518 | 7.09E-03 | 0.037791 | 0.018233 | CEBPD/CEBPB | 2 |
| CC | GO:0019898 | extrinsic component of membrane | 2月10日 | 288/19518 | 9.03E-03 | 0.043342 | 0.020911 | RGS1/FGR | 2 |
| CC | GO:0005922 | connexin complex | 1月10日 | 21/19518 | 1.07E-02 | 0.046734 | 0.022547 | GJA1 | 1 |
| CC | GO:0005921 | gap junction | 1月10日 | 32/19518 | 1.63E-02 | 0.065114 | 0.031414 | GJA1 | 1 |
| CC | GO:0016235 | aggresome | 1月10日 | 36/19518 | 1.83E-02 | 0.067556 | 0.032593 | FGR | 1 |
| CC | GO:0014704 | intercalated disc | 1月10日 | 50/19518 | 2.53E-02 | 0.081143 | 0.039148 | GJA1 | 1 |
| CC | GO:1904724 | tertiary granule lumen | 1月10日 | 55/19518 | 2.78E-02 | 0.081143 | 0.039148 | CXCL1 | 1 |
| CC | GO:0030660 | Golgi-associated vesicle membrane | 1月10日 | 58/19518 | 2.93E-02 | 0.081143 | 0.039148 | GJA1 | 1 |
| CC | GO:0000791 | euchromatin | 1月10日 | 60/19518 | 3.03E-02 | 0.081143 | 0.039148 | KLF4 | 1 |
| CC | GO:0035580 | specific granule lumen | 1月10日 | 62/19518 | 3.13E-02 | 0.081143 | 0.039148 | CXCL1 | 1 |
| CC | GO:0005657 | replication fork | 1月10日 | 66/19518 | 3.33E-02 | 0.081143 | 0.039148 | BCL6 | 1 |
| CC | GO:0031093 | platelet alpha granule lumen | 1月10日 | 67/19518 | 3.38E-02 | 0.081143 | 0.039148 | SRGN | 1 |
| CC | GO:0044291 | cell-cell contact zone | 1月10日 | 72/19518 | 3.63E-02 | 0.081342 | 0.039244 | GJA1 | 1 |
| CC | GO:0016234 | inclusion body | 1月10日 | 74/19518 | 3.73E-02 | 0.081342 | 0.039244 | FGR | 1 |
| CC | GO:0005758 | mitochondrial intermembrane space | 1月10日 | 84/19518 | 4.22E-02 | 0.087029 | 0.041988 | FGR | 1 |
| CC | GO:0005798 | Golgi-associated vesicle | 1月10日 | 91/19518 | 4.57E-02 | 0.087029 | 0.041988 | GJA1 | 1 |
| CC | GO:0031091 | platelet alpha granule | 1月10日 | 91/19518 | 4.57E-02 | 0.087029 | 0.041988 | SRGN | 1 |
| CC | GO:0031970 | organelle envelope lumen | 1月10日 | 94/19518 | 4.71E-02 | 0.087029 | 0.041988 | FGR | 1 |
| MF | GO:0031490 | chromatin DNA binding | 3月10日 | 118/18369 | 3.00E-05 | 0.00162 | 0.000726 | KLF4/BCL6/CEBPB | 3 |
| MF | GO:0140297 | DNA-binding transcription factor binding | 4月10日 | 478/18369 | 8.39E-05 | 0.002267 | 0.001016 | NFKBIA/KLF4/BCL6/CEBPB | 4 |
| MF | GO:0061629 | RNA polymerase II-specific DNA-binding transcription factor binding | 3月10日 | 348/18369 | 7.33E-04 | 0.01319 | 0.005914 | NFKBIA/KLF4/CEBPB | 3 |
| MF | GO:0008013 | beta-catenin binding | 2月10日 | 88/18369 | 9.96E-04 | 0.013445 | 0.006028 | GJA1/KLF4 | 2 |
| MF | GO:0001228 | DNA-binding transcription activator activity, RNA polymerase II-specific | 3月10日 | 468/18369 | 1.73E-03 | 0.015912 | 0.007134 | CEBPD/KLF4/CEBPB | 3 |
| MF | GO:0001216 | DNA-binding transcription activator activity | 3月10日 | 472/18369 | 1.77E-03 | 0.015912 | 0.007134 | CEBPD/KLF4/CEBPB | 3 |
| MF | GO:0034987 | immunoglobulin receptor binding | 1月10日 | 10/18369 | 5.43E-03 | 0.037361 | 0.016751 | FGR | 1 |
| MF | GO:0001161 | intronic transcription regulatory region sequence-specific DNA binding | 1月10日 | 11/18369 | 5.97E-03 | 0.037361 | 0.016751 | BCL6 | 1 |
| MF | GO:0005243 | gap junction channel activity | 1月10日 | 12/18369 | 6.52E-03 | 0.037361 | 0.016751 | GJA1 | 1 |
| MF | GO:0035259 | nuclear glucocorticoid receptor binding | 1月10日 | 13/18369 | 7.06E-03 | 0.037361 | 0.016751 | CEBPB | 1 |
| MF | GO:0015562 | efflux transmembrane transporter activity | 1月10日 | 15/18369 | 8.14E-03 | 0.037361 | 0.016751 | GJA1 | 1 |
| MF | GO:0045236 | CXCR chemokine receptor binding | 1月10日 | 18/18369 | 9.76E-03 | 0.037361 | 0.016751 | CXCL1 | 1 |
| MF | GO:0000979 | RNA polymerase II core promoter sequence-specific DNA binding | 1月10日 | 19/18369 | 1.03E-02 | 0.037361 | 0.016751 | CEBPB | 1 |
| MF | GO:0022829 | wide pore channel activity | 1月10日 | 19/18369 | 1.03E-02 | 0.037361 | 0.016751 | GJA1 | 1 |
| MF | GO:0001227 | DNA-binding transcription repressor activity, RNA polymerase II-specific | 2月10日 | 303/18369 | 1.12E-02 | 0.037361 | 0.016751 | BCL6/CEBPB | 2 |
| MF | GO:0001217 | DNA-binding transcription repressor activity | 2月10日 | 308/18369 | 1.15E-02 | 0.037361 | 0.016751 | BCL6/CEBPB | 2 |
| MF | GO:0044389 | ubiquitin-like protein ligase binding | 2月10日 | 320/18369 | 1.24E-02 | 0.037361 | 0.016751 | NFKBIA/CEBPB | 2 |
| MF | GO:0035035 | histone acetyltransferase binding | 1月10日 | 23/18369 | 1.25E-02 | 0.037361 | 0.016751 | CEBPB | 1 |
| MF | GO:0001965 | G-protein alpha-subunit binding | 1月10日 | 26/18369 | 1.41E-02 | 0.037983 | 0.01703 | RGS1 | 1 |
| MF | GO:0008139 | nuclear localization sequence binding | 1月10日 | 26/18369 | 1.41E-02 | 0.037983 | 0.01703 | NFKBIA | 1 |
| MF | GO:0051059 | NF-kappaB binding | 1月10日 | 31/18369 | 1.68E-02 | 0.043078 | 0.019314 | NFKBIA | 1 |
| MF | GO:0001046 | core promoter sequence-specific DNA binding | 1月10日 | 43/18369 | 2.32E-02 | 0.052728 | 0.02364 | CEBPB | 1 |
| MF | GO:0004715 | non-membrane spanning protein tyrosine kinase activity | 1月10日 | 43/18369 | 2.32E-02 | 0.052728 | 0.02364 | FGR | 1 |
| MF | GO:0001222 | transcription corepressor binding | 1月10日 | 45/18369 | 2.42E-02 | 0.052728 | 0.02364 | BCL6 | 1 |
| MF | GO:0001784 | phosphotyrosine residue binding | 1月10日 | 47/18369 | 2.53E-02 | 0.052728 | 0.02364 | FGR | 1 |
| MF | GO:0005048 | signal sequence binding | 1月10日 | 48/18369 | 2.58E-02 | 0.052728 | 0.02364 | NFKBIA | 1 |
| MF | GO:0008009 | chemokine activity | 1月10日 | 49/18369 | 2.64E-02 | 0.052728 | 0.02364 | CXCL1 | 1 |
| MF | GO:0045309 | protein phosphorylated amino acid binding | 1月10日 | 58/18369 | 3.11E-02 | 0.060051 | 0.026923 | FGR | 1 |
| MF | GO:1990841 | promoter-specific chromatin binding | 1月10日 | 63/18369 | 3.38E-02 | 0.062902 | 0.028202 | KLF4 | 1 |
| MF | GO:0042379 | chemokine receptor binding | 1月10日 | 74/18369 | 3.96E-02 | 0.07123 | 0.031936 | CXCL1 | 1 |
| MF | GO:0051219 | phosphoprotein binding | 1月10日 | 92/18369 | 4.90E-02 | 0.085324 | 0.038254 | FGR | 1 |

Supplementary Table 6. The GO results of keygenes.
