# Supplementary material for: Exploring the pathogenesis and key genes associated of acute myocardial infarction complicated with Alzheimer’s disease
Source: Sci Rep. 2024 Jan 16;14:1449. doi: 10.1038/s41598-024-52094-4 (PMC10791667; doi:10.1038/s41598-024-52094-4)
Supplement: Supplementary file 7 — Supplementary Table 7. [file 41598_2024_52094_MOESM7_ESM.docx]

| ID | Description | GeneRatio | BgRatio | pvalue | p.adjust | qvalue | geneID | Count |
| --- | --- | --- | --- | --- | --- | --- | --- | --- |
| hsa04657 | IL-17 signaling pathway | 3月7日 | 94/8464 | 4.50E-05 | 2.32E-03 | 0.001939 | NFKBIA/CXCL1/CEBPB | 3 |
| hsa04668 | TNF signaling pathway | 3月7日 | 114/8464 | 8.01E-05 | 2.32E-03 | 0.001939 | NFKBIA/CXCL1/CEBPB | 3 |
| hsa04062 | Chemokine signaling pathway | 3月7日 | 192/8464 | 3.76E-04 | 7.27E-03 | 0.00607 | FGR/NFKBIA/CXCL1 | 3 |
| hsa05134 | Legionellosis | 2月7日 | 57/8464 | 9.16E-04 | 1.33E-02 | 0.011085 | NFKBIA/CXCL1 | 2 |
| hsa05120 | Epithelial cell signaling in Helicobacter pylori infection | 2月7日 | 70/8464 | 1.38E-03 | 1.60E-02 | 0.01335 | NFKBIA/CXCL1 | 2 |
| hsa04064 | NF-kappa B signaling pathway | 2月7日 | 104/8464 | 3.02E-03 | 2.92E-02 | 0.024344 | NFKBIA/CXCL1 | 2 |
| hsa04936 | Alcoholic liver disease | 2月7日 | 142/8464 | 5.55E-03 | 4.60E-02 | 0.038419 | NFKBIA/CXCL1 | 2 |
| hsa04621 | NOD-like receptor signaling pathway | 2月7日 | 186/8464 | 9.38E-03 | 5.90E-02 | 0.049265 | NFKBIA/CXCL1 | 2 |
| hsa05202 | Transcriptional misregulation in cancer | 2月7日 | 193/8464 | 1.01E-02 | 5.90E-02 | 0.049265 | BCL6/CEBPB | 2 |
| hsa05167 | Kaposi sarcoma-associated herpesvirus infection | 2月7日 | 194/8464 | 1.02E-02 | 5.90E-02 | 0.049265 | NFKBIA/CXCL1 | 2 |
| hsa05207 | Chemical carcinogenesis - receptor activation | 2月7日 | 212/8464 | 1.21E-02 | 5.99E-02 | 0.05003 | KLF4/BCL6 | 2 |
| hsa05417 | Lipid and atherosclerosis | 2月7日 | 215/8464 | 1.24E-02 | 5.99E-02 | 0.05003 | NFKBIA/CXCL1 | 2 |

Supplementary Table 7. The KEGG results of keygenes.
